# SINTER3D: continuous 3D reconstruction of spatial transcriptomics via implicit neural representations

**DOI:** 10.1186/s13059-026-04160-5

**Published:** 2026-06-17

**Authors:** Tianjiao Zhang, Shenghe Li, Hongfei Zhang, Ruolan Zhang, Zhongqian Zhao, Ruihan Wang, Xiaopeng Teng, Long Wan, Yucai Jiang, Jianyi Lyu, Runqing Wang, Guohua Wang

**Affiliations:** 1 School of Computer Science and Artificial Intelligence, Northeast Forestry University, No. 26 Hexing Road, Xiangfang District, Harbin, 150040 China; 2 Faculty of Computing, Harbin Institute of Technology, No. 92 West Da Zhi Street, Nangang District, Harbin, 150001 China

**Keywords:** Spatial transcriptomics, Three-dimensional reconstruction, Implicit neural representation, Virtual section generation, Spatial domain identification, Cell type deconvolution, Multi-slice integration

## Abstract

Spatial transcriptomics enables gene-expression profiling while preserving spatial context, but three-dimensional reconstruction from discrete tissue sections remains limited by large inter-section gaps and gene-wise independent interpolation. We develop SINTER3D, an implicit neural representation–based framework for joint three-dimensional interpolation of multiple genes. SINTER3D models gene expression as continuous functions of three-dimensional coordinates, enabling virtual section generation, spatial-domain identification, and cell-type deconvolution. Across datasets including adult mouse brain, human dorsolateral prefrontal cortex, developing human heart, *Drosophila* embryo, and breast cancer tissues, SINTER3D outperforms existing methods and reconstructs biologically meaningful three-dimensional molecular structures.

**Supplementary Information:**

The online version contains supplementary material available at 10.1186/s13059-026-04160-5.

## Background

Elucidating the spatial organization principles governing cellular function within three-dimensional (3D) tissue architecture is crucial for understanding disease mechanisms and developmental processes [1]. Spatial transcriptomics has emerged as a transformative technology [2–4], enabling genome-wide expression profiling while preserving intact spatial information, thereby providing unprecedented resolution for studying tissue organization. With the increasing availability of multi-slice spatial transcriptomic datasets [5–8], the reconstruction of complete 3D transcriptomic atlases of tissues and organs has become feasible [9]. However, biological processes such as cell differentiation, morphogenesis, and organogenesis are inherently 3D phenomena [10]. The reliance of existing methods on discrete two-dimensional sections fundamentally limits our ability to capture the dynamic spatial variations within tissues.

Recent years have witnessed significant methodological advances for analyzing multi-slice spatial transcriptomics data. Early studies focused on developing section registration techniques: STUtility [11] employed the iterative closest point (ICP) algorithm for precise alignment of histological images and supported multi-section 3D visualization, while PASTE [12], based on fused Gromov-Wasserstein optimal transport theory, not only enables precise alignment of adjacent sections but also constructs stacked 3D tissue models. Building upon registration technologies, a new generation of joint analysis methods has achieved functional breakthroughs. STAGATE [13] utilizes a graph attention autoencoder framework to precisely characterize spatial domain boundaries by adaptively learning similarities between adjacent spots, and can integrate multiple consecutive sections to extract 3D expression domain information. GraphST [14] combines graph neural networks with a self-supervised contrastive learning strategy, encoding both gene expression and spatial proximity by minimizing the embedding distance between spatially adjacent spots, and enables multi-section joint analysis via registration algorithms. STitch3D [15] employs deep neural networks to directly model multiple sections jointly, detecting spatial domains and inferring cell type distributions in 3D space. In addition, recent methods such as SpaMask [16], SpaCross [17], SpaBatch [18], and mclSTExp [19] have further advanced spatial transcriptomics analysis from the perspectives of self-supervised spatial representation learning, cross-section integration, batch-effect correction, and multimodal expression prediction, respectively. These methods improve spatial-domain identification, cross-sample integration, or expression prediction; however, their primary objective is not to perform multi-gene joint interpolation at arbitrary coordinates or to generate virtual sections within a continuous three-dimensional tissue volume. Notably, Spateo [20], as a groundbreaking integrated framework, successfully reconstructed the 3D transcriptomic landscapes of mouse E9.5 and E11.5 embryos through scalable non-rigid registration and multi-section iterative optimization strategies, demonstrating the capability to construct "molecular holograms". By integrating gene expression patterns and spatial information across multiple sections, these methods significantly enhance the biological interpretability of 3D tissue structure analysis compared to single-section approaches.

However, the prevailing reconstruction paradigm based on stacking discrete sections faces fundamental challenges. The substantial unmeasured gaps between tissue sections lead to critical information loss [21]. In typical 3D reconstruction experiments for spatial transcriptomics, although individual section thickness is typically 10–20 µm [22], sparse sampling strategies are commonly adopted due to experimental costs, processing time, and sample throughput, collecting one section every few tens to hundreds of micrometers. This results in unsampled regions between adjacent measured sections equivalent to the thickness of dozens of cell layers, leading to several key limitations. Firstly, the absence of structural information within inter-slice gaps prevents the complete characterization of the spatial extension patterns of continuous anatomical features such as blood vessels, nerve tracts, and epithelial boundaries [21, 23]. Secondly, when section spacing is large, existing methods struggle to accurately infer consistent tissue structures and boundaries across wide gaps, resulting in reduced accuracy of 3D spatial domain identification [17, 21]. Although recent methods such as Spateo have incorporated diverse interpolation strategies—including Shepard and Gaussian interpolation in VTK, SparseVFC, and deep learning-based approaches—to enhance the continuity of 3D reconstruction, they still interpolate each gene independently. Consequently, these methods fail to capture the coordinated spatial variation and high-dimensional dependencies among genes that shape the organization and function of complex tissues. This single-gene independent modeling strategy is not only computationally inefficient, as it requires separate modeling for thousands of genes, but, more importantly, cannot ensure the biological consistency of the generated three-dimensional transcriptome at the system level. Therefore, the transition from a discrete stacking paradigm to truly continuous multi-gene joint reconstruction represents a central challenge in the field of three-dimensional tissue analysis. Recently, Luo et al. proposed STINR [24], which introduces implicit neural representation (INR) into spatial transcriptomics analysis by learning a continuous mapping from spatial coordinates to gene-expression patterns to characterize spatial expression variation. This work demonstrates the substantial potential of the INR framework for capturing continuous expression patterns in spatial transcriptomics. However, STINR is primarily designed for expression modeling and interpretation in two-dimensional spatial transcriptomics data. It does not explicitly integrate multiple tissue sections into a unified three-dimensional coordinate system, nor does it focus on interpolation across large inter-section gaps, virtual section generation, or continuous three-dimensional tissue reconstruction. Thus, how to leverage the INR framework to achieve continuous three-dimensional reconstruction from multi-section spatial transcriptomics data remains an open problem.

To address these challenges, we propose SINTER3D, an implicit neural representation–based framework that extends INR from two-dimensional spatial expression modeling to continuous three-dimensional reconstruction of multi-section spatial transcriptomics data, thereby enabling joint three-dimensional interpolation of multiple genes. Building upon existing registration methods, SINTER3D models preprocessed gene-expression profiles as continuous functions of three-dimensional spatial coordinates and learns the coordinated spatial distribution patterns of multiple genes within a unified framework, thereby supporting gene-expression queries at arbitrary three-dimensional coordinates. In addition, the framework enables joint three-dimensional spatial-domain identification across multiple sections and, when combined with single-cell reference data, supports cell-type deconvolution for low-resolution spatial transcriptomics data. The core innovation of SINTER3D lies in leveraging the implicit neural representation framework to learn a continuous mapping from spatial coordinates to gene expression, accurately capturing cross-section gene expression evolution and spatial structural variation patterns. It generates biologically plausible virtual sections to fill tissue gaps, thereby constructing a truly continuous 3D transcriptomic volume model. We systematically validated the performance of SINTER3D on datasets encompassing diverse tissue types and organism scales, including data generated from multiple spatial transcriptomics platforms such as 10 × Visium, ST, and Stereo-seq. Benchmarking comparisons against existing methods, including Spateo, demonstrate that SINTER3D excels in interpolation accuracy, 3D spatial domain identification, and 3D cell type deconvolution. As the first implicit neural representation–based framework for joint three-dimensional interpolation of multiple genes, SINTER3D provides a new methodological avenue for resolving the three-dimensional molecular organization of living systems.

## Results

### Overview of SINTER3D

**Fig. 1:**
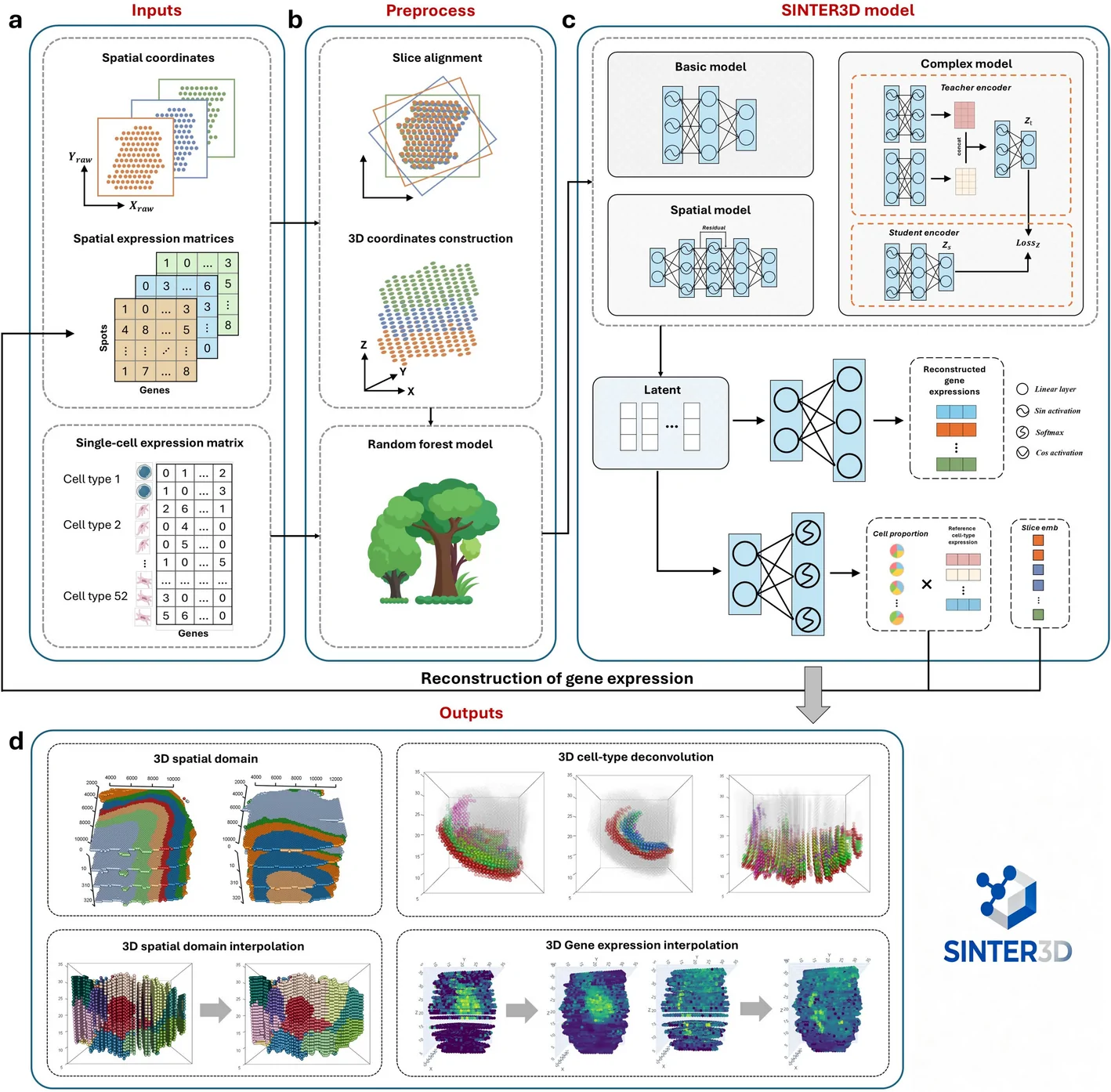
Overview of SINTER3D. The raw inputs for SINTER3D comprise multi-slice spatial transcriptomics (ST) tissue sections and cell-type-specific gene expression profiles from a reference single-cell RNA sequencing (scRNA-seq) dataset. Preprocessing steps in SINTER3D involve aligning spots from different tissue sections to construct a unified 3D coordinate system, which are then input into a pre-trained random forest model. SINTER3D constructs three distinct sub-models tailored to different dataset characteristics. A pre-trained random forest model selects the optimal sub-model in a data-driven manner. The framework subsequently jointly models multiple slices by employing an INR to continuously represent ST data, which implicitly characterizes the correlation between spatial coordinates and gene expression across sections. Simultaneously, an encoder-decoder architecture leverages the single-cell reference data to deconvolve the ST data. By integrating these components, SINTER3D constructs a mapping from spatial coordinates to gene expression, consequently enabling gene expression interpolation at any arbitrary 3D spatial position, identification of 3D spatial domains, and 3D cell type deconvolution. The outputs of SINTER3D include the identified 3D spatial domains, the reconstructed 3D spatial distribution of distinct cell types within the tissue, the interpolated gene expression values filling the gaps between physical sections, and the subsequent spatial domain annotation derived from the complete 3D reconstruction

SINTER3D is a deep learning method based on an implicit neural representation (INR) framework, designed to reconstruct three-dimensional (3D) tissue structures from multiple two-dimensional (2D) spatial transcriptomics (ST) slices (Fig. 1). The inputs to SINTER3D include multiple ST slices and a matched single-cell RNA sequencing (scRNA-seq) reference (Fig. 1a). A key preprocessing step involves aligning these slices to construct a unified 3D spatial coordinate system. Subsequently, a pre-trained random forest model selects the most appropriate sub-model based on the specific characteristics of the input data (Fig. 1b).

Specifically (Fig. 1c), the core of SINTER3D leverages an implicit neural network to learn a continuous mapping from 3D spatial coordinates (incorporating a slice index encoding to integrate information across multiple sections) to a latent representation that captures the underlying gene expression patterns. A separate neural network is introduced to decode cell type proportions directly from this latent representation. SINTER3D is then trained to reconstruct the observed ST gene expression by combining the estimated cell type proportions with the cell-type-specific gene expression profiles from the scRNA-seq reference.

Upon completion of training, SINTER3D effectively learns a continuous function that maps any 3D spatial coordinate to its corresponding latent gene expression representation and cell type composition. This learned model enables the prediction of gene expression profiles at any arbitrary, previously unmeasured 3D location, facilitating high-fidelity interpolation within the tissue volume. The coherent latent representations can be clustered to identify biologically meaningful 3D spatial domains, while the decoded cell type proportions enable the reconstruction of the 3D spatial distribution of cell types (Fig. 1d). Together, these outputs support a wide range of downstream analyses. Comprehensive methodological details are provided in the Methods section.

### Comprehensive evaluation of SINTER3D on the structurally complex 3D adult mouse brain dataset

**Fig. 2:**
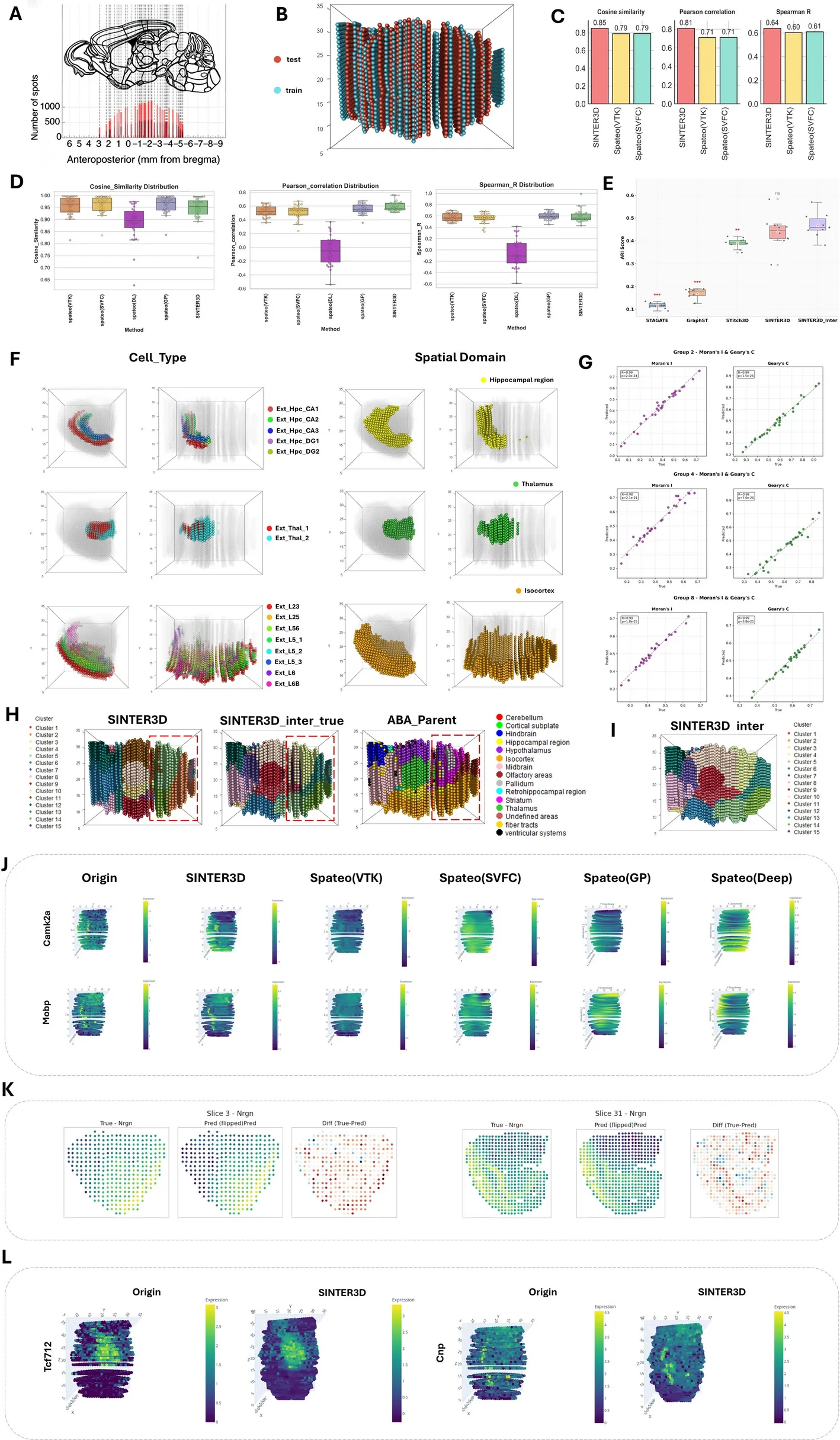
3D reconstruction of the adult mouse brain. **A** The adult mouse brain dataset generated by the ST platform [5]. **B** Thirty-five coronal sections distributed along the anterior–posterior (AP) axis used in this experiment. **C** Quantitative comparison between SINTER3D and the VTK and SparseVFC (SVFC) interpolation methods provided by Spateo. **D** Performance comparison between SINTER3D and all interpolation methods from Spateo on a selected set of 30 highly variable genes (HVGs). **E** Comparison of the Adjusted Rand Index (ARI) for spatial domain identification between SINTER3D and several baseline methods. **F** Visualization of the 3D cell type distribution and 3D spatial domains identified by SINTER3D. **G** Spatial correlation analysis between the data interpolated by SINTER3D and adjacent real tissue sections. **H** Comparison of 3D spatial domain identification results before and after interpolation using SINTER3D. **I** The complete 3D spatial domain model after SINTER3D interpolation. **J** Comparison between the original gene expression in the test set and the results after interpolation using various methods. **K** Gene expression interpolation results by SINTER3D on two-dimensional sections. **L** Overall dataset comparison before and after SINTER3D interpolation

We first systematically evaluated the overall performance of SINTER3D on a structurally complex three-dimensional (3D) adult mouse brain dataset. This dataset comprises 35 coronal sections distributed along the anterior–posterior (AP) axis [5] (Fig. 2A, B), accompanied by annotations for 59 reference cell types [25], presenting a significant challenge for the model in terms of capturing cross-section data consistency and spatial distribution variations of cell subtypes.

In an odd–even section split experiment (18 odd-numbered sections for training, 17 even-numbered sections for validation) (Fig. 2B), SINTER3D significantly outperformed the VTK (Shepard) and SparseVFC (SVFC) interpolation methods provided by Spateo in terms of global interpolation performance on the validation set (Fig. 2C and Additional file 1: Fig. S1). Specifically, SINTER3D achieved a Cosine similarity of 0.85 (compared to 0.79 for both VTK and SVFC), a Pearson correlation coefficient of 0.81 (compared to 0.71 for both), and a Spearman correlation coefficient of 0.64 (compared to 0.60 for VTK and 0.61 for SVFC). Due to the training time constraints of the Gaussian Process (GP) and deep learning methods provided by Spateo, which prevented a direct comparison on the full gene set, we evaluated performance on 30 selected highly variable genes (HVGs). The results (Fig. 2D, J) showed that while most methods, except Spateo's deep learning method, achieved Cosine similarities exceeding 0.90, SINTER3D demonstrated superior performance in both Pearson and Spearman correlations. It is noteworthy that SINTER3D performs joint interpolation across all preprocessed HVGs, leveraging gene co-expression patterns to enhance prediction accuracy, whereas Spateo only supports gene-by-gene independent interpolation, unable to capture synergistic information between genes, which is a key factor limiting its performance.

To validate the spatial correlation fidelity of the data interpolated by SINTER3D, we grouped the 35 sections, with each group containing one test section and its two adjacent training sections. We calculated Moran's I and Geary's C for the real gene expression in the test section versus the gene expression in the adjacent training sections, and compared these metrics with those computed for the interpolated data. The results (Fig. 2G) showed that the correlation between the real and interpolated data exceeded 0.95 for both metrics.

To assess whether the interpolation results possess genuine biological relevance, we further visualized the 2D and 3D spatial distributions of several HVGs on the test set (Fig. 2J, K, and Additional file 1: Figs. S2, S3). These genes are closely associated with the regulation of excitatory and inhibitory neuronal functions in the mouse brain, involved in synaptic plasticity, signal transduction, neurodevelopment, and metabolic processes, thereby influencing learning and memory, neural network equilibrium, motor control, the reward system, and susceptibility to psychiatric disorders. Comparing the original data with the interpolated data, we found that SINTER3D accurately reconstructed complex spatial expression patterns and effectively reduced technical noise present in the sequencing data, resulting in smoother expression within the same regions and sharper regional boundaries.

Based on the deconvolution results from SINTER3D (Fig. 2F), *Ext_Hpc* cell types showed high spatial consistency with the hippocampal region; *Ext_Thal* cell types aligned well with the thalamic region; and *Ext_L* cell types, associated with the cerebral cortex, accurately depicted the spatial distribution across different cortical areas in the 3D view, highly consistent with the layout of the isocortex region.

We further employed SINTER3D to interpolate and reconstruct the gaps between the original 35 sections. The results (Fig. 2L and Additional file 1: Fig. S4) demonstrated that SINTER3D successfully reconstructed the complete 3D spatial expression patterns of key genes such as *Tcf7l2*, *Cnp*, and *Mbp* [26], based on both the original and interpolated data. These genes are involved in crucial biological processes like neurodevelopmental regulation, and myelination formation and maintenance. The accurate reconstruction of their 3D distribution patterns provides important insights for understanding functional specialization across brain regions. In addition, to further verify whether SINTER3D can support super-resolution reconstruction within the same physical section, we generated new virtual spots between measured spots and used the trained SINTER3D model to infer gene expression at these newly added coordinates. The strategy for generating virtual spots was designed according to the array structure of each platform and is described in detail in the Methods section. This analysis achieved approximately 3.9-fold spatial densification in the DLPFC Visium dataset and 3.6- to 3.8-fold spatial densification in the adult mouse brain ST dataset. Visualization of representative marker genes showed that the super-resolution reconstructed expression patterns exhibited strong spatial continuity and remained consistent with the anatomical structures and regional expression patterns observed in the original sections (Additional file 1: Figs. S5–S7). These results indicate that SINTER3D is capable not only of inter-section interpolation along the z-axis, but also of super-resolution expression prediction at unmeasured spatial locations within the same section.

Using both the original data and the data interpolated by SINTER3D, we performed spatial domain detection, utilizing the ABA_parent annotations from the Allen Brain Atlas (ABA) as the gold standard [27]. The results (Fig. 2E) showed that SINTER3D's spatial domain identification performance before interpolation was already superior to various baseline methods (mean ARI = 0.44, significantly higher than STitch3D [15] at 0.39, GraphST [14] at 0.16, and STAGATE [13] at 0.11). This performance improved further after interpolation to an ARI of 0.47. To further validate the biological validity of the interpolated data, we compared the spatial domain identification results for the test set (interpolated even-numbered sections) against the results obtained using all real sections. The results indicated that the ARI for the test set was slightly lower than that for the real data (Additional file 1: Fig. S8), a minor decrease theoretically expected due to the inevitable slight information loss during interpolation. Crucially, the results on the interpolated test set still significantly outperformed all baseline methods and showed high consistency with the ground truth (ABA annotations), fully demonstrating that SINTER3D's interpolated data preserve the spatial biological characteristics of the original data. 3D visualization analysis indicated that spatial domain identification for the first ten sections in the dataset was improved after interpolation compared to before. In the 2D section view (e.g., sections 14 A, 20B), identification of the Isocortex region became more precise post-interpolation, showing high agreement with ABA annotations (Additional file 2: Fig. S9).

In summary, SINTER3D demonstrated excellent performance and high spatial biological fidelity across multiple tasks including gene expression interpolation, spatial domain detection, and cell type deconvolution, offering potential for the construction of multimodal 3D brain atlases.

### SINTER3D enables integration and analysis of 3D spatial transcriptomes in a widely-spaced DLPFC dataset

**Fig. 3:**
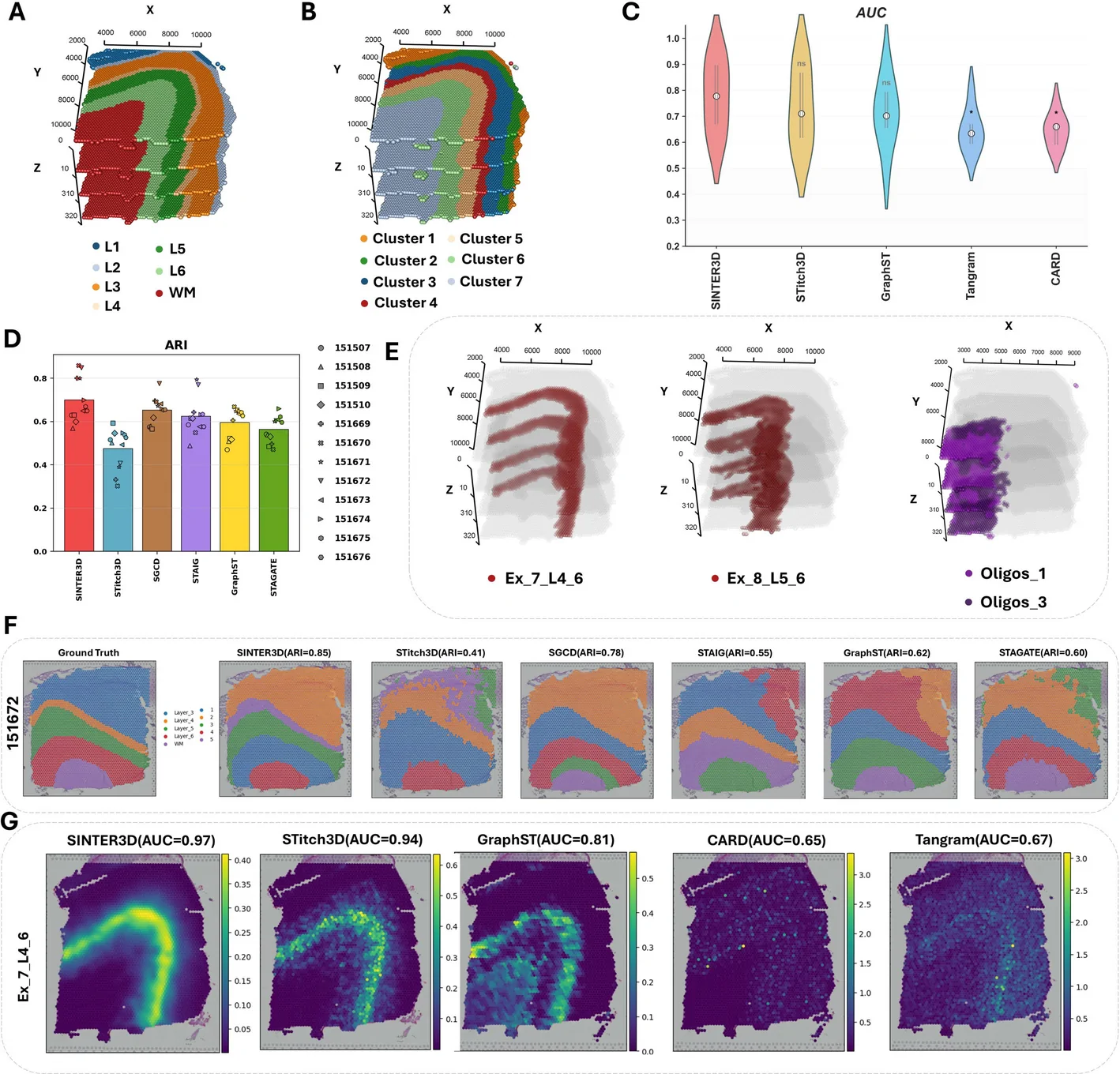
Integration and analysis of 3D spatial transcriptomes using SINTER3D on a widely-spaced DLPFC dataset. **A** Ground truth labels of 3D spatial domains across four sections (including #151,673). **B** 3D spatial domain identification results generated by SINTER3D. **C** Violin plots comparing AUC scores between SINTER3D and baseline methods. **D** Performance comparison of spatial domain identification between SINTER3D and baseline methods across all 12 sections. **E** 3D visualization of cell type distributions reconstructed by SINTER3D. **F** Spatial domain identification results on section #151,672: SINTER3D versus baseline methods. **G** Comparative analysis of *Ex_7_L4_6* cell type identification on section #151,674 between SINTER3D and baseline methods

We systematically evaluated the performance of SINTER3D across three critical tasks—spatial domain identification, cell type deconvolution, and wide-interval section interpolation—using the human dorsolateral prefrontal cortex (DLPFC) dataset [6]. This dataset comprises 12 sections from 3 individuals (4 sections per individual), with each section previously annotated for 4–6 cortical layers (L1–L6) and the white matter (WM) region in the original study (Fig. 3A). Given the substantial physical intervals between sections in this dataset, it provides an ideal scenario for assessing SINTER3D's capability to handle sparsely sampled 3D tissues.

We first performed spatial domain identification on the three sample groups separately (Fig. 3D and Additional file 2: Fig. S10). The spatial domains identified by SINTER3D showed high consistency with the manually annotated laminar architecture. Quantitative evaluation employed the Adjusted Rand Index (ARI) as the accuracy metric, using manual annotations as the gold standard. SINTER3D achieved an average ARI of 0.70 across all sections, significantly outperforming the next best method, SGCD [28] (0.65), and other baseline methods, including STAIG [29] (0.62), GraphST [14] (0.60), STAGATE [13] (0.56), and STitch3D [15] (0.48). Notably, SINTER3D demonstrated particularly outstanding performance on sections 151,671 and 151,672 (Fig. 3F and Additional file 2: Fig. S11), achieving ARIs of 0.80 and 0.85, respectively, substantially surpassing all other methods. On section 151,671, only SINTER3D and STAIG successfully identified Layer 4; whereas on section 151,672, SINTER3D was the only method capable of accurately identifying Layer 4. This advantage stems from SINTER3D's effective integration of multi-section information, successfully constructing a continuous mapping function from spatial coordinates to latent representations, thereby capturing the intrinsic 3D spatial patterns within the dataset.

To evaluate cell type deconvolution performance, we utilized a single-cell reference dataset containing 10 excitatory neuronal subtypes, with the layer specificity of each subtype determined via layer marker genes. We employed Receiver Operating Characteristic (ROC) analysis to assess deconvolution reliability, using the estimated neuronal subtype proportions to predict layer-specific distributions, where a higher Area Under the Curve (AUC) indicates better performance. A systematic comparison between SINTER3D and mainstream deconvolution methods (STitch3D, GraphST, Tangram [30], and CARD [31]) revealed its significant superiority (Fig. 3G). On consecutive sections starting from 151,673, SINTER3D achieved an average AUC of 0.78, demonstrating stable performance improvements compared to STitch3D (0.73), GraphST (0.71), Tangram (0.65), and CARD (0.64). This advantage was particularly pronounced in the spatial localization of individual cell subtypes. Taking the excitatory neuronal subtype *Ex_7_L4_6* in section 151,674 as an example, this subtype specifically expresses layer marker genes in layers 4–6. SINTER3D successfully reconstructed its enriched distribution pattern in the corresponding cortical layers, achieving a notably high AUC of 0.972, significantly surpassing STitch3D (0.94), GraphST (0.81), Tangram (0.65), and CARD (0.67). More importantly, the 3D spatial reconstruction results further corroborated SINTER3D's exceptional deconvolution performance (Fig. 3E). We observed that several layer-specific cell subtypes—including *Ex_7_L4_6*, *Ex_8_L5_6*, *Oligos_1*, and *Oligos_3*—exhibited highly consistent 3D spatial distributions with the anatomical locations of their corresponding cortical domains, fully demonstrating SINTER3D's ability to precisely resolve the spatial organization patterns of cell types in three dimensions while maintaining the continuity and integrity of the histological structure.

Finally, we selected the four most commonly used sections in the DLPFC dataset (#151,673, #151,674, #151,675, #151,676) to systematically evaluate the interpolation performance of SINTER3D (Additional file 2: Figs. S12-S16). These sections present varying spatial interval characteristics: the intervals between #151,673 and #151,674, and between #151,675 and #151,676, are 10 μm, whereas a significantly larger 300 μm interval exists between #151,674 and #151,675. This cross-scale interval distribution provides ideal experimental conditions for comprehensively evaluating the model's interpolation capability across different spatial resolutions. Based on this, we designed two complementary experiments to assess SINTER3D's performance in interpolation and extrapolation scenarios, respectively. Each experiment employed the same evaluation strategy: using training sections to build the SINTER3D model, performing data interpolation at test anchor positions, and quantitatively assessing the biological fidelity of the interpolated data via the ARI of spatial domain identification.

In the interpolation experiment, we used the outermost sections #151,673 and #151,676 as training slices to perform interpolation predictions for the intervening sections #151,674 and #151,675. The results (Additional file 3: Fig. S17) showed high consistency between the spatial domains identified from the interpolated data and the ground truth annotations, with ARIs reaching 0.62 and 0.63 for #151,674 and #151,675, respectively. Although this performance is slightly lower than SINTER3D's identification accuracy on real observed data (0.70 and 0.67), it still significantly surpasses most baseline methods, including STitch3D (0.55 and 0.54), STAIG (0.53 and 0.52), and STAGATE (0.57 and 0.43), fully validating SINTER3D's robustness and accuracy in large-interval interpolation scenarios.

In contrast, the extrapolation experiment used the middle sections #151,674 and #151,675 as training slices to perform extrapolative interpolation for the outer sections #151,673 and #151,676. As expected, the extrapolation performance decreased compared to interpolation, with ARIs of 0.61 and 0.59 for #151,673 and #151,676, respectively, but still outperformed STitch3D (0.49 and 0.53), STAIG (0.53 and 0.53), and STAGATE (0.60 and 0.58). This performance difference aligns with fundamental principles of computational modeling and biology: extrapolation inherently involves inference in unknown spaces outside the model's training domain, carrying greater predictive uncertainty than interpolation within the training domain. More importantly, the large 300 μm interval might contain histological structural transitions, changes in cellular composition, or microenvironment heterogeneity not captured by the training sections—potential biological complexities that further increase the difficulty of the extrapolation task. Nonetheless, SINTER3D demonstrated performance superior to most existing methods in both scenarios, proving its technical advantage in handling sparsely sampled 3D data.

In summary, SINTER3D achieved exceptional capabilities in spatial domain identification, cell type deconvolution, and cross-interval section interpolation on the DLPFC dataset, providing a powerful computational tool for the integrated analysis and biological interpretation of 3D spatial transcriptomic data.

### SINTER3D deciphers spatial functional domains and coordinated cellular distributions in early human heart development

**Fig. 4:**
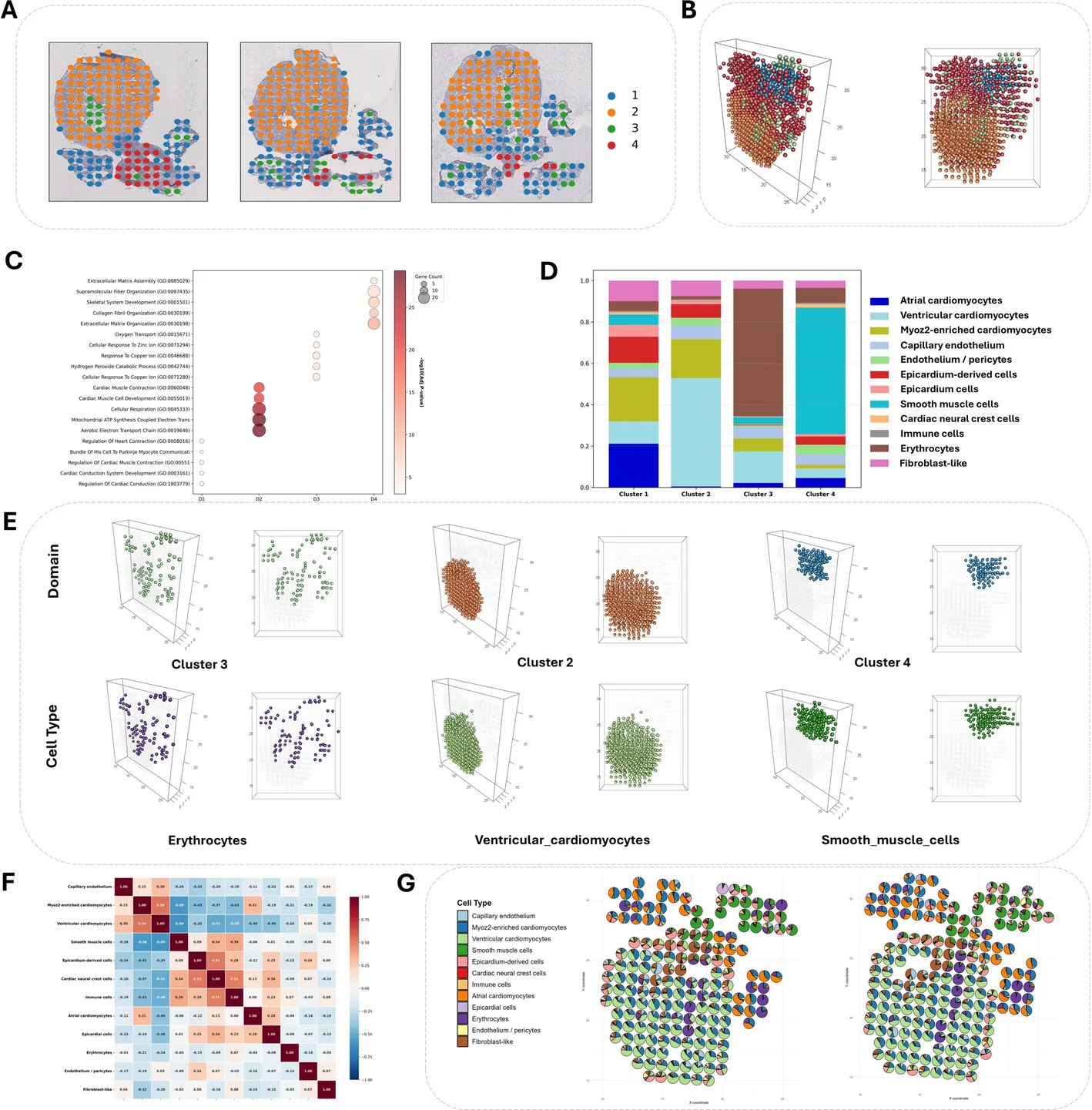
Analysis of the developing human heart Dataset. **A** Representative spatial domain identification results by SINTER3D on selected sections. **B** Three-dimensional architectural reconstruction of the developing human heart generated by SINTER3D. **C** Gene Ontology (GO) enrichment analysis of biological processes for identified spatial domains, showing representative terms. **D** Distribution relationships between spatial domains and major cell types. **E** Spatial correspondence between 3D domains and 3D cell type distributions after reconstruction. **F** Cell type co-localization heatmap depicting pairwise spatial associations. **G** Pie charts visualizing cell type proportions within a representative tissue section

We applied SINTER3D to a spatial transcriptomics (ST) dataset of the human embryonic heart, sourced from a 6.5 post-conception weeks (6.5-PCW) human heart sample [7]. Through SINTER3D analysis, we robustly identified four distinct spatial domains across nine consecutive sections (Fig. 4A and Additional file 3: Fig. S18). To elucidate the functional characteristics of these domains, we performed Gene Ontology (GO) enrichment analysis [32] (Fig. 4C). The results revealed that Spatial Domain 1 corresponds to the cardiac conduction system region, with its significantly enriched GO terms primarily associated with biological processes related to cardiac conduction system development and electrical signal transduction [33]. Spatial Domain 2 corresponds to the working myocardium region (primarily ventricular myocardium), significantly enriched in processes related to cardiac muscle contraction and energy metabolism [34]. Spatial Domain 3 corresponds to the cardiac vascular system region, enriched with terms related to gas transport—including oxygen and carbon dioxide transport—and cellular response to copper and zinc ions [35]. Spatial Domain 4 corresponds to the cardiac fibrous skeleton and valve regions, with its enriched GO terms mainly associated with structural biological processes such as extracellular matrix organization, collagen fibril organization, and elastic fiber assembly [36].

Notably, SINTER3D enabled precise reconstruction of the 3D spatial distribution of cell types (Fig. 4B), which exhibited a high degree of correspondence with the aforementioned spatial domains (Fig. 4E). Specifically, Spatial Domain 2 was predominantly composed of ventricular cardiomyocytes, whose enriched functions in muscle contraction and mitochondrial ATP synthesis closely align with the physiological characteristics of working myocardium. Spatial Domain 3 was significantly enriched in erythrocytes, consistent with the gas transport function of this domain, indicating its correspondence to the cardiac chambers and vascular lumens. Spatial Domain 4 showed spatial co-localization with smooth muscle cells [37], reflecting the structural composition of the coronary vessel walls and cardiac fibrous skeleton.

Further cell type co-localization analysis revealed significant spatial interaction patterns (Fig. 4F, G): *Myoz2*-enriched cardiomyocytes exhibited strong spatial co-localization with ventricular cardiomyocytes [7], suggesting the former may represent a functional subtype of ventricular muscle. Furthermore, significant spatial associations were observed between epicardium-derived cells and cardiac neural crest cells, as well as between immune cells and cardiac neural crest cells [38], reflecting coordinated distribution patterns of specific cell subpopulations during heart development and their potential roles in shaping the cardiac immune microenvironment.

In summary, SINTER3D successfully integrates functional domain identification from spatial transcriptomic data with 3D spatial reconstruction of cell types, providing a powerful tool for deepening the understanding of the spatial organizational principles and cell–cell interaction patterns during early human heart development.

### SINTER3D enables high-fidelity reconstruction of the 3D spatial transcriptomic atlas in highly sparse *Drosophila* embryos

**Fig. 5:**
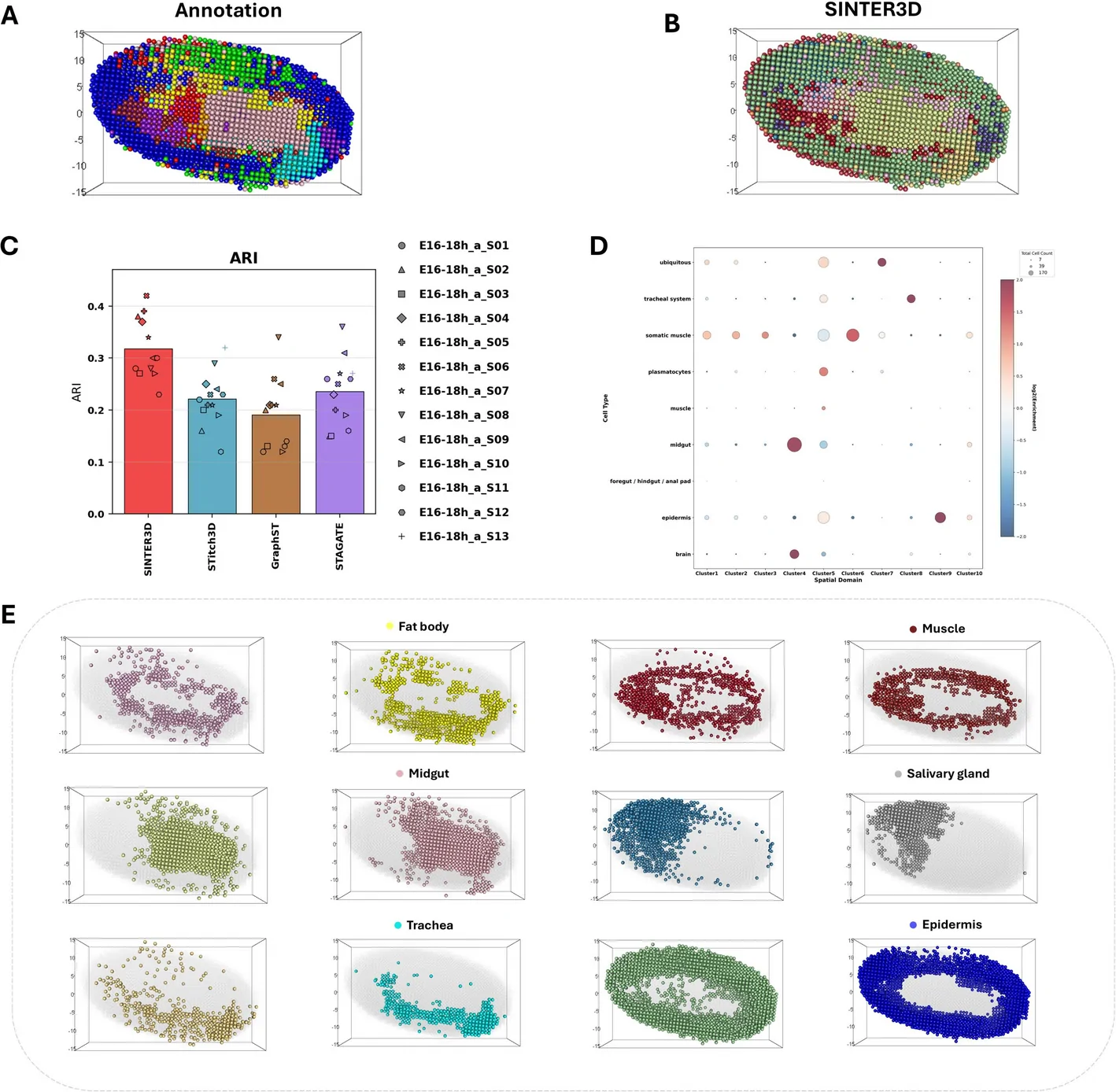
Reconstruction of the single-cell-resolution *Drosophila* embryo dataset. **A** Ground truth 3D atlas of the *Drosophila* embryo dataset. **B** Reconstruction of the *Drosophila* embryo dataset by SINTER3D. **C** Comparison of spatial domain identification performance between SINTER3D and baseline methods across all 13 sections. **D** Bubble plot illustrating the distribution relationships between spatial domains and cell types. **E** Visual comparison between the 3D spatial domains identified by SINTER3D and the ground truth annotations

We applied SINTER3D to reconstruct a comprehensive three-dimensional spatial atlas of the whole *Drosophila* embryo, demonstrating its capability in integrating large-scale spatial transcriptomics data. Our analysis utilized 16–18 h embryo samples sequenced by Stereo-seq [8] (Fig. 5A and Additional file 3: Fig. S19). To establish a cell type reference for SINTER3D analysis, we performed cell type annotation based on a scRNA-seq atlas of *Drosophila* embryos at the same developmental stage [39]. This task presented significant technical challenges due to the pronounced sparsity of the *Drosophila* embryonic ST data. Despite these difficulties, SINTER3D successfully reconstructed the complete 3D spatial architecture of the *Drosophila* embryo through effective integration of ST and scRNA-seq data (Fig. 5B). To evaluate reconstruction accuracy, we conducted a systematic comparative analysis between spatial domains identified by SINTER3D and those determined by other benchmark methods (Fig. 5C). In evaluations using the Adjusted Rand Index (ARI) across 13 sections, SINTER3D achieved a score of 0.31, significantly outperforming STitch3D (0.22), GraphST (0.19), and STAGATE (0.23), thereby highlighting the superiority of our method. A comprehensive bubble plot illustrating the correspondence between cell type distributions and spatial domains identified by SINTER3D (Fig. 5D) revealed distinct tissue-specific enrichment patterns across different spatial domains [8].

From a global spatial organization perspective, SINTER3D accurately captured the three-dimensional organizational characteristics of the *Drosophila* embryo. Along the anterior–posterior axis [40], spatial domains exhibited a progressive distribution pattern: the head and foregut region (Spatial Domain 8, enriched for foregut cells), the central midgut and nervous system region (Spatial Domains 3/4, enriched for brain and intestinal cells), the trunk muscle and mesoderm region (Spatial Domains 5/6, co-enriched for muscle and hemocytes), and the tail and hindgut region (Spatial Domain 9, enriched for hindgut cells). Along the radial axis [41], the tissue organization displayed a characteristic inner-middle-outer stratification: the inner layer comprised intestinal tissue (Spatial Domain 4), the middle layer contained muscle and hemocyte-enriched regions (Spatial Domains 5/6), and the outer layer consisted of epidermal cell-enriched regions (Spatial Domains 1/7). This reconstruction fully reveals the germ layer differentiation pattern and 3D spatial organization characteristics of the *Drosophila* embryo. We observed high concordance between the 3D spatial domains identified by SINTER3D and the ground truth labels (Fig. 5E), with successful identification and accurate reconstruction of typical tissue regions including fat body, midgut, trachea, muscle, salivary gland, and epidermis [8], demonstrating the high fidelity of SINTER3D in resolving complex biological tissue structures.

These results indicate that SINTER3D not only processes highly sparse spatial transcriptomics data effectively, but also precisely reconstructs the spatial architecture of complex whole organisms in three dimensions while recovering their germ layer differentiation patterns. Through our multimodal data integration approach, we successfully reconstructed a biologically meaningful 3D embryonic model from 2D slice-based spatial transcriptomics data, fully demonstrating the strong potential and broad application prospects of SINTER3D in large-scale, high-complexity spatial transcriptome analysis of biological systems.

Notably, although the *Drosophila* embryo dataset was generated using the Stereo-seq platform, this study used the cell-segmented expression matrix provided by the original study, in which each spatial unit corresponds to a segmented cell. Therefore, this result also demonstrates that SINTER3D can process spatial transcriptomics data at single-cell resolution. To further validate this capability, we applied SINTER3D to the STARmap PLUS single-cell-resolution mouse brain dataset [42]. The results showed that SINTER3D recovered spatial structures consistent with the ground-truth cell-type annotations in two 13-month-old TauPS2APP disease replicate samples, and achieved stable performance under both ICP and CAST [43] alignment strategies (Additional file 3: Fig. S20 and Additional file 3: Table S1). These results further support the applicability of SINTER3D to single-cell-resolution spatial transcriptomics data.

### SINTER3D enables deciphering of functional heterogeneity in 3D spatial transcriptomics of breast tumors

**Fig. 6:**
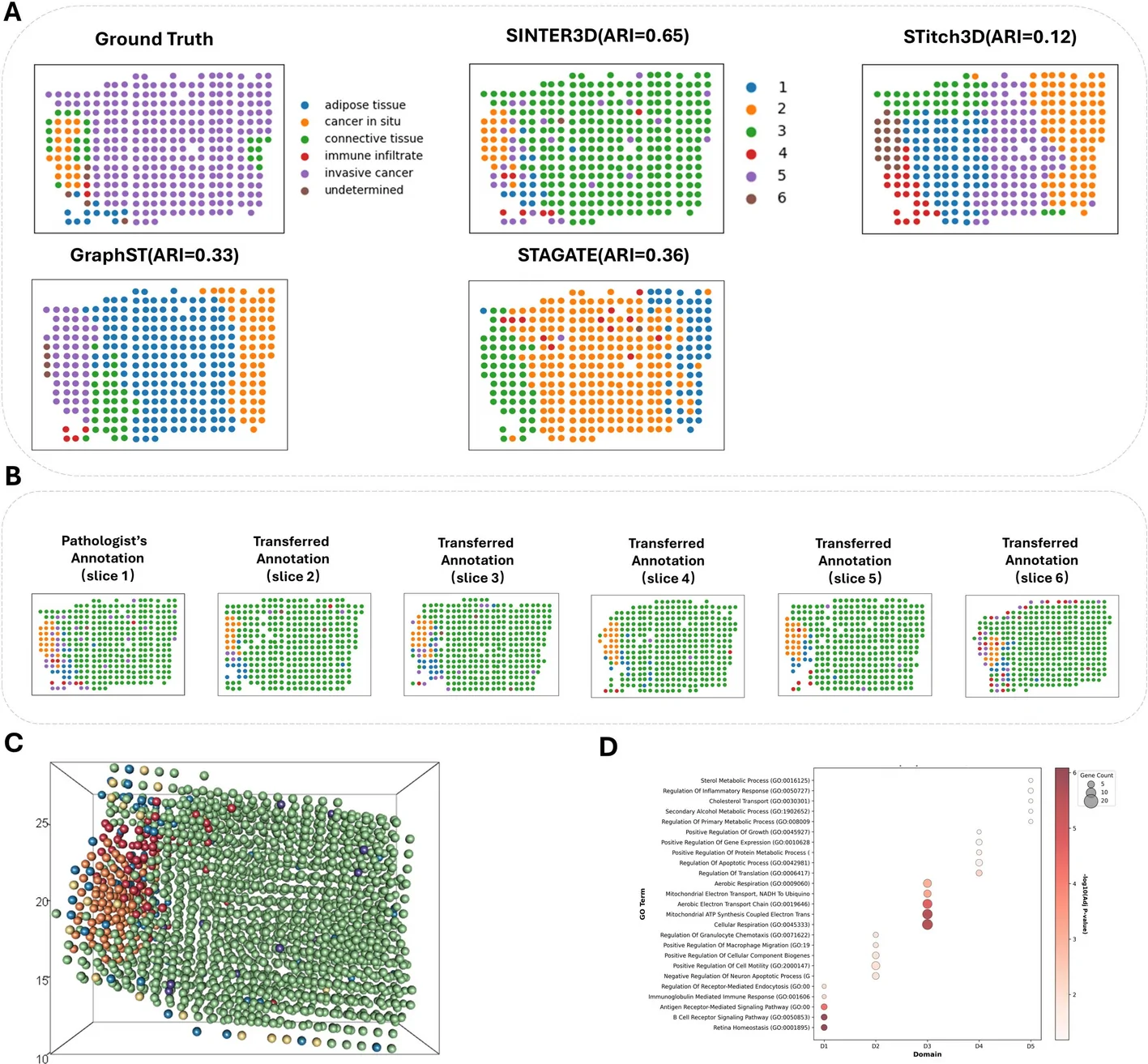
Analysis of HER2-positive breast cancer data. **A** Comparison of spatial domain segmentation results between SINTER3D and baseline methods on the first tissue section. **B** Spatial domains identified by SINTER3D across all six consecutive sections. **C** 3D reconstruction of the breast cancer dataset by SINTER3D. **D** GO enrichment analysis across the identified spatial domains

To demonstrate the potential of SINTER3D in deciphering the 3D architecture of abnormal tissues, we analyzed 3D spatial transcriptomic data from a breast cancer patient [7, 44], comprising a total of 2,065 spatial spots across six consecutive tissue sections. Applying SINTER3D, we identified six biologically coherent 3D spatial domains within the tumor tissue. Since the first section had been histologically annotated by pathologists, we systematically compared the spatial domains identified by SINTER3D on this section against several benchmark methods (Fig. 6A). Quantitative evaluation revealed that SINTER3D achieved an Adjusted Rand Index (ARI) of 0.65, significantly outperforming STitch3D (0.12), GraphST (0.33), and STAGATE (0.36), thereby demonstrating the superior performance of our method.

Based on GO enrichment analysis (Fig. 6D), the spatial domains identified by SINTER3D exhibited significant functional heterogeneity and distinct biological characteristics. Spatial Domain 3 was enriched for pathways related to mitochondrial energy metabolism, including aerobic respiration, the electron transport chain, and ATP synthesis, reflecting the characteristic high metabolic activity of invasive cancer cells [45]. Spatial Domain 1 was enriched for immune response-related signaling pathways, such as the B cell receptor signaling pathway, antigen receptor-mediated signaling, and immunoglobulin-mediated immune response, indicating its correspondence to an immune cell-infiltrated region [46]. Spatial Domain 2 was enriched for functions related to cell migration and chemotaxis, including the positive regulation of macrophage migration, regulation of granulocyte chemotaxis, and positive regulation of cell motility, suggesting its potential location at the tumor-immune interface or within stromal regions. Spatial Domains 4 and 5 were primarily associated with processes such as growth regulation, protein metabolism, and regulation of gene expression, exhibiting lower metabolic activity, and likely corresponding to carcinoma in situ or adjacent normal tissue regions [47].

Importantly, SINTER3D successfully identified spatial domains across all six sections simultaneously (Fig. 6B). These domains demonstrated high consistency across multiple sections, and their 3D visualization robustly confirmed the accuracy and reliability of SINTER3D in reconstructing 3D tissue structures from serial sections (Fig. 6C).

These results indicate that SINTER3D not only significantly surpasses existing methods in the accuracy of spatial domain identification but also effectively delineates 3D spatial domains with clear biological functional characteristics. This provides a powerful analytical tool for gaining deeper insights into the spatial heterogeneity of the tumor microenvironment and its role in cancer progression.

### Ablation studies

To systematically validate the contribution of individual components within SINTER3D, we conducted comprehensive ablation studies across three spatial transcriptomics datasets of varying complexity: the DLPFC dataset (sections #151,673–76), the mouse brain dataset, and the *Drosophila* embryo dataset, representing basic, spatial, and complex model scenarios, respectively. We designed five ablation configurations, sequentially removing the Poisson negative log-likelihood loss (w/o Poisson Loss), the intermediate feature loss (w/o Mid-Feat Loss), the reconstruction loss (w/o Recon Loss), the multi-scale spatial encoder (w/o Spatial_multi_scale), and the teacher-student distillation module (w/o T-S_model). All experiments were evaluated using the Adjusted Rand Index (ARI) to assess the model's capability in capturing biological structures.

**Fig. 7:**
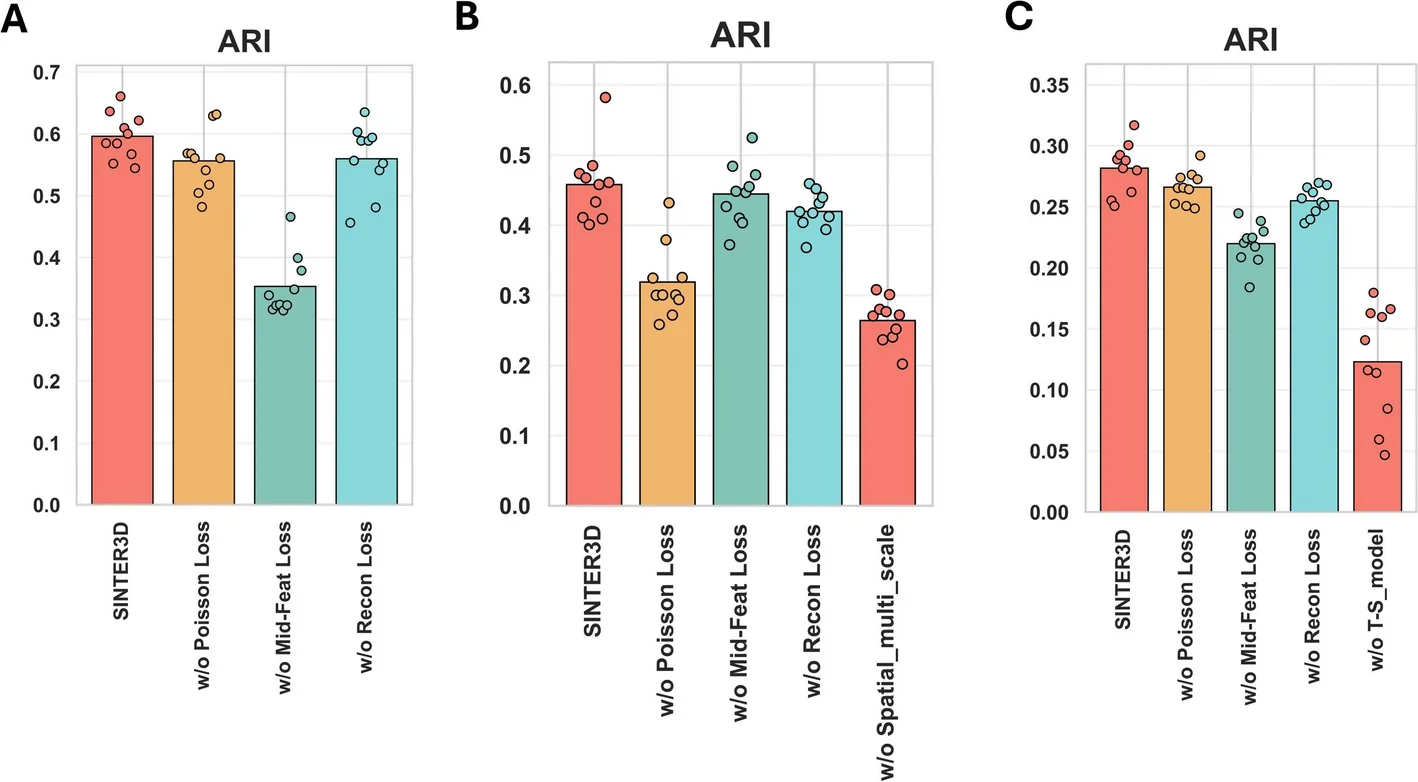
Ablation studies. **A** Ablation study on the basic model using the DLPFC dataset. **B** Ablation study on the spatial model using the mouse brain dataset. **C** Ablation study on the complex model using the *Drosophila* embryo dataset

On the DLPFC dataset, the full model achieved a mean ARI of 0.58 (Fig. 7A). Removing the intermediate feature loss resulted in a substantial performance drop (~ 40% decrease), underscoring the critical role of multi-level feature alignment in deep representation learning. In contrast, the removal of either the Poisson loss or the reconstruction loss had a comparatively minor impact, suggesting that discriminative learning dominates model performance on structurally simpler datasets.

Results on the mouse brain dataset highlighted the importance of spatial modeling. The full model attained an ARI of 0.47 (Fig. 7B). Removing the multi-scale spatial encoder caused a drastic performance reduction (~ 43% decrease), emphasizing the essential role of multi-scale spatial information integration for capturing tissue microenvironment complexity. Omitting the Poisson loss decreased the ARI to 0.32 (a 32% reduction), reflecting the importance of accurately modeling gene expression distributions in complex spatial organizations. The effects of removing the intermediate feature loss and reconstruction loss were more moderate.

Using the most challenging *Drosophila* embryo dataset, the full model achieved an ARI of 0.29 (Fig. 7C). Removing the teacher-student module led to a sharp performance decline to 0.13 (a 55% decrease), robustly demonstrating the core value of knowledge distillation in handling highly complex spatial patterns. Removing other components also resulted in performance degradation: w/o Mid-Feat Loss (ARI = 0.23), w/o Recon Loss (ARI = 0.25), and w/o Poisson Loss (ARI = 0.27).

Cross-dataset analysis revealed three key findings. First, the intermediate feature loss consistently demonstrated importance across all scenarios, validating the universal value of hierarchical feature learning. Second, the contribution of specific components exhibited a graded dependence on data complexity: the basic dataset relied more on discriminative learning, the spatial dataset required multi-scale encoding, and the complex dataset necessitated knowledge distillation, highlighting the adaptive capability of SINTER3D's modular design.

### Parameter analysis

**Fig. 8:**
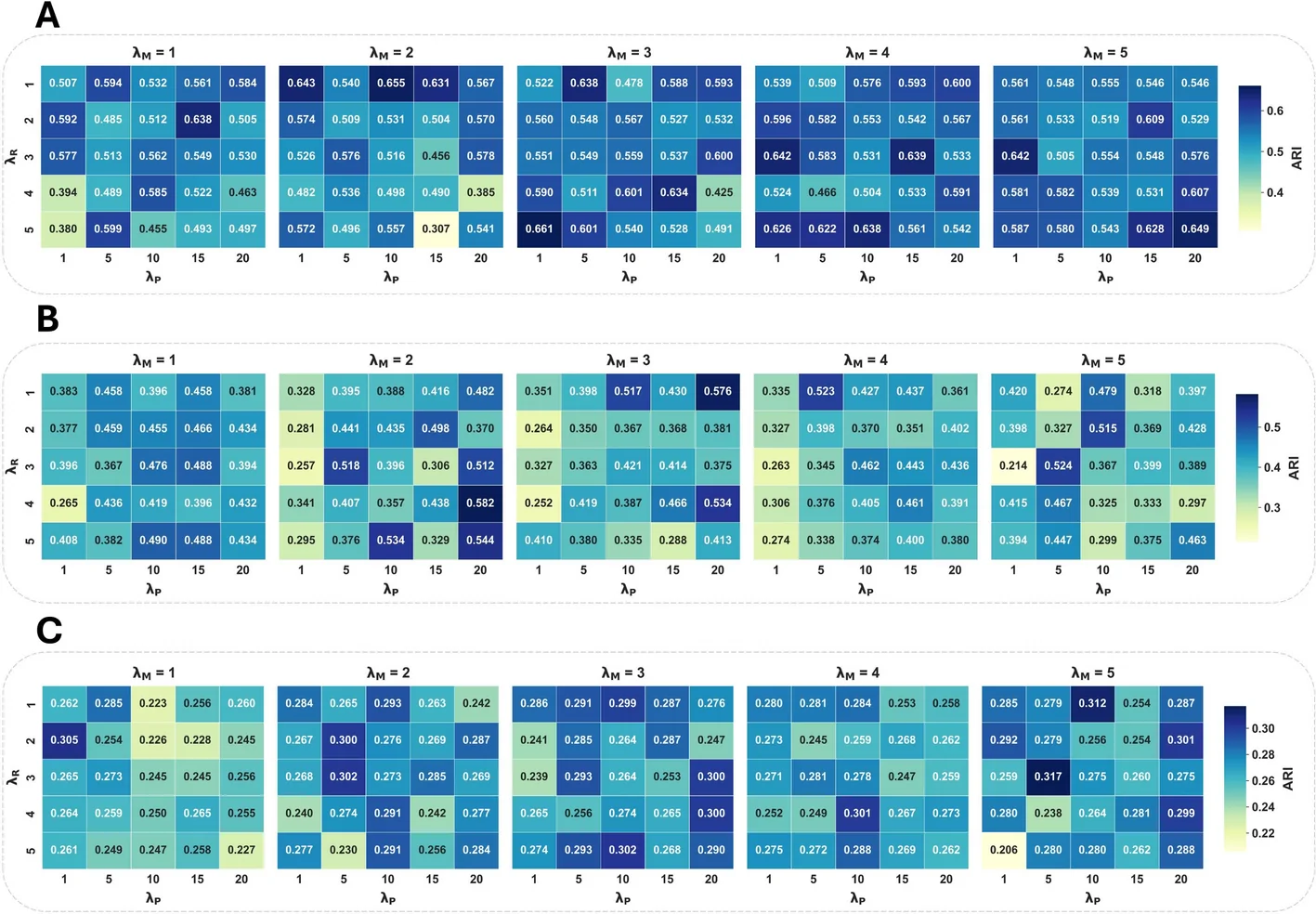
Parameter sensitivity analysis of SINTER3D. **A**-**C** Heatmaps illustrate the impact of Poisson loss weight (${\lambda}_{P}$), intermediate loss weight (${\lambda}_{M}$), and reconstruction loss weight (${\lambda}_{R}$) on model performance (ARI) for the DLPFC, mouse brain, and *Drosophila* embryo datasets, respectively. Color intensity represents the ARI value, with white areas indicating low performance and dark blue areas denoting optimal performance

To comprehensively evaluate the impact of loss function configurations on SINTER3D's performance, we conducted a systematic parameter sensitivity analysis across three datasets of varying complexity. We fixed the intermediate feature loss weight (${\lambda}_{M}$) at different values and performed a grid search to assess how different combinations of the Poisson loss weight (${\lambda}_{P}$) and reconstruction loss weight (${\lambda}_{R}$) affected the model's capability to capture biological structures, as measured by the Adjusted Rand Index (ARI). On the DLPFC dataset (Fig. 8A), the model demonstrated robust performance across a wide parameter range, achieving its optimum (ARI = 0.661) with ${\lambda}_{M}$=3, ${\lambda}_{P}$=1, and ${\lambda}_{R}$=5. Notably, performance declined substantially when ${\lambda}_{M}$=1, indicating that insufficient intermediate feature loss weakens feature learning capability. As ${\lambda}_{M}$ increased, training stability improved significantly—when ${\lambda}_{M}$=5, no parameter configuration resulted in an ARI below 0.53. The mouse brain dataset exhibited more complex parameter dependencies (Fig. 8B), reaching peak performance (ARI = 0.582) with ${\lambda}_{M}$=2, ${\lambda}_{P}$=20, and ${\lambda}_{R}$=4. Compared to the DLPFC dataset, the model showed higher sensitivity to the Poisson loss weight (${\lambda}_{P}$), reflecting the dataset's increased structural complexity and the heightened importance of accurately modeling count distributions. For the *Drosophila* embryo dataset (Fig. 8C), ${\lambda}_{M}$ re-emerged as the dominant factor influencing model performance. Incrementally increasing ${\lambda}_{M}$ progressively enhanced training stability, with optimal performance achieved using ${\lambda}_{M}$=5, ${\lambda}_{P}$=5, and ${\lambda}_{R}$=3.

## Discussion

In this study, we developed SINTER3D to address two key challenges in three-dimensional reconstruction of multi-section spatial transcriptomics data: the loss of information caused by large inter-section gaps and the difficulty of ensuring system-level biological consistency when genes are interpolated independently. By leveraging an implicit neural representation framework, SINTER3D enables joint three-dimensional modeling of multiple genes and achieves robust advantages in interpolation accuracy, spatial-domain identification, and cell-type deconvolution. Conventional three-dimensional reconstruction approaches often rely on discrete section stacking and gene-wise independent interpolation, thereby overlooking the coordinated spatial variation of gene-expression programs and the continuity of tissue structures across inter-section gaps. To overcome these limitations, SINTER3D first constructs a unified three-dimensional spatial coordinate system through spatial registration, mapping multiple two-dimensional sections into a continuous three-dimensional space. It then uses an implicit neural network to learn a continuous mapping from three-dimensional spatial coordinates to latent representations of preprocessed gene-expression profiles, allowing the spatial distribution patterns of multiple genes to be jointly modeled within a unified framework. Meanwhile, a cell-type deconvolution module is introduced to infer cell-type proportions from the latent representations, enabling coordinated reconstruction of gene expression and cellular composition. This paradigm shift from discrete to continuous modeling and from single-gene to multi-gene joint modeling provides a new computational framework for three-dimensional analysis of spatial transcriptomics.

One of the most closely related methods to SINTER3D is STINR, proposed by Luo et al., which likewise introduces implicit neural representation into spatial transcriptomics analysis by learning a continuous mapping from spatial coordinates to gene-expression patterns. STINR demonstrates the feasibility of the INR framework for modeling spatial transcriptomics data, but it mainly focuses on expression analysis within two-dimensional spatial transcriptomics sections. In contrast, SINTER3D extends INR to multi-section three-dimensional reconstruction. By constructing a unified three-dimensional coordinate system, SINTER3D learns continuous mappings from three-dimensional coordinates to multi-gene expression latent representations and cell-type composition, thereby supporting inter-section interpolation, virtual section generation, three-dimensional spatial-domain identification, and reconstruction of cell-type distributions. Thus, SINTER3D complements and extends the application scope of existing INR-based spatial transcriptomics methods.

Across multiple datasets spanning different tissue types, species scales, and technological platforms, SINTER3D demonstrated robust performance. In the adult mouse brain and DLPFC datasets, SINTER3D achieved performance comparable to or better than existing methods in interpolation accuracy, spatial-domain identification, and cell-type deconvolution, while reconstructing three-dimensional gene-expression and cell-type distribution patterns with clear biological relevance. In the developing human heart, *Drosophila* embryo, and HER2-positive breast cancer datasets, SINTER3D further demonstrated its applicability to developmental biology, whole-embryo reconstruction, and tumor microenvironment analysis. Additional analyses showed that SINTER3D remained robust under moderately sparse sampling along the z-axis and could support super-resolution expression prediction within the same section. Moreover, results from the Stereo-seq *Drosophila* embryo and STARmap PLUS datasets indicated that SINTER3D can also be extended to single-cell-resolution spatial transcriptomics data. SINTER3D can also flexibly integrate different spatial registration strategies, such as ICP and CAST, highlighting its modularity and extensibility.

Despite its strong performance across diverse datasets and tasks, SINTER3D has several limitations. Its reconstruction quality may be affected by input section sampling density, registration accuracy, sequencing depth, and tissue structural complexity. When the spacing between sections is excessively large, when the target region lacks sufficient nearby observations, or when tissue morphology changes substantially between adjacent sections, the uncertainty of model predictions may increase. Moreover, the current implementation of SINTER3D primarily relies on spatial coordinates and gene-expression information for continuous modeling. For extremely sparse data or regions with severe local missingness, transcriptomic signals alone may be insufficient to fully recover complex tissue structures. Future work could integrate histological images [48], morphological features, spatial proteomics, or other multimodal information to construct multimodal implicit neural representations, thereby improving reconstruction accuracy in low-coverage regions and complex boundary areas. Another promising direction is to extend SINTER3D into a spatiotemporal four-dimensional modeling framework by integrating spatial transcriptomics data from multiple developmental time points or disease-progression stages. Such a framework could learn a continuous mapping from spatiotemporal coordinates (x,y,z,t) to gene expression and cellular composition, enabling reconstruction of dynamic molecular changes during development, aging, or disease progression.

## Conclusions

SINTER3D provides an implicit neural representation–based framework for continuous three-dimensional reconstruction of multi-section spatial transcriptomics data. By jointly modeling multiple genes as continuous functions of three-dimensional spatial coordinates, SINTER3D overcomes key limitations of discrete section stacking and gene-wise independent interpolation, enabling accurate inter-section interpolation, virtual section generation, spatial-domain identification, and cell-type deconvolution. Its performance across diverse tissues, species, platforms, and resolutions demonstrates its broad applicability for reconstructing biologically meaningful three-dimensional molecular structures. These findings establish SINTER3D as a useful computational framework for spatial transcriptomics and provide a foundation for future studies of three-dimensional tissue organization, developmental processes, disease mechanisms, and precision medicine.

## Methods

### Data preprocessing

SINTER3D selects informative genes based on reference scRNA-seq data. Specifically, for each cell type in the annotated scRNA-seq dataset, SINTER3D applies a *t*-test to identify the top *K* marker genes, with the default setting of *K* = 500. The top marker genes from all cell types are then concatenated to form a comprehensive list of informative genes for downstream analysis in SINTER3D. This gene-selection step enables the model to focus on representative transcriptional signals with cell-type specificity and spatial discriminative power, and to perform joint multi-gene modeling on the filtered set of informative genes. This default setting is designed to balance the retention of cell-type-specific information, model stability, and computational efficiency; the sensitivity analysis under different values of *K* is presented in Additional file 3: Supplementary Section 1.

For the selected *G* genes across *C* cell types, SINTER3D constructs a cell type-specific gene expression profile matrix $V\in {R}^{C\times G}$. First, the gene expression measurements of each cell in the reference scRNA-seq dataset are normalized such that the total gene expression per cell sums to 1. The row vector ${V}_{c}$, representing the average expression signature of cell type *c*, is then computed by averaging the normalized expression values of all cells belonging to that cell type. If the reference scRNA-seq dataset contains multiple batches, SINTER3D calculates a cell type-specific gene expression profile matrix for each batch and subsequently takes their average to obtain the overall matrix *V*.

For three-dimensional coordinate construction, SINTER3D first performs spatial registration of multiple two-dimensional spatial transcriptomics sections to map different sections into a unified coordinate system. By default, SINTER3D uses the iterative closest point (ICP) algorithm [11] for rigid registration. Meanwhile, SINTER3D also supports other registration methods, including PASTE [12] and the non-rigid registration method CAST [43]. Specifically, ICP aligns spatial points by estimating rigid transformations between sections and is suitable for consecutive sections with limited morphological variation and relatively consistent tissue structures. PASTE aligns adjacent sections based on an optimal transport framework, enabling spatial matching while preserving expression similarity across sections. CAST models local deformations between sections through non-rigid spatial registration and is therefore suitable for datasets with substantial morphological variation or local structural differences. Users can select different registration methods in the ‘align_spots’ interface of SINTER3D according to the characteristics of their data. ICP is used as the default method because of its high computational efficiency, whereas CAST is provided as an optional non-rigid registration method for handling local deformation or pronounced morphological differences between sections, where it may further improve spatial registration quality.

### SINTER3D model

SINTER3D adopts a unified framework centered on coordinate-guided latent representation learning, using three-dimensional spatial coordinates as the primary input to learn continuous tissue representations. The framework consists of three modules. To enable coordinate-guided learning, the spatial encoder maps the 3D coordinates ${C}_{\mathrm{c}\mathrm{o}\mathrm{o}\mathrm{r}\mathrm{d}}\in {\mathbb{R}}^{N\times 3}$ to a latent representation $Z\in {\mathbb{R}}^{N\times d}$ that captures spatial continuity, with spatial encoding defined as

1 $$\begin{array}{c}Z={f}_{\theta }\left(\mathrm{N}\mathrm{o}\mathrm{r}\mathrm{m}\mathrm{a}\mathrm{l}\mathrm{i}\mathrm{z}\mathrm{e}\left({C}_{\mathrm{c}\mathrm{o}\mathrm{o}\mathrm{r}\mathrm{d}}\right)\right)\end{array}$$

The deconvolution module predicts the cell-type proportions at each spatial location from the latent representation:

2 $$\begin{array}{c}\pi =softmax\left({g}_{\phi }\left(\mathrm{sin}\left(Z\right)\right)\right)\end{array}$$

where $\pi \in {\mathbb{R}}^{N\times C}$, and C denotes the number of cell types in the reference scRNA-seq data. The spatial location-specific expression offset o is predicted as

3 $$\begin{array}{c}o={h}_{\varphi }\left(\mathrm{sin}\left(\left[Z;{e}_{s}\right]\right)\right)\end{array}$$

where ${e}_{s}$ is a section-specific embedding used to account for systematic variations across sections caused by sequencing depth, technical noise, or differences in sample processing. To ensure information completeness, the expression decoder reconstructs gene-expression profiles from Z, and supervised learning is performed through a feature-reconstruction constraint.

The overall gene-expression prediction follows a Poisson model:

4 $$\begin{array}{c}\mathrm{log}\lambda =\mathrm{log}\left(\pi V+\epsilon \right)+o+{u}_{s}\end{array}$$

and the final count prediction is given by

5 $$\begin{array}{c}\widehat{Y}=L\odot \mathrm{exp}\left(\mathrm{log}\lambda \right)\end{array}$$

where $V\in {\mathbb{R}}^{C\times G}$ is the cell-type-specific expression signature matrix constructed from the reference scRNA-seq data, G denotes the number of informative genes used for modeling, o and ${u}_{s}$ represent the spatial location-specific expression offset and the section-specific global offset parameter, respectively, L is the library-size vector, and ϵ is a small constant added to avoid taking the logarithm of zero.

Compared with conventional methods that directly encode gene expression, this coordinate-driven strategy incorporates spatial continuity as an inductive bias and enables spatial interpolation or extrapolative inference for unobserved tissue sections by learning a continuous three-dimensional spatial mapping.

### Submodel selection strategy

To accommodate dataset heterogeneity, SINTER3D incorporates three specialized submodels tailored to different levels of complexity: The Basic model is optimized for datasets with fewer sections and simple expression patterns. It encodes spatial coordinates using periodic activation functions (sine layers), which naturally capture multi-scale spatial patterns through frequency modulation. The Spatial model handles tissues with high spatial heterogeneity by incorporating multi-scale positional encoding. Coordinates in the *XY*-plane and along the *Z*-axis are processed by independent encoders and then progressively fused to capture both local microstructure and global macrostructure. The Complex model addresses scenarios with significant batch effects and high expression variability via a teacher–student architecture. During training, the teacher branch integrates both coordinate and expression information to provide rich supervisory signals, while the student branch learns to predict using coordinates alone. At inference, only the student branch is deployed, enabling coordinate-driven prediction without gene expression input. A pretrained random forest classifier automatically selects the optimal submodel based on two features extracted from the input data:

Number of sections:

6 $$\begin{array}{c}k=\mid unique\left({\boldsymbol{S}}\right)\mid \end{array}$$

Expression heterogeneity:

7 $$\begin{array}{c}h=\frac{1}{G}{\sum}_{g=1}^{G}Var\left({{\boldsymbol{X}}}_{:,g}\right)\end{array}$$

The classifier was trained on four public spatial transcriptomics benchmark datasets (Additional file 3: Table S2) using pseudo-labels derived from empirical cross-validation performance. Further details are provided in Additional file 3: Supplementary Section 2.

### Multi-task learning objective

SINTER3D jointly optimizes three complementary objectives to ensure the latent representation *Z* simultaneously encodes spatial topology and transcriptional states:

Poisson Negative Log-Likelihood models the statistical properties of count data:

8 $$\begin{array}{c}{\mathcal{L}}_{Poisson}=-\frac{1}{NG}{\sum}_{i,g}\left[{Y}_{ig}\mathrm{log}{\widehat{\lambda }}_{ig}-{\widehat{\lambda }}_{ig}\right]\end{array}$$

This loss enforces that the predicted cell type proportions, combined with the reference signatures, accurately reconstruct the observed gene counts.

Feature Reconstruction Loss constrains the latent space to preserve molecular information:

9 $$\begin{array}{c}{\mathcal{L}}_{Feature}={\lambda}_{M}\parallel X-{f}_{mid}\left(C\right){\parallel}_{2}^{2}+{\lambda}_{R}\parallel X-Decoder\left(Z\right){\parallel}_{2}^{2}\end{array}$$

The first term constrains the intermediate encoder features to retain expression patterns, while the second term enforces end-to-end reconstruction capability. This dual constraint ensures the coordinate-derived latent representation remains informative for underlying gene expression.

The total loss integrates these components:

10 $$\begin{array}{c}{\mathcal{L}}_{total}={\lambda}_{P}{\mathcal{L}}_{Poisson}+{\mathcal{L}}_{Feature}+{\mathcal{L}}_{aux}\end{array}$$

where ${\lambda}_{P}$, ${\lambda}_{M}$, and ${\lambda}_{R}$ denote the weights of the Poisson negative log-likelihood loss, the intermediate feature constraint loss, and the expression reconstruction loss, respectively. ${\mathcal{L}}_{aux}$​ encompasses submodel-specific objectives, such as the Mean Squared Error distillation loss ${L}_{distil}$​ for training the teacher and student branches in the Complex model:

11 $$\begin{array}{c}{L}_{distil}=\parallel {\mathbf{Z}}^{teacher}-{\mathbf{Z}}^{student}{\parallel}_{2}^{2}\end{array}$$

To handle technical variations across tissue sections, SINTER3D incorporates learnable section embeddings (${e}_{s}\in {R}^{{d}_{emb}}$​) and global offset parameters (${u}_{s}\in {R}^{G}$). The learnable section embeddings modulate the deconvolution process by being concatenated with the latent representation prior to cell type proportion prediction. These embeddings capture section-specific cellular composition patterns. The global offset parameters correct for systematic expression shifts in each section, accounting for technical factors such as sequencing depth and platform differences.

For unobserved tissue sections, SINTER3D computes the embedding through spatial distance-weighted interpolation without using gene-expression data from the target section and without retraining the model:

12 $$\begin{array}{c}{e}_{new}={\sum}_{s=1}^{k}{\omega}_{s}\cdot {e}_{s}\end{array}$$

13 $$\begin{array}{c}{\omega}_{s}=\frac{\mathrm{exp}\left(-\frac{{d}_{s}}{\sigma }\right)}{\sum_{{s}^{^{\prime}}}\mathrm{exp}\left(-\frac{{d}_{{s}{\prime}}}{\sigma }\right)}\end{array}$$

Here, ${d}_{s}={\left|{C}_{new}-{C}_{s}\right|}^{2}$ is the squared Euclidean distance between the centroids of the new and measured sections, and $\sigma = std\left({\left\{{d}_{s}\right\}}_{s=1}^{K}\right)+\varepsilon$ adaptively scales the distance metric. This strategy leverages the learned spatial prior to generalize across samples without retraining. When the target location lies between measured sections, this procedure corresponds to spatial interpolation; when the target location lies outside the range of the training sections, it corresponds to spatial extrapolation. This strategy leverages the learned spatial prior to predict gene expression at unobserved section locations without requiring model retraining.

### Network architecture

The three submodels of SINTER3D all employ a hierarchical encoder structure but differ in activation functions and feature processing strategies.

The Basic encoder utilizes four cascaded sine layers followed by two fully connected layers:

14 $$\begin{array}{c}{f}_{enc}\left({\boldsymbol{C}}\right)=Dense\left(SineLayer\left(\cdots SineLayer\left(SineLayer\left(\frac{{\boldsymbol{C}}}{c}\right)\right)\cdots \right)\right)\end{array}$$

The sine layer is defined as $sineLayer\left(x\right)=sin({\omega}_{0}({w}_{x}+b))$, ${\upomega}_{0}$=30. Weights are uniformly initialized in the range *(*$-\frac{\sqrt{6}}{n\cdot {\omega}_{0}},+\frac{\sqrt{6}}{n\cdot {\omega}_{0}}$*)* to ensure an appropriate frequency response.

The Spatial encoder replaces sinusoidal activations with multi-scale positional encoding blocks. Each coordinate dimension is expanded into 2* K* features via:

15 $$\begin{array}{c}PE\left(c,k\right)=\left[\mathit{sin}\left({2}^{0}c\right),\mathit{cos}\left({2}^{0}c\right),\dots ,\mathit{sin}\left({2}^{k-1}c\right),\mathrm{cos}\left({2}^{k-1}c\right)\right]\end{array}$$

We employ *K* = 4 frequency scales. The coordinates are separated into.

${\boldsymbol{C}}=[{{\boldsymbol{C}}}_{xy};{{\boldsymbol{C}}}_{z}]$, and a Fourier-style encoding is applied:

16 $$\begin{array}{c}PE\left(u,\omega \right)=\left[sin\left(u\omega \right),cos\left(u\omega \right)\right]\end{array}$$

with frequencies $\omega ={log}_{2}({2}^{0:\nu -1})$, where *ν* = 12. A dropout rate of 0.1 is added for regularization. The outputs are concatenated and progressively fused through fully connected layers.

The multi-scale encoder is formulated as:

17 $$\begin{array}{c}{{\boldsymbol{F}}}_{xy}=MLP\left(PE\left({{\boldsymbol{C}}}_{xy},\omega \right)\right)\end{array}$$

18 $$\begin{array}{c}{{\boldsymbol{F}}}_{z}=MLP\left(PE\left({{\boldsymbol{C}}}_{z},\omega \right)\right)\end{array}$$

19 $$\begin{array}{c}{{\boldsymbol{F}}}_{coord}=Linear\left(\left[{{\boldsymbol{F}}}_{xy};{{\boldsymbol{F}}}_{z}\right]\right)\end{array}$$

The progressive residual sine stack is defined as:

20 $$\begin{array}{c}{{\boldsymbol{Z}}}^{\left(l+1\right)}=ResSine\left({{\boldsymbol{Z}}}^{\left(l\right)}\right)+{{\boldsymbol{Z}}}^{\left(l\right)}\end{array}$$

where *l* = 4.

The Complex encoder integrates a dual-branch architecture. The spatial coordinate branch shares the same structure as the Basic encoder, while a newly introduced gene expression branch maps the input gene expression $X\epsilon {R}^{N\times G}$ to a 128-dimensional feature space via fully connected layers.

The fusion layer concatenates both branches and applies three fully connected layers with *ReLU* activation for regularization:

21 $$\begin{array}{c}{{\boldsymbol{F}}}_{coord}={f}_{enc}^{\left(c\right)}\left(\frac{{\boldsymbol{C}}}{c}\right)\end{array}$$

22 $$\begin{array}{c}{{\boldsymbol{F}}}_{gene}=Dense\left({\boldsymbol{X}}\right)\end{array}$$

23 $$\begin{array}{c}{{\boldsymbol{Z}}}^{teacher}=Dense\left(\left[{{\boldsymbol{F}}}_{gene};{{\boldsymbol{F}}}_{coord}\right]\right)\end{array}$$

The student prediction is given by ${{\boldsymbol{Z}}}^{student}={f}_{enc}^{(s)}\left(\frac{{\boldsymbol{C}}}{c}\right)$, During training, ${{\boldsymbol{Z}}}^{student}$ is trained to approximate ${{\boldsymbol{Z}}}^{teacher}$, enabling the model to perform predictions using only spatial coordinates at inference by relying solely on ${{\boldsymbol{Z}}}^{student}$.

### Deconvolution module architecture

The deconvolution module contains two parallel predictors: a cell-type proportion predictor and a spatial location-specific offset predictor. To avoid notational ambiguity, we use π to denote cell-type proportions, o to denote spatial location-specific offsets, and ${u}_{s}$ to denote the global section-specific offset parameter.

The cell-type proportion predictor is defined as:

24 $$\begin{array}{c}{H}_{\pi ,1}=BN\left(Z{W}_{\pi ,1}+{q}_{\pi ,1}\right)\end{array}$$

25 $$\begin{array}{c}{H}_{\pi ,2}=BN\left({H}_{\pi ,1}{W}_{\pi ,2}+{q}_{\pi ,2}\right)\end{array}$$

26 $$\begin{array}{c}\pi =Softmax\left({H}_{\pi ,2}\right)\end{array}$$

where $\pi \in {\mathbb{R}}^{N\times C}$ represents the cell-type proportions at each spatial location, and C denotes the number of cell types in the reference scRNA-seq data.

The spatial location-specific offset predictor takes the concatenated representation $\left[{Z}_{m};{e}_{s}\right]$ as input to estimate the local expression offset at each spatial location:

27 $$\begin{array}{c}{H}_{o,1}=BN\left(\left[{Z}_{m};{e}_{s}\right]{W}_{o,1}+{q}_{o,1}\right)\end{array}$$

28 $$\begin{array}{c}{H}_{o,2}=BN\left({H}_{o,1}{W}_{o,2}+{q}_{o,2}\right)\end{array}$$

29 $$\begin{array}{c}o={H}_{o,2}\end{array}$$

where $o\in {\mathbb{R}}^{N\times G}$ denotes the spatial location-specific expression offset, G is the number of informative genes used for modeling, and W and q represent the learnable weight matrices and bias terms of the corresponding fully connected layers, respectively. The section-specific global offset parameter ${u}_{s}\in {\mathbb{R}}^{G}$ is implemented as a directly learnable tensor to correct systematic expression shifts in section s.

### Decoder architecture

The decoder gradually upsamples from the latent space to the gene expression space through four fully connected layers with *ReLU* activation:

30 $$\begin{array}{c}{f}_{recon}\left(Z\right)={BN}_{4}\left(ReLU\left({BN}_{3}\left(ReLU\left({BN}_{2}\left(ReLU\left({BN}_{1}\left(Z{W}_{1}+{b}_{1}\right){W}_{2}+{b}_{2}\right)\right){W}_{3}+{b}_{3}\right)\right)\right)\right)\end{array}$$

No dropout is applied to preserve reconstruction fidelity. The final layer employs linear activation to allow unrestricted prediction of expression values.

### Model training details

SINTER3D employs the Adam optimizer [49] for stochastic optimization during model training. By default, SINTER3D uses a learning rate (lr) of 0.001. An early stopping mechanism is implemented with a patience of 200 epochs and a minimum improvement threshold of σ = 0.0001 to prevent overfitting. All experiments were conducted using a single graphics processing unit. The training time and memory consumption for each spatial transcriptomics dataset are detailed in Additional file 3: Table S4.

### Evaluation of cell type deconvolution

We employed the area under the curve (AUC) score to assess the reliability of cell type deconvolution results. Here, we use the analysis of human dorsolateral prefrontal cortex (DLPFC) data as an example to illustrate the calculation of the AUC score. The spatial study of DLPFC provides layer annotations on the generated DLPFC Visium sections. The single-cell dataset contains 10 excitatory neuronal subtypes across cortical layers. Previous research has investigated the layer specificity of each neuronal subtype in the single-cell reference data [50]. For instance, the neuronal subtype Ext_7_L4_6 was found in the single-cell study to express marker genes for cortical layers 4–6, indicating its localization to these layers. Based on this information, we evaluated the reliability of cell type deconvolution methods by examining whether they could successfully identify the localization of the Ext_7_L4_6 subtype to layers 4–6 through their estimated cell type distributions.

For this purpose, using the manual annotations on the DLPFC Visium sections, we labeled spots in layers 4–6 as "true" and spots in other layers as "false" to establish the ground truth. Subsequently, we performed receiver operating characteristic (ROC) analysis, utilizing the estimated cell type proportion of this subtype (Ext_7_L4_6) for a binary classification task to predict spot labels, thereby generating the ROC curve. The AUC score, calculated as the area under the ROC curve, indicates whether the estimated cell type proportions are distributed across the correct regions. We conducted identical ROC analyses for the other neuronal subtypes. To ensure a fair comparison, the output from each method was normalized such that the sum of all cell type proportions per spot equaled 1 before AUC score evaluation.

### Virtual section generation and interpolation

To interpolate unmeasured regions between real sections, it is first necessary to construct the spatial query coordinates of a virtual section at the target z-position. Because adjacent sections may differ in the number of spots and may exhibit local missing regions and changes in tissue contour, direct global one-to-one matching between the upper and lower sections may introduce unstable spatial distortions. Therefore, we adopted a template-driven strategy combined with lightweight boundary correction to generate the coordinates of virtual sections.

For a target virtual section located at ${z}_{t}$, we first select the real section closest to ${z}_{t}$ as the spatial template:

31 $$\begin{array}{c}{s}_{\mathrm{t}\mathrm{e}\mathrm{m}\mathrm{p}}= arg\underset{s}{\mathrm{min}}\left|{z}_{s}-{z}_{t}\right|\end{array}$$

where ${z}_{s}$ denotes the mean z-coordinate of the s-th real section, and ${s}_{\mathrm{t}\mathrm{e}\mathrm{m}\mathrm{p}}$ denotes the selected template section. Let the two-dimensional coordinates of the i-th spot in the template section be

32 $$\begin{array}{c}X{Y}_{i}^{\left({s}_{\mathrm{t}\mathrm{e}\mathrm{m}\mathrm{p}}\right)}=\left({x}_{i}^{\left({s}_{\mathrm{t}\mathrm{e}\mathrm{m}\mathrm{p}}\right)},{y}_{i}^{\left({s}_{\mathrm{t}\mathrm{e}\mathrm{m}\mathrm{p}}\right)}\right)\end{array}$$

We copy the XY coordinates of all spots in the template section and replace their z-coordinates with the target position ${z}_{t}$, yielding the initial virtual section coordinates:

33 $$\begin{array}{c}{\widetilde{p}}_{i}^{\left(t,0\right)}=\left({x}_{i}^{\left({s}_{\mathrm{t}\mathrm{e}\mathrm{m}\mathrm{p}}\right)},{y}_{i}^{\left({s}_{\mathrm{t}\mathrm{e}\mathrm{m}\mathrm{p}}\right)},{z}_{t}\right)\end{array}$$

This step preserves the real tissue contour and the original spot-array structure, thereby avoiding spatial structural distortions caused by random sampling or unstable cross-section matching.

To further smooth the boundary of the virtual section and enhance contour continuity with adjacent sections, we apply a lightweight correction only to boundary spots in the template section. Specifically, let the two-dimensional geometric center of the template section be

34 $$\begin{array}{c}{c}_{\mathrm{t}\mathrm{e}\mathrm{m}\mathrm{p}}=\frac{1}{{N}_{\mathrm{t}\mathrm{e}\mathrm{m}\mathrm{p}}}\sum_{i}X{Y}_{i}^{\left({s}_{\mathrm{t}\mathrm{e}\mathrm{m}\mathrm{p}}\right)}\end{array}$$

where ${N}_{\mathrm{t}\mathrm{e}\mathrm{m}\mathrm{p}}$ denotes the number of spots in the template section. The radial distance from the i-th spot to the center of the template section is defined as

35 $$\begin{array}{c}{r}_{i}={\parallel X{Y}_{i}^{\left({s}_{\mathrm{t}\mathrm{e}\mathrm{m}\mathrm{p}}\right)}-{c}_{\mathrm{t}\mathrm{e}\mathrm{m}\mathrm{p}}\parallel }_{2}\end{array}$$

and the maximum radial distance is defined as

36 $$\begin{array}{c}{r}_{\mathrm{m}\mathrm{a}\mathrm{x}}=\underset{i}{\mathrm{m}\mathrm{a}\mathrm{x}}{r}_{i}\end{array}$$

Subsequently, the boundary weight is defined according to the normalized radial distance:

37 $$\begin{array}{c}{\lambda}_{i}={\lambda}_{\mathrm{m}\mathrm{a}\mathrm{x}}\cdot clip\left(\frac{\frac{{r}_{i}}{{r}_{\mathrm{m}\mathrm{a}\mathrm{x}}}-\rho }{1-\rho },\hspace{0.17em}0,\hspace{0.17em}1\right)\end{array}$$

where ρ is the boundary-ratio parameter and ${\lambda}_{\mathrm{m}\mathrm{a}\mathrm{x}}$ is the maximum correction strength. This weighting scheme ensures that interior spots remain essentially unchanged, while spots near the tissue contour can undergo slight adjustments. We then use a KDTree [51] to identify corresponding local positions in adjacent reference sections and perform neighborhood smoothing on the resulting displacement field. Let the smoothed boundary displacement be denoted by ${\overline{\Delta } }_{i}$, where ${\overline{\Delta } }_{i}\in {\mathbb{R}}^{2}$ represents the smoothed two-dimensional displacement vector of the i-th spot. The final two-dimensional coordinates are then given by

38 $$\begin{array}{c}X{Y}_{i}^{\mathrm{f}\mathrm{i}\mathrm{n}\mathrm{a}\mathrm{l}}=X{Y}_{i}^{\left({s}_{\mathrm{t}\mathrm{e}\mathrm{m}\mathrm{p}}\right)}+{\lambda}_{i}{\overline{\Delta } }_{i}\end{array}$$

Accordingly, the final three-dimensional query coordinate of the virtual section is

39 $$\begin{array}{c}{\widetilde{p}}_{i}^{t}=\left(X{Y}_{i}^{\mathrm{f}\mathrm{i}\mathrm{n}\mathrm{a}\mathrm{l}},{z}_{t}\right)\end{array}$$

It should be emphasized that this procedure is used only to generate the spatial query coordinates of the virtual section and does not copy gene-expression values from the template section. The expression of each virtual spot is predicted by the trained SINTER3D model based on the new three-dimensional coordinates:

40 $$\begin{array}{c}{\widehat{x}}_{i}^{t}={f}_{\theta }\left({\widetilde{p}}_{i}^{t}\right)\end{array}$$

where ${f}_{\theta }$ denotes the continuous mapping learned by SINTER3D from three-dimensional coordinates to gene expression, and ${\widehat{x}}_{i}^{t}$ denotes the predicted gene-expression vector of the i-th spot in the virtual section. Through this process, SINTER3D can generate virtual section expression maps at unmeasured z-positions while preserving tissue contour constraints and spatial continuity.

To more clearly illustrate the two-dimensional spatial distribution of interpolated spots, we further selected representative virtual sections and visualized the predicted expression of marker genes such as *Tcf7l2*, *Cnp*, and *Mbp*. The results show that the expression patterns in the virtual sections exhibit strong spatial continuity and are consistent with adjacent real sections and known anatomical structures of the mouse brain (see Additional file 3: Fig. S22).

### 3D spatial domain identification

After training SINTER3D on all tissue sections, we obtained latent representations of the data. We then performed unified spatial domain partitioning by clustering the complete set of latent representations using the mclust algorithm [52] in *R*. For mclust, we selected the 'EEE' model, which demonstrates stable performance in spatial transcriptomic data without imposing strong assumptions on cluster shapes. Prior to clustering, we reduced the dimensionality of the latent representations to 20 principal components using PCA to balance information retention and noise reduction.

### Super-resolution reconstruction of 2D sections

To evaluate whether SINTER3D can support within-section super-resolution reconstruction, we conducted additional proof-of-concept experiments on the Visium and ST datasets. In these experiments, new virtual spots were generated between measured spots within the same physical section, and the trained SINTER3D model was used to predict gene expression at these newly added coordinates.

Let the two-dimensional coordinates of the original measured spots in a given section be

41 $$\begin{array}{c}P={\{{p}_{i}=({x}_{i}, { y}_{i})\}}_{i=1}^{N}\end{array}$$

with the corresponding three-dimensional coordinates defined as

42 $$\begin{array}{c}{\widetilde{p}}_{i}=\left({x}_{i},{y}_{i},{z}_{s}\right)\end{array}$$

where ${z}_{s}$ denotes the position of that section along the z-axis in the three-dimensional coordinate system. We first estimated the array center-to-center spacing based on the median nearest-neighbor distance among the original spots:

43 $$\begin{array}{c}d={\mathrm{m}\mathrm{e}\mathrm{d}\mathrm{i}\mathrm{a}\mathrm{n}}_{i}({}_{j\ne i}{}^{min}\Vert {p}_{i}-{{p}_{j}\Vert }_{2})\end{array}$$

For Visium data, where spatial spots are approximately arranged in a hexagonal lattice, point pairs satisfying the following distance criterion were regarded as adjacent:

44 $$\begin{array}{c}\left(1-\epsilon \right)d\le \parallel {p}_{i}-{p}_{j}{\parallel}_{2}\le \left(1+\epsilon \right)d\end{array}$$

A virtual spot was then inserted at the midpoint of each adjacent pair:

45 $$\begin{array}{c}{\widetilde{v}}_{ij}=\left(\frac{{x}_{i}+{x}_{j}}{2},\frac{{y}_{i}+{y}_{j}}{2},{z}_{s}\right)\end{array}$$

For ST data, where spatial spots are approximately distributed on a square grid, we first identified edge-adjacent spot pairs using the same distance criterion and generated virtual spots at the corresponding edge midpoints:

46 $$\begin{array}{c}{\widetilde{v}}_{ij}^{\mathrm{e}\mathrm{d}\mathrm{g}\mathrm{e}}=\left(\frac{{x}_{i}+{x}_{j}}{2},\frac{{y}_{i}+{y}_{j}}{2},{z}_{s}\right)\end{array}$$

In addition, we further identified square grid cells formed by four measured spots and inserted a virtual spot at the center of each square:

47 $$\begin{array}{c}{\widetilde{v}}_{ijkl}^{\mathrm{c}\mathrm{o}\mathrm{r}\mathrm{n}\mathrm{e}\mathrm{r}}=\left(\frac{{x}_{i}+{x}_{j}+{x}_{k}+{x}_{l}}{4},\frac{{y}_{i}+{y}_{j}+{y}_{k}+{y}_{l}}{4},{z}_{s}\right)\end{array}$$

In implementation, square cells were identified based on diagonal point pairs and shared edge-neighbor relationships: if the distance between two points was approximately $\sqrt{2}d$, and the two points shared two common edge-adjacent neighbors, then the corresponding four-point combination was considered to form a square cell. All virtual spots were assigned the same ${z}_{s}$ coordinate as their corresponding physical section, thereby ensuring that they represent unmeasured spatial locations within the same section.

After spatial densification, the three-dimensional coordinates of the virtual spots were input into the trained SINTER3D model for expression prediction:

48 $$\begin{array}{c}{\widehat{x}}_{m}={f}_{\theta }\left({\widetilde{v}}_{m}\right)\end{array}$$

where ${f}_{\theta }$ denotes the trained coordinate-to-expression mapping function learned by SINTER3D, ${\widetilde{v}}_{m}$ represents the three-dimensional coordinate of the m-th virtual spot, and ${\widehat{x}}_{m}$ denotes its predicted gene-expression vector. Through this process, SINTER3D can generate high-resolution spatial gene-expression maps within the same physical section.

### Baseline methods

All baseline methods were run using the default parameters recommended in the original papers or official code repositories. The code sources, parameter settings, and implementation details for each baseline method are provided in Additional file 3: Table S5.

## Supplementary Information

Additional file 1: This file includes Figs. S1-Fig S8.

Additional file 2: This file includes Figs. S9-Fig S16.

Additional file 3: This file includes Figs. S17-S26, Tables S1-S7, and Supplementary Sections 1-3.

## Data Availability

All data used in this study are publicly available from online resources: the human dorsolateral prefrontal cortex dataset generated by the Visium platform [6, 53] (http://spatial.libd.org/spatialLIBD/); the human dorsolateral prefrontal cortex dataset sequenced by the 10 × Genomics Chromium platform (GSE144136) [54]; the mouse whole-brain dataset generated by the ST platform [5, 55] (GSE147747); the mouse brain dataset sequenced by the 10 × Genomics Chromium platform [25, 56] (E-MTAB-11115) (https://www.ebi.ac.uk/biostudies/arrayexpress/studies/E-MTAB-11115); the human embryonic heart dataset generated by the ST platform [7, 57] (https://data.mendeley.com/datasets/dgnysc3zn5/1); the human embryonic heart dataset sequenced by the 10 × Genomics Chromium platform [7, 58] (https://data.mendeley.com/datasets/mbvhhf8m62/2); the HER2-positive breast tumor dataset generated by the ST platform [59, 60] (10.5281/zenodo.4751624); the HER2-positive breast tumor dataset sequenced by the 10 × Genomics Chromium platform [61, 62] (GSE176078); the Drosophila embryo dataset generated by Stereo-seq [8, 63] (https://db.cngb.org/stomics/datasets/STDS0000060); the Drosophila embryo dataset [39, 64] sequenced by sci-RNA-seq (GSE190149). The single-cell-resolution mouse brain spatial transcriptomics dataset generated using the STARmap PLUS platform is available through the Single Cell Portal (SCP1375; https://singlecell.broadinstitute.org/single_cell/study/SCP1375) [65]. The source code of SINTER3D used in this manuscript has been deposited in GitHub (https://github.com/hezi-li/SINTER3D) under an Apache License 2.0 [66] and is also available in a DOI-assigning repository, Zenodo, at https://doi.org/10.5281/zenodo.20550649 [67] under the same Apache license.
