# Supplementary material for: SINTER3D: continuous 3D reconstruction of spatial transcriptomics via implicit neural representations
Source: Genome Biol. 2026 Jun 17;27:274. doi: 10.1186/s13059-026-04160-5 (PMC13508360; doi:10.1186/s13059-026-04160-5)
Supplement: Supplementary file 1 [file 13059_2026_4160_MOESM1_ESM.pdf]

Supplementary Figures

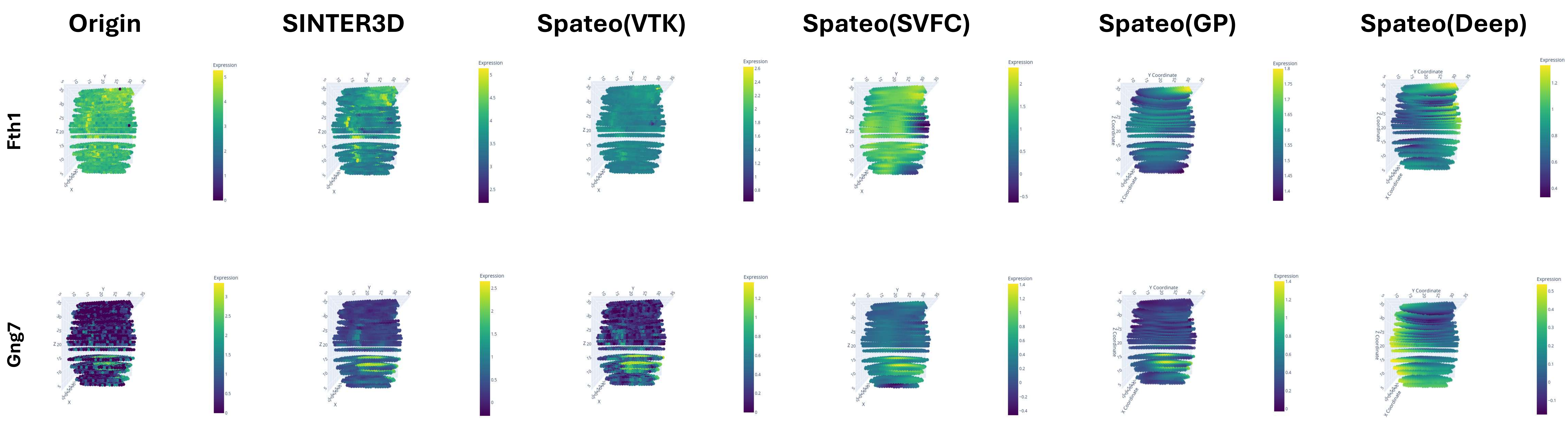

Fig S1. Comparison of original gene expression and results obtained after interpolation using various interpolation methods on a mouse brain test dataset

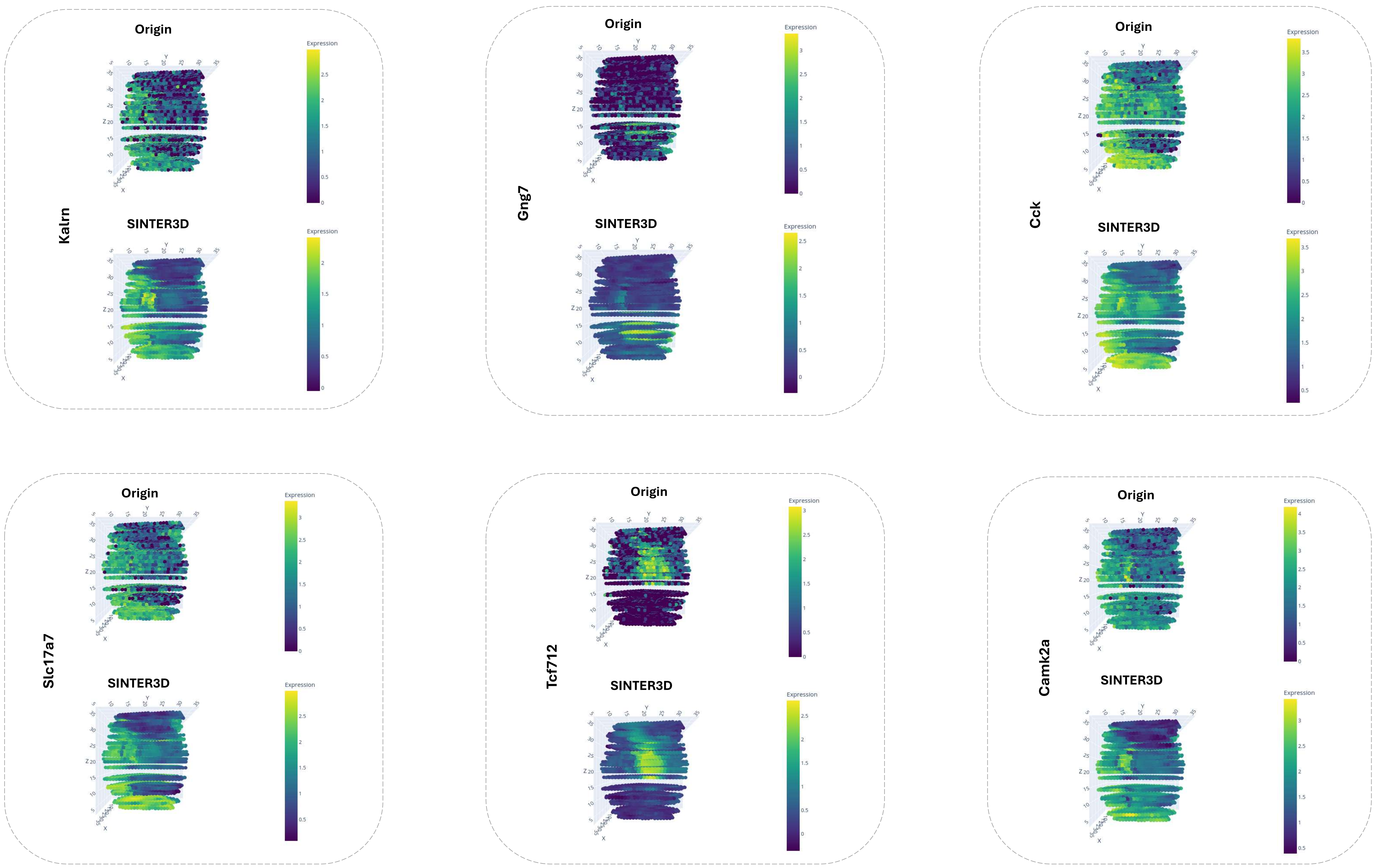

Fig S2. A 3D comparison between the imputed data generated by SINTER3D and the original gene expression data from the mouse whole-brain test set.

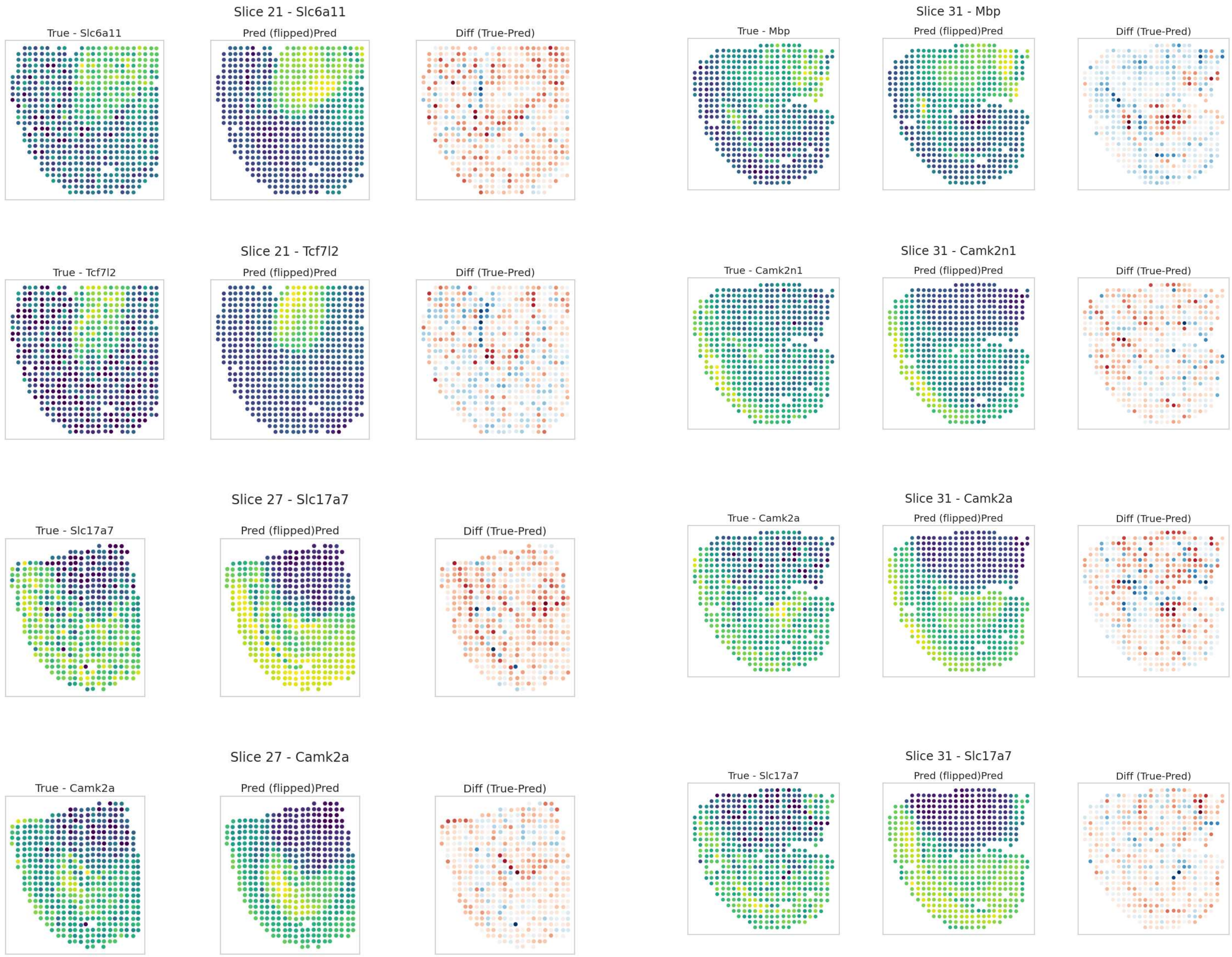

Fig S3. A 2D comparison of the imputed data generated by SINTER3D and the original gene expression data from the mouse whole-brain test set.

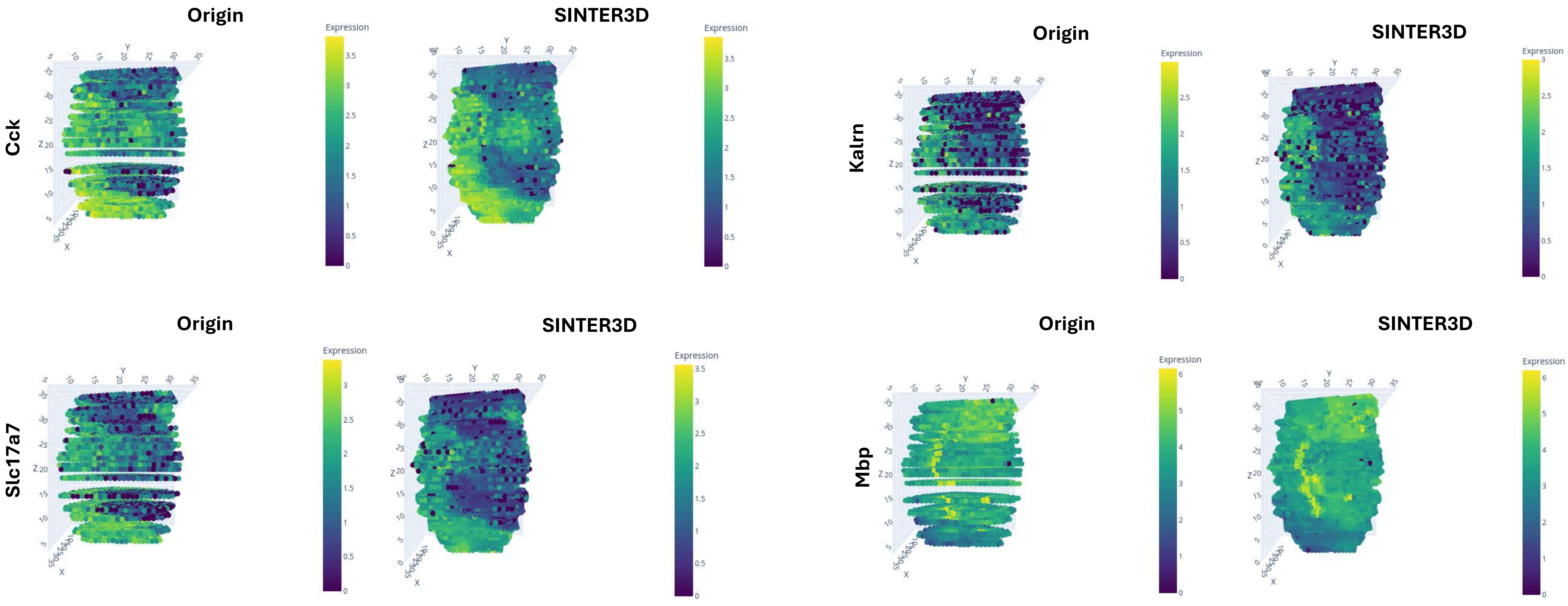

Fig S4. Comparison of 3D gene expression visualizations for the mouse whole-brain dataset before and after imputation by SINTER3D.

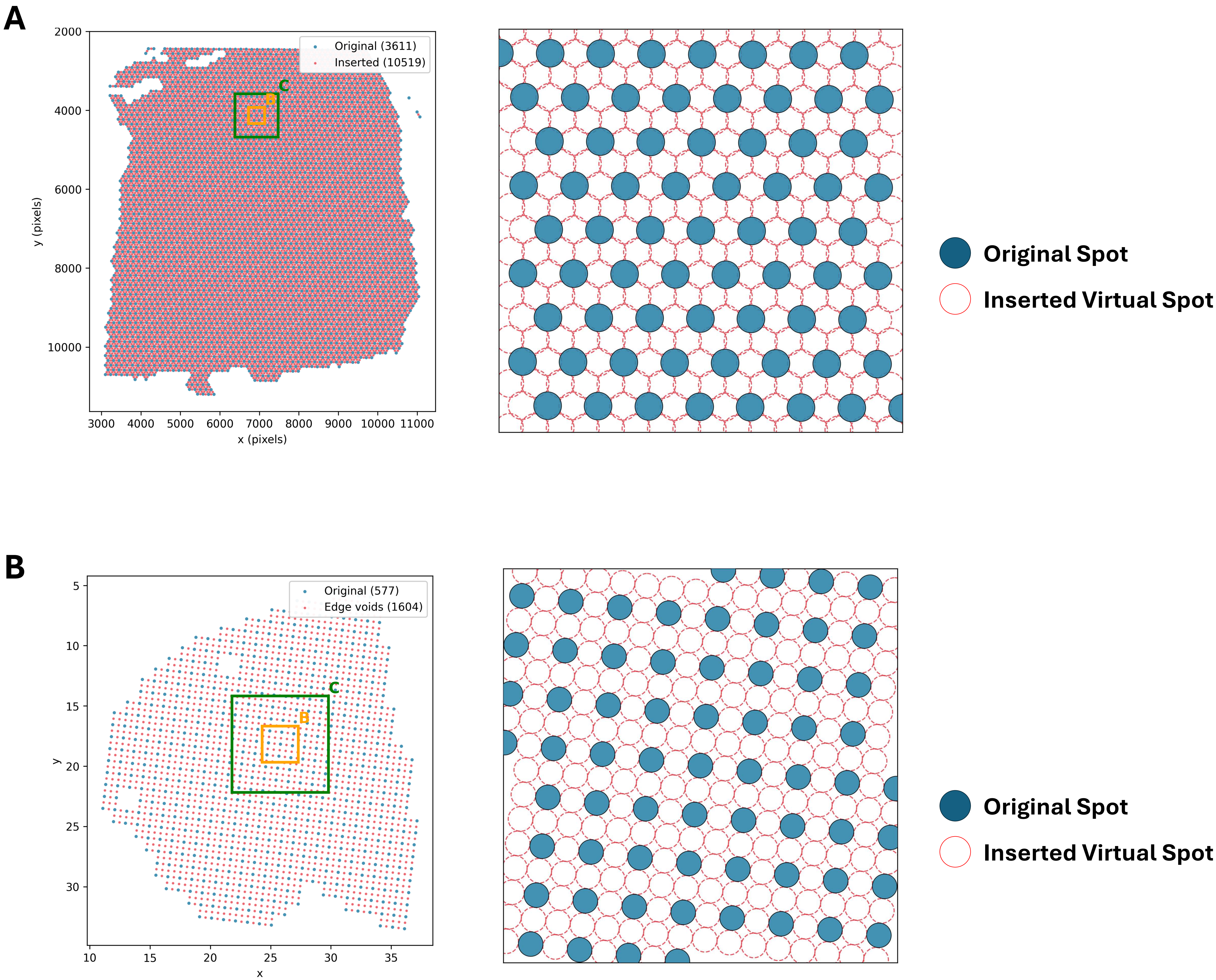

Fig S5. Intra-slice super-resolution reconstruction by SINTER3D.

A, Geometry-aware spot densification strategy for Visium sections. Virtual spots were inserted at the midpoints between neighboring measured spots according to the hexagonal array layout.

B, Geometry-aware spot densification strategy for ST sections. Virtual spots were inserted at both edge midpoints and square centers according to the ST square-grid layout.

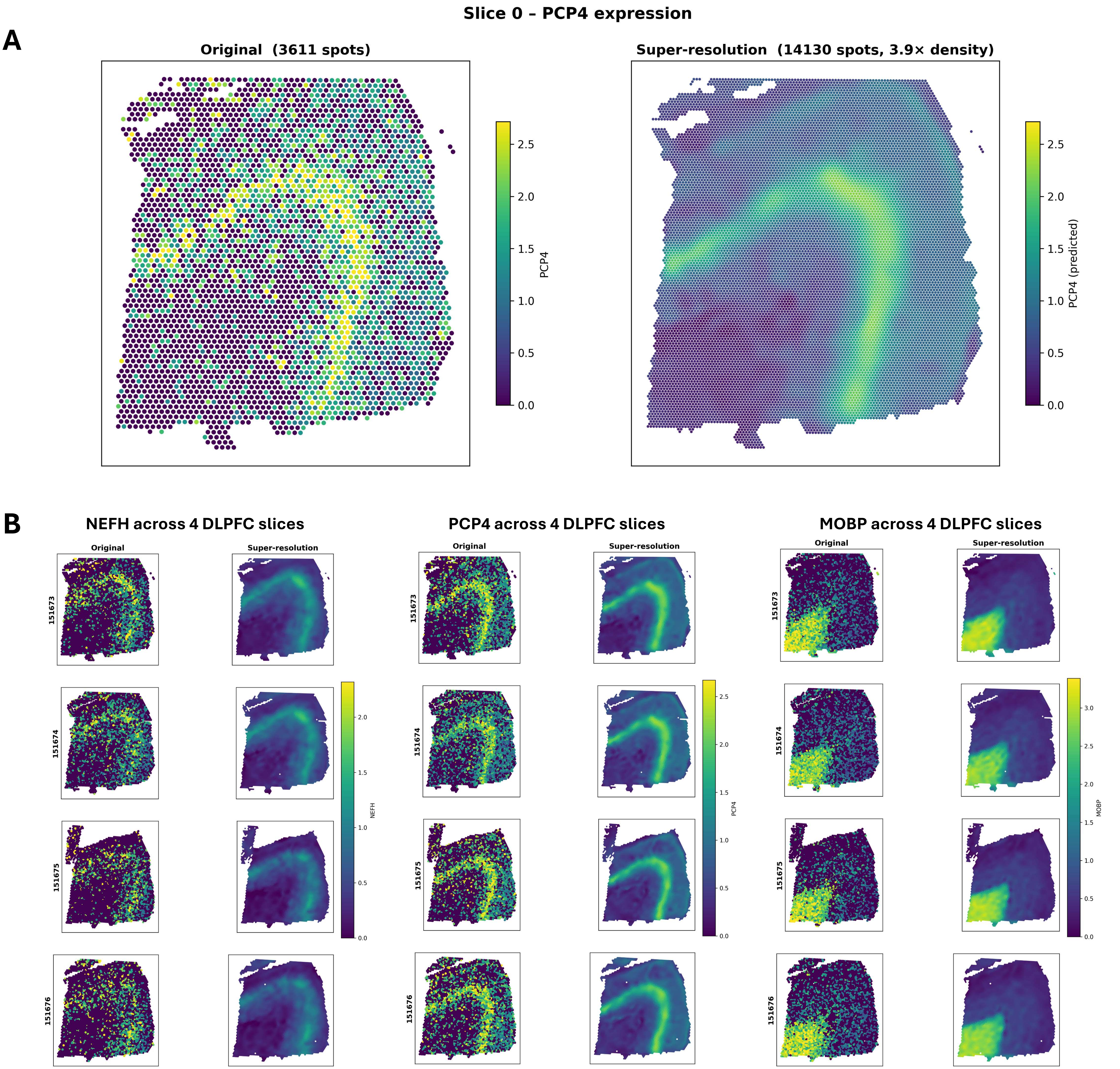

Fig S6. Intra-slice super-resolution reconstruction of Visium data via SINTER3D.

A, Representative super-resolution reconstruction of marker-gene expression in a DLPFC Visium section. Original measured spots and SINTER3D-predicted high-resolution spots are shown side by side using the same color scale.

B, Super-resolution reconstruction of representative marker genes across multiple DLPFC Visium sections.

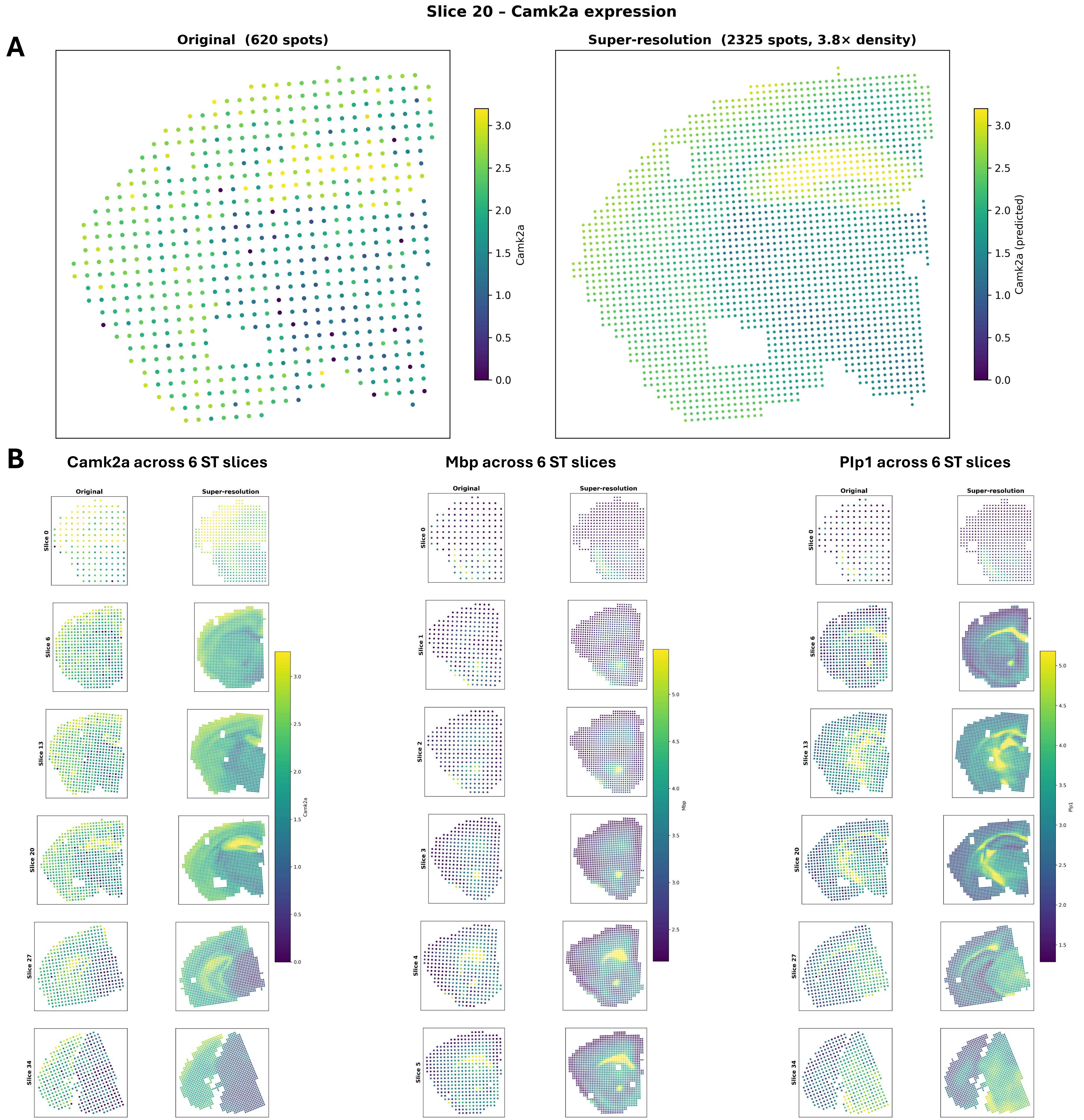

Fig S7. Intra-slice super-resolution reconstruction of ST data via SINTER3D.

A, Representative super-resolution reconstruction of marker-gene expression in an adult mouse brain ST section.

B, Super-resolution reconstruction of representative marker genes across multiple adult mouse brain ST sections. These results show that SINTER3D can generate biologically plausible expression predictions at newly inserted intra-slice coordinates, supporting its use for within-section super-resolution.

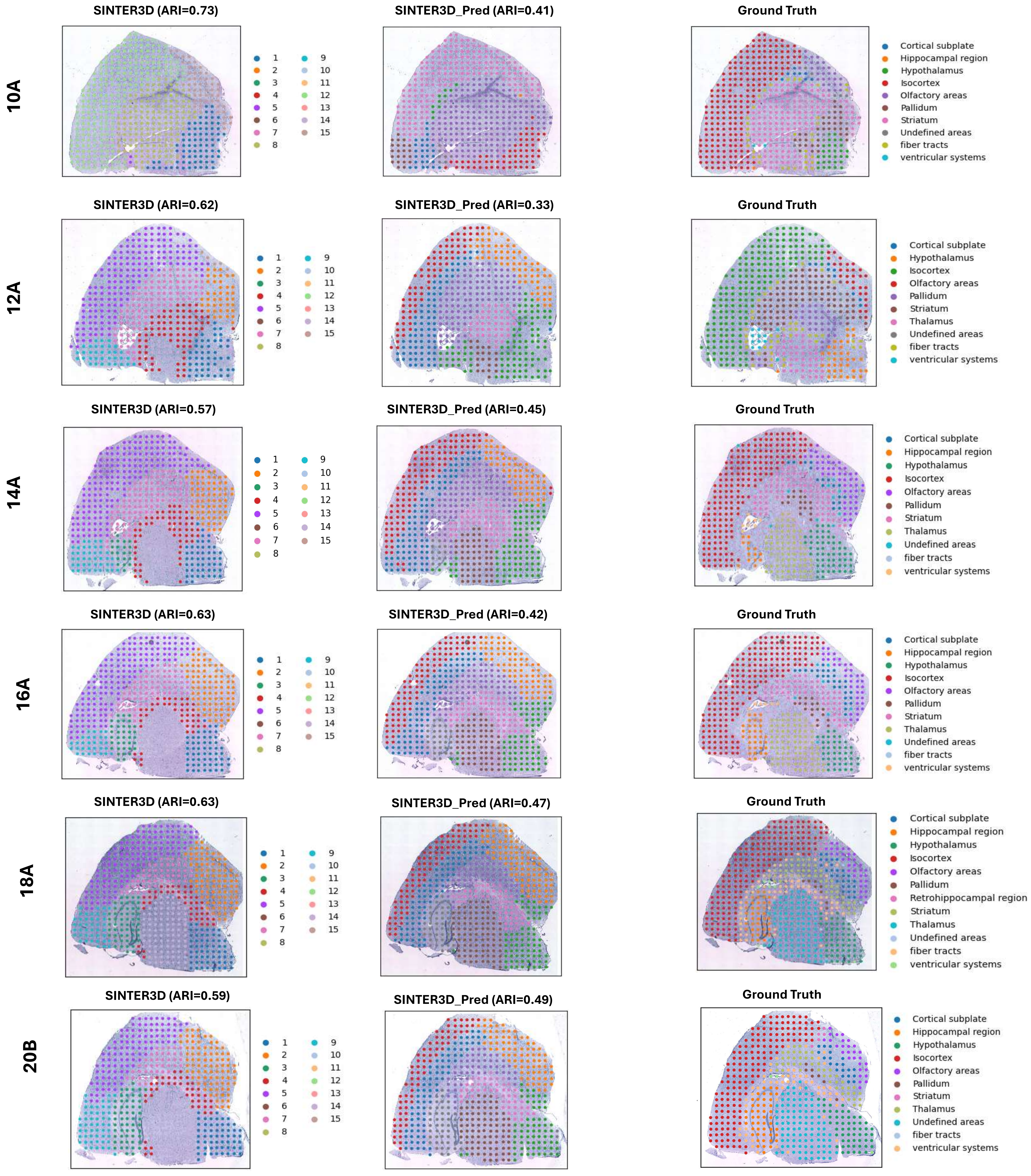

Fig S8. Comparison of spatial domain identification results based on ground truth data versus SINTER3D-imputed data on the test set of the mouse whole-brain dataset.
