# Supplementary material for: SINTER3D: continuous 3D reconstruction of spatial transcriptomics via implicit neural representations
Source: Genome Biol. 2026 Jun 17;27:274. doi: 10.1186/s13059-026-04160-5 (PMC13508360; doi:10.1186/s13059-026-04160-5)
Supplement: Supplementary file 2 [file 13059_2026_4160_MOESM2_ESM.pdf]

# Supplementary Figures

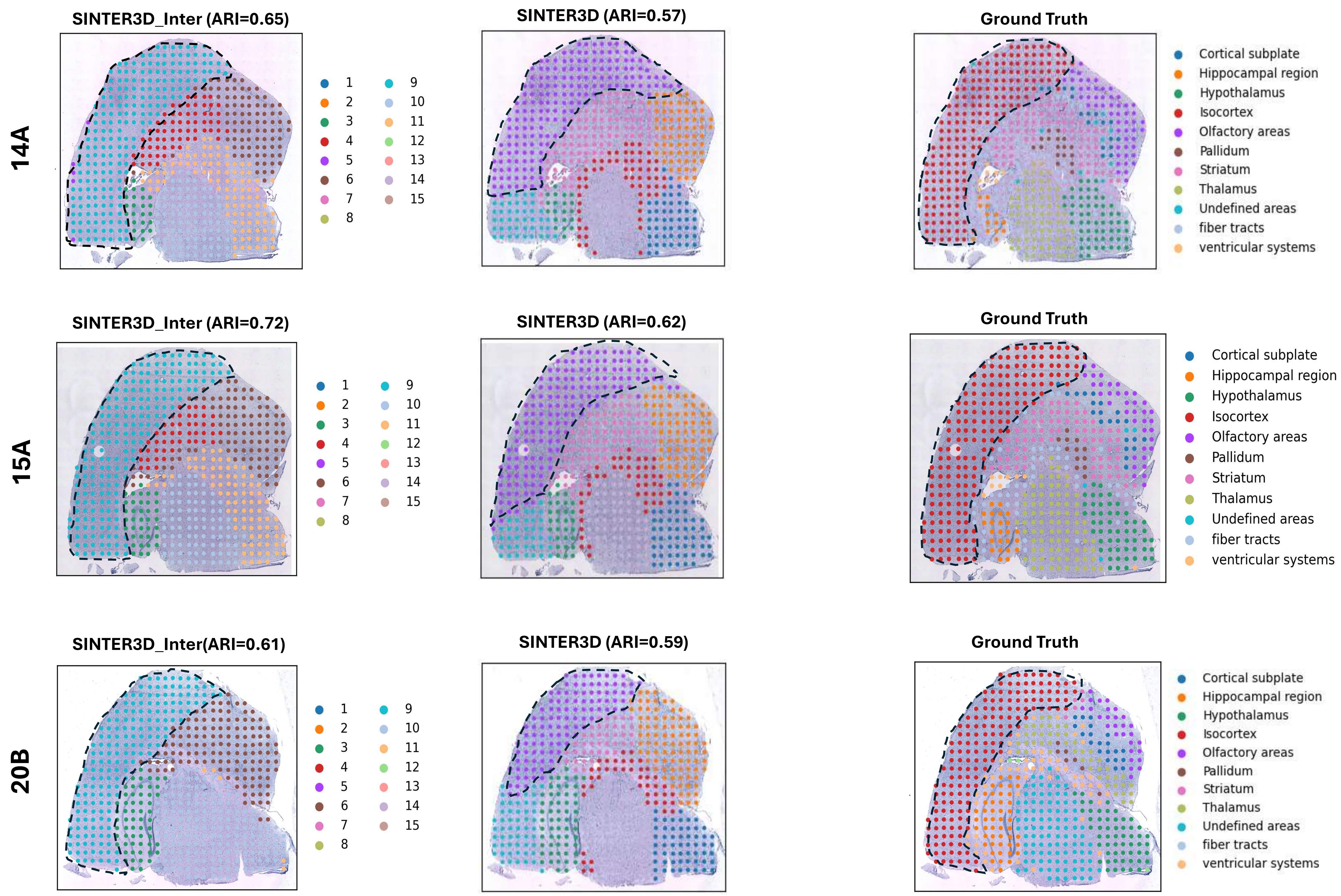


**Fig S9.** Comparison of spatial domain identification results between the original and SINTER3D-imputed mouse whole-brain datasets.

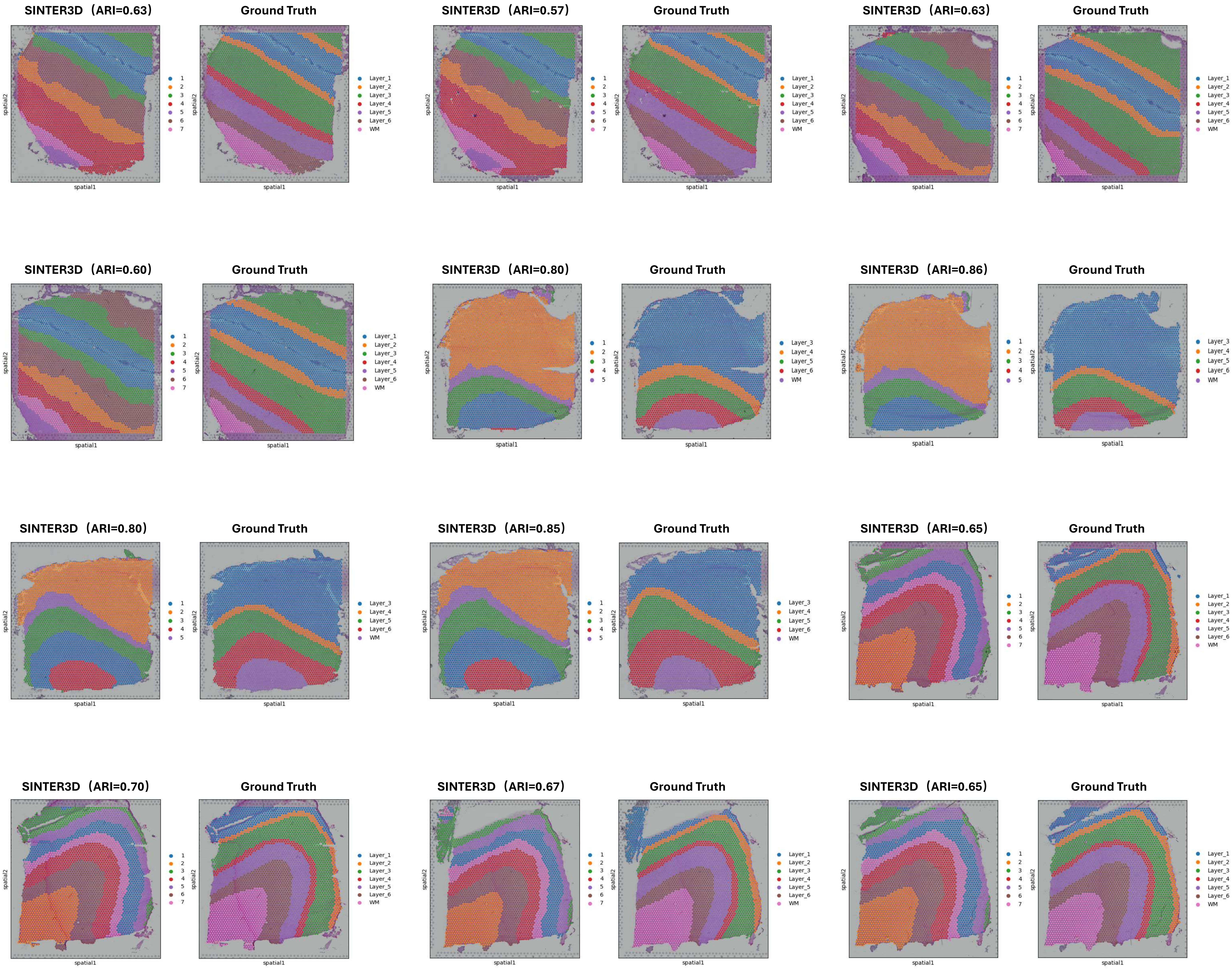


**Fig S10.** Spatial domain identification results of SINTER3D on 12 sections of the DLPFC dataset.

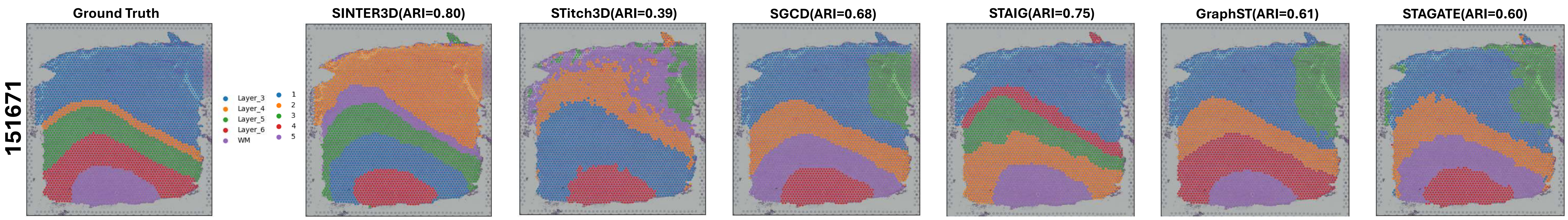


**Fig S11.** Comparison of spatial domain segmentation results by different methods for section #151671 from the DLPFC dataset.

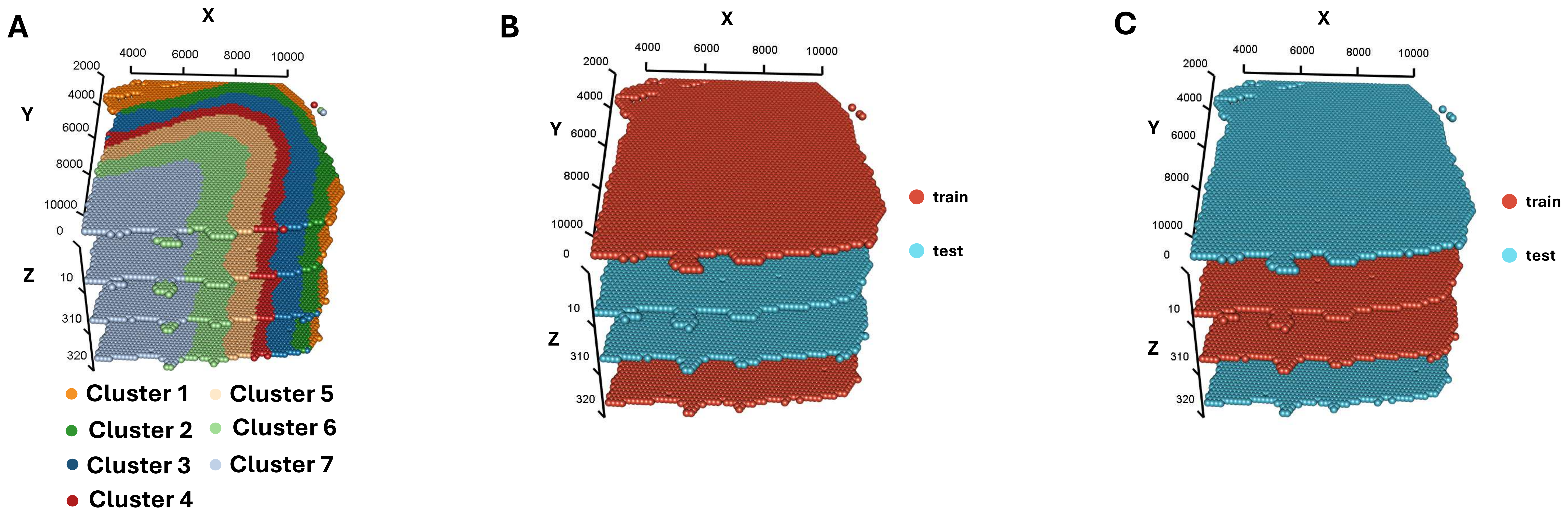


**Fig S12.** Configuration of training and test sets for interpolation and extrapolation experiments on the DLPFC dataset. (A) Ground truth spatial domain labels for sections #151673-76 of the DLPFC. (B) Division of training and test data for the interpolation experiment. (C) Division of training and test data for the extrapolation experiment.

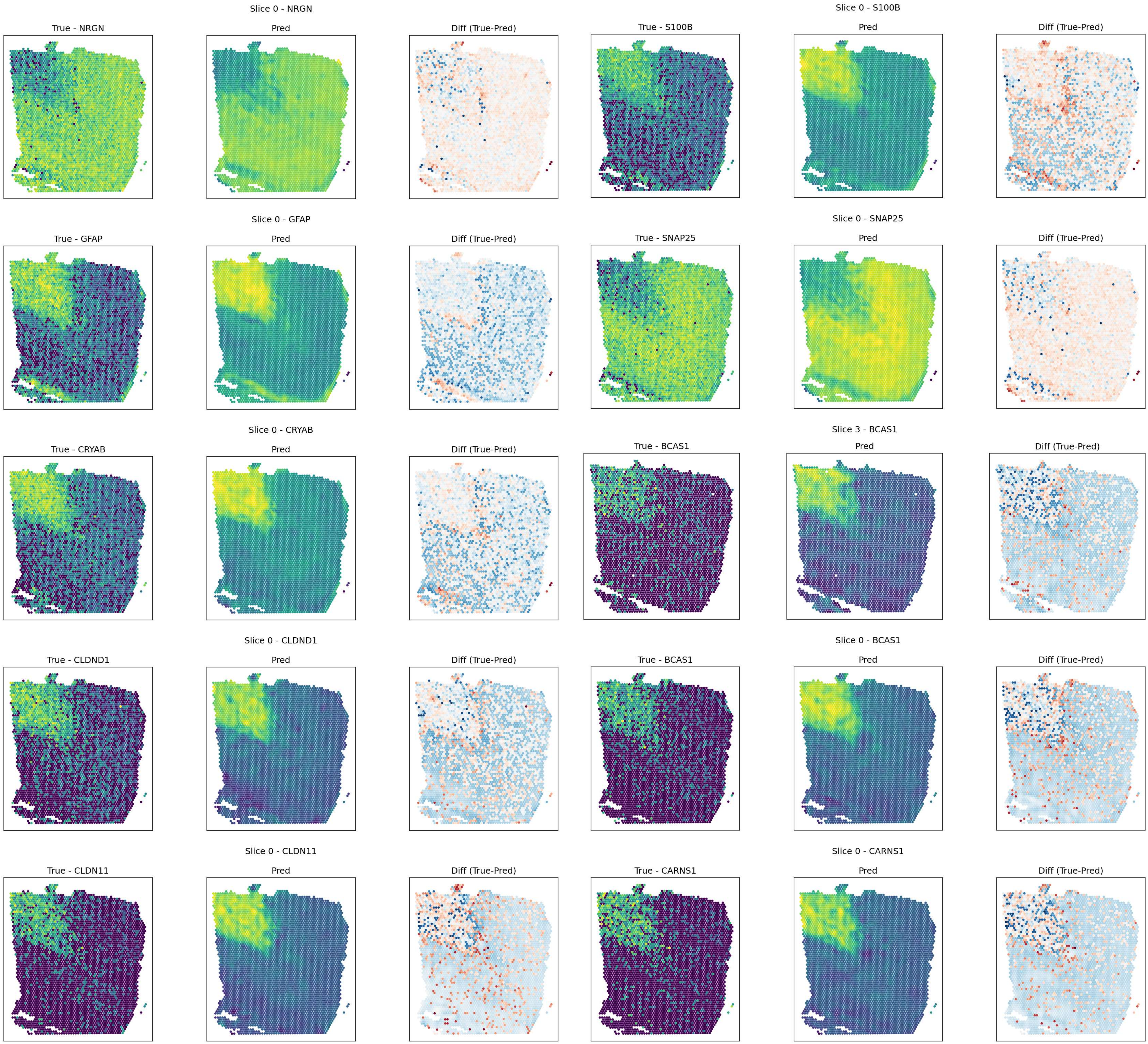


**Fig S13.** Comparison between the gene expression imputation results and the original results for section #151673 during the spatial extrapolation experiment on the DLPFC dataset.

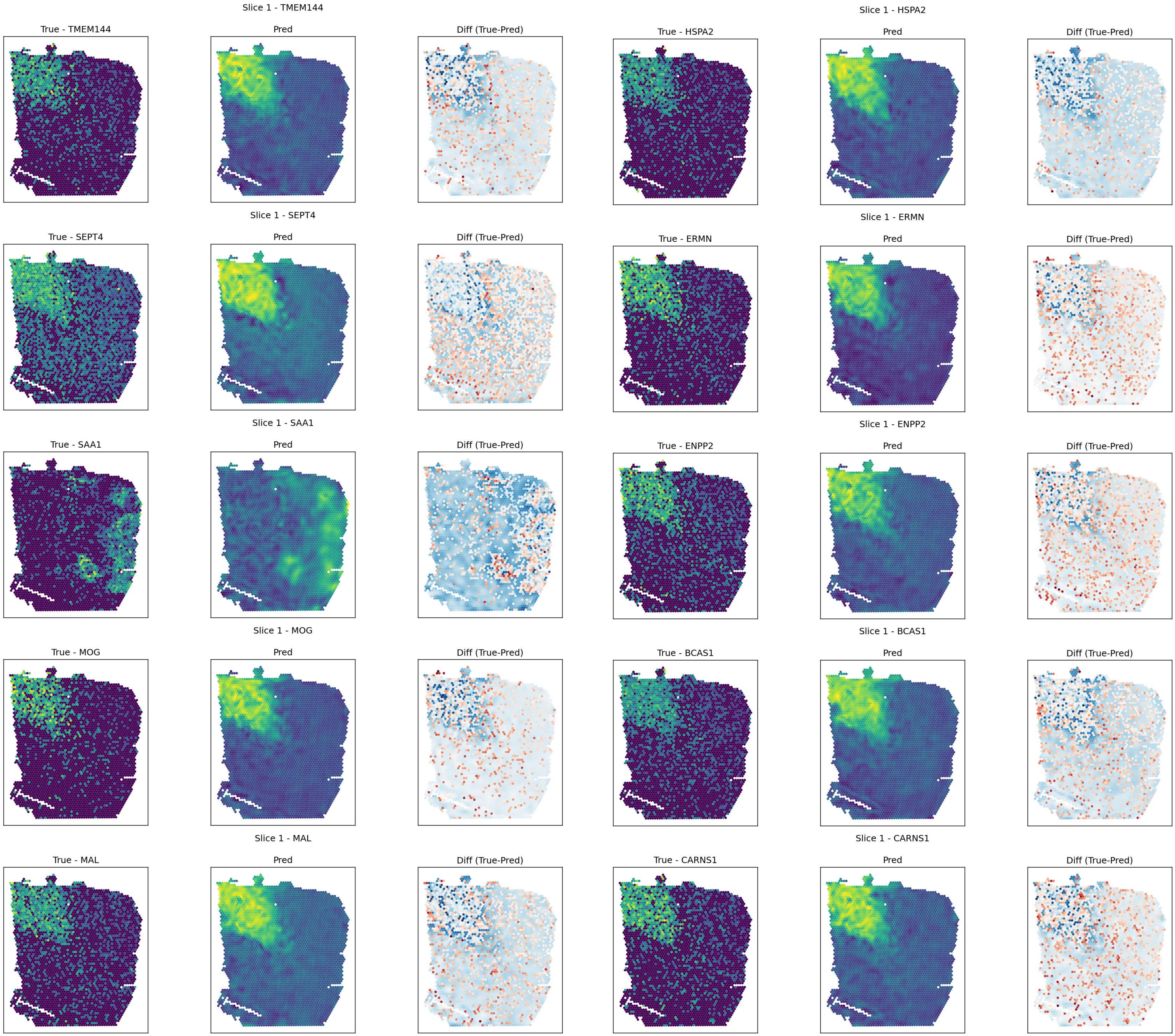


**Fig S14.** Comparison between the imputed and original gene expression results for section #151674 from the spatial interpolation experiment on the DLPFC dataset.

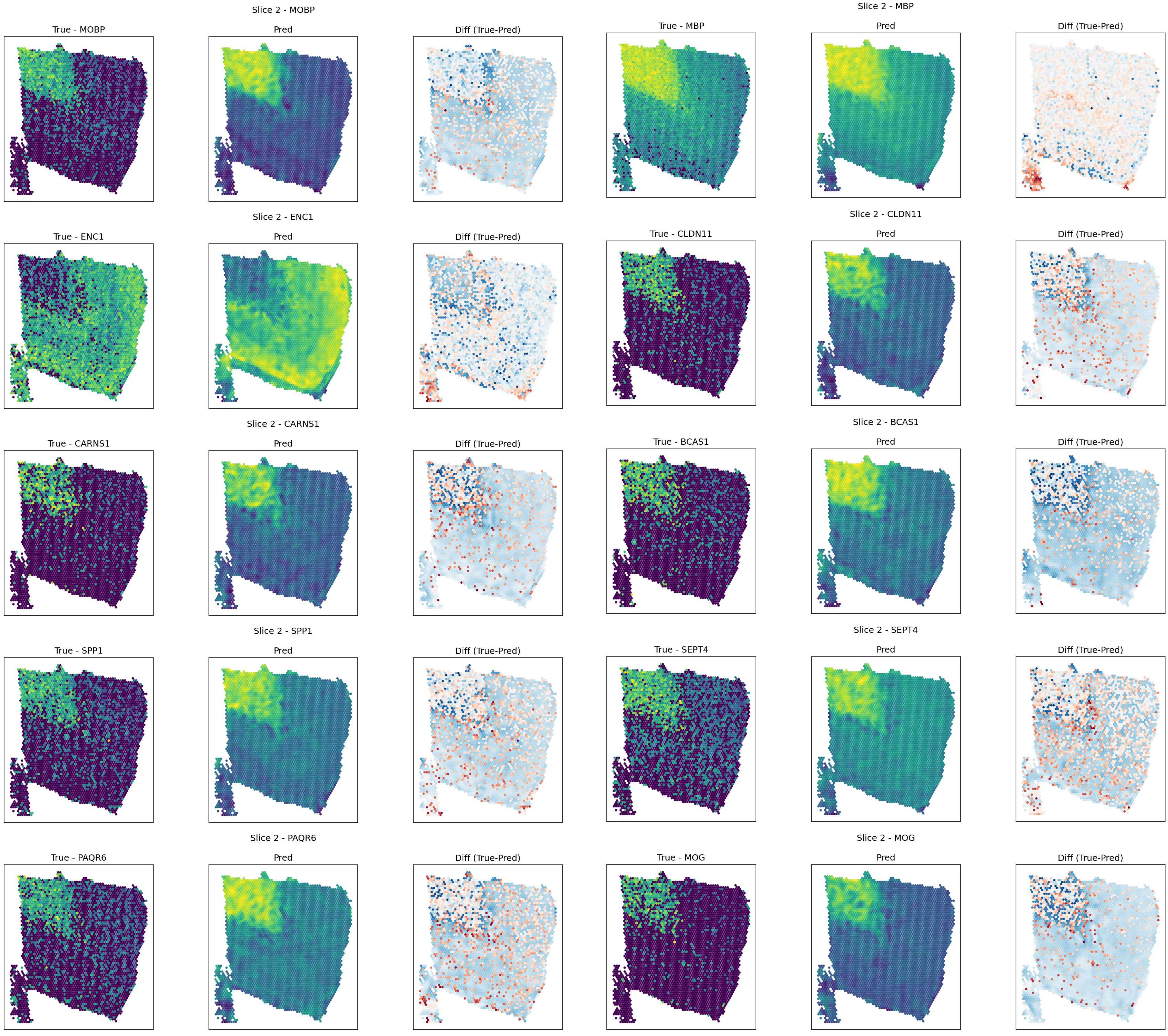


**Fig S15.** Comparison between the gene expression imputation results and the original results for section #151675 during the spatial interpolation experiment on the DLPFC dataset.

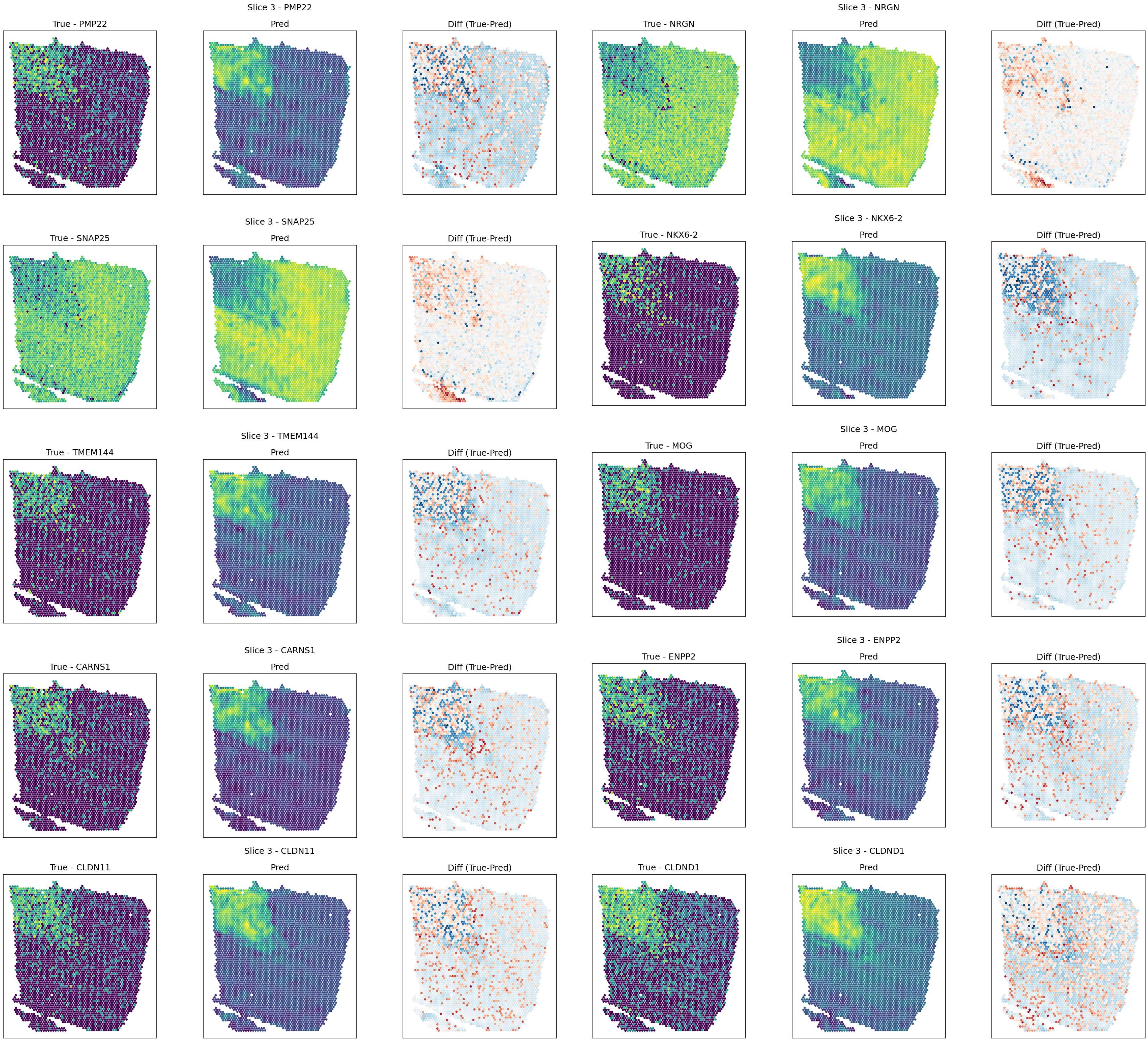


**Fig S16.** Comparison between the gene expression imputation results and the original results for section #151676 during the spatial extrapolation experiment on the DLPFC dataset.
