# Supplementary material for: SINTER3D: continuous 3D reconstruction of spatial transcriptomics via implicit neural representations
Source: Genome Biol. 2026 Jun 17;27:274. doi: 10.1186/s13059-026-04160-5 (PMC13508360; doi:10.1186/s13059-026-04160-5)
Supplement: Supplementary file 3 [file 13059_2026_4160_MOESM3_ESM.pdf]

Supplementary Figures

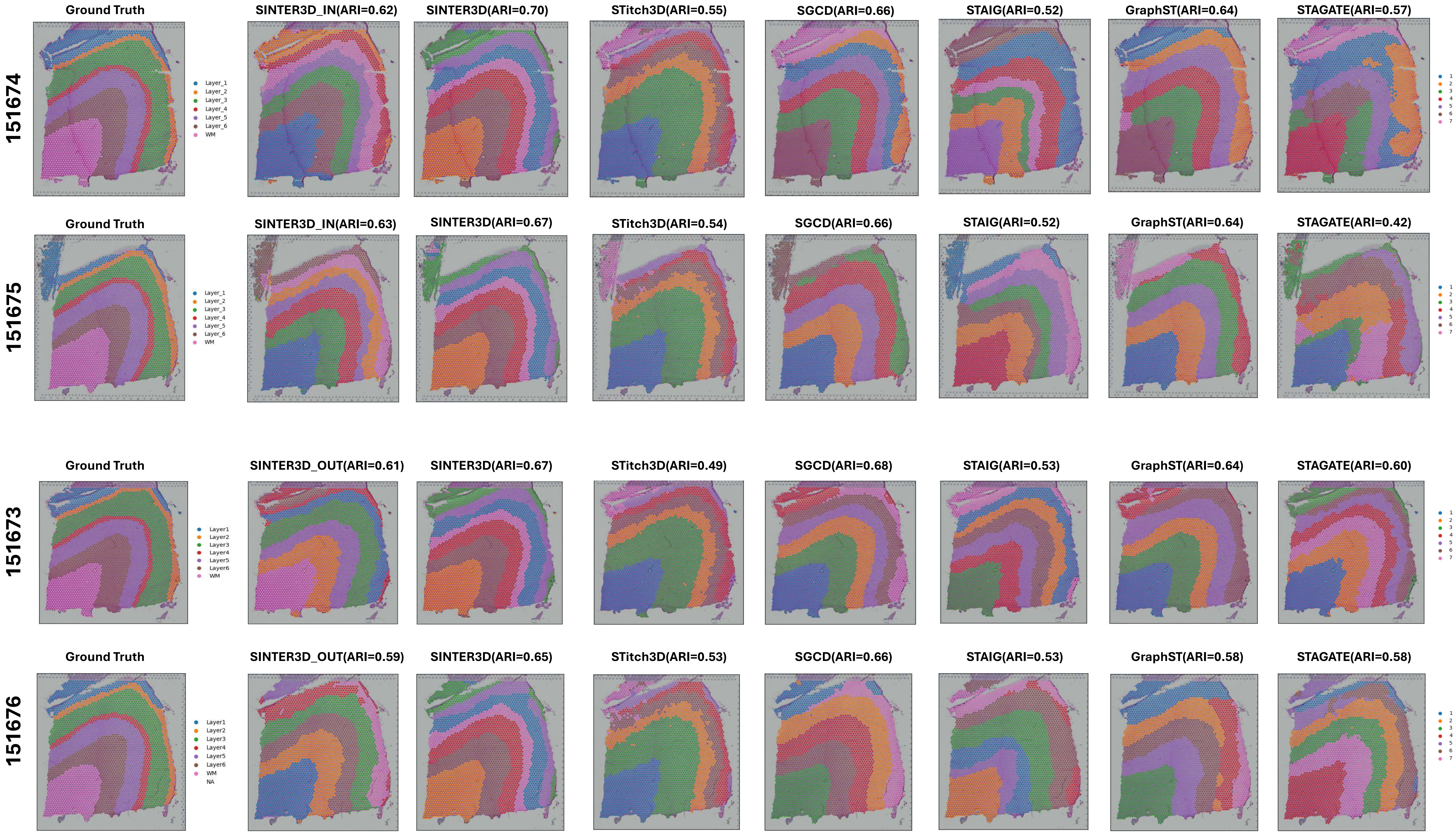

Fig S17. Configuration of training and test sets for interpolation and extrapolation experiments on the DLPFC dataset. (A) Ground truth spatial domain labels for sections #151673-76 of the DLPFC. (B) Division of training and test data for the interpolation experiment. (C) Division of training and test data for the extrapolation experiment.

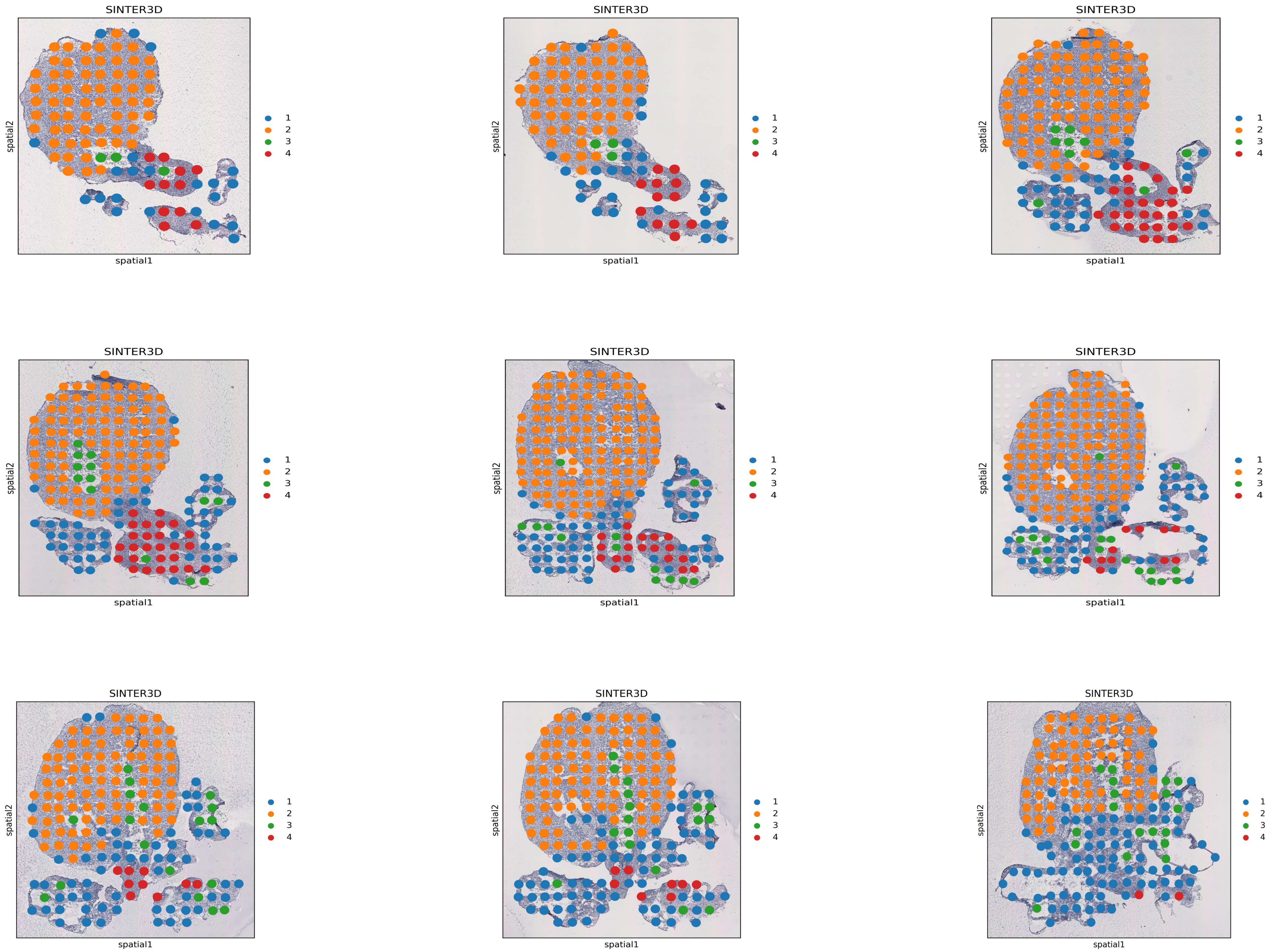

Fig S18. Spatial domain identification results of SINTER3D on 9 sections of the human heart dataset at 6.5 post-conception weeks (6.5-PCW).

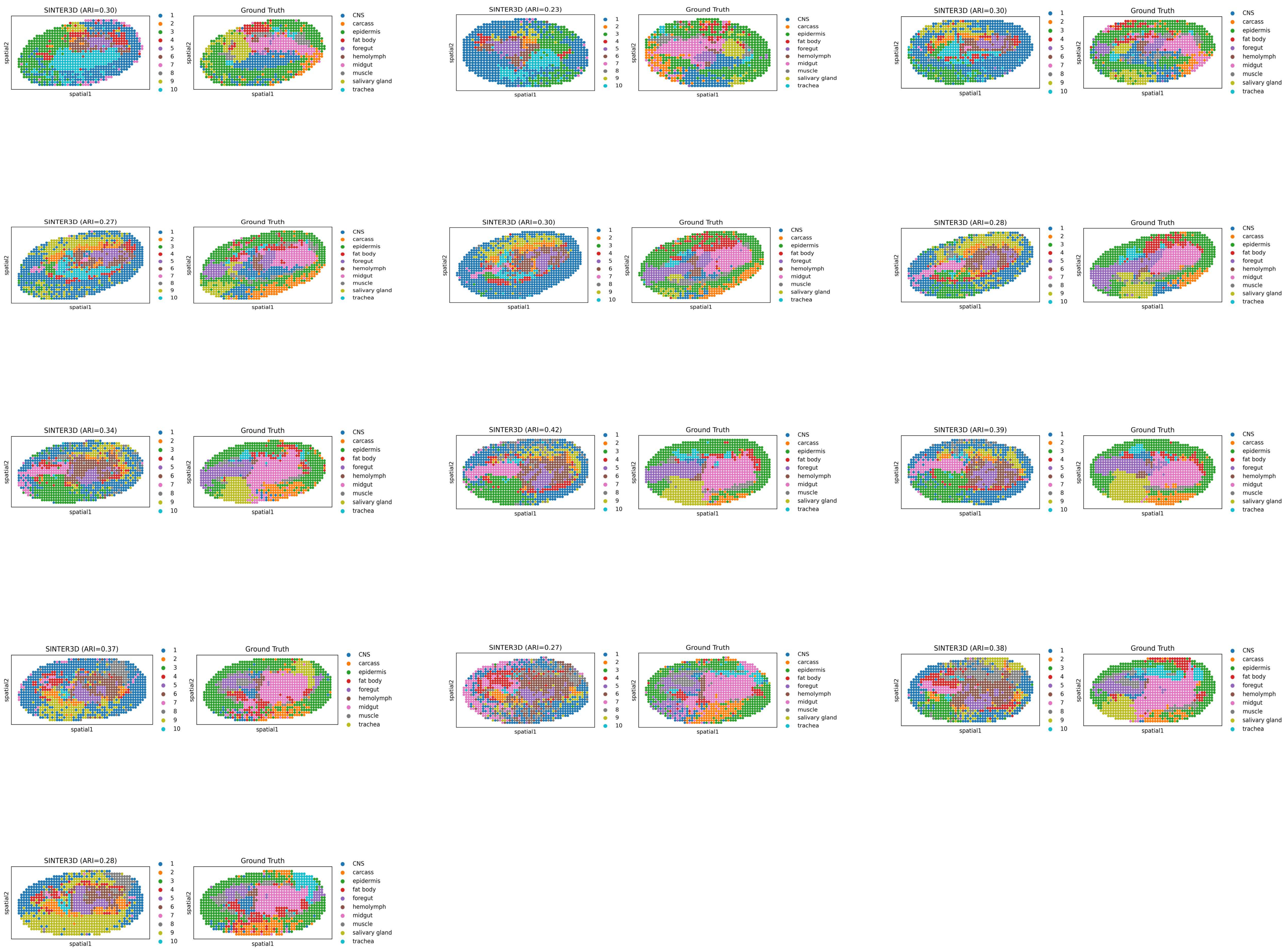

Fig S19. SINTER3D's spatial domain identification results for 13 sections of the Drosophila embryo dataset.

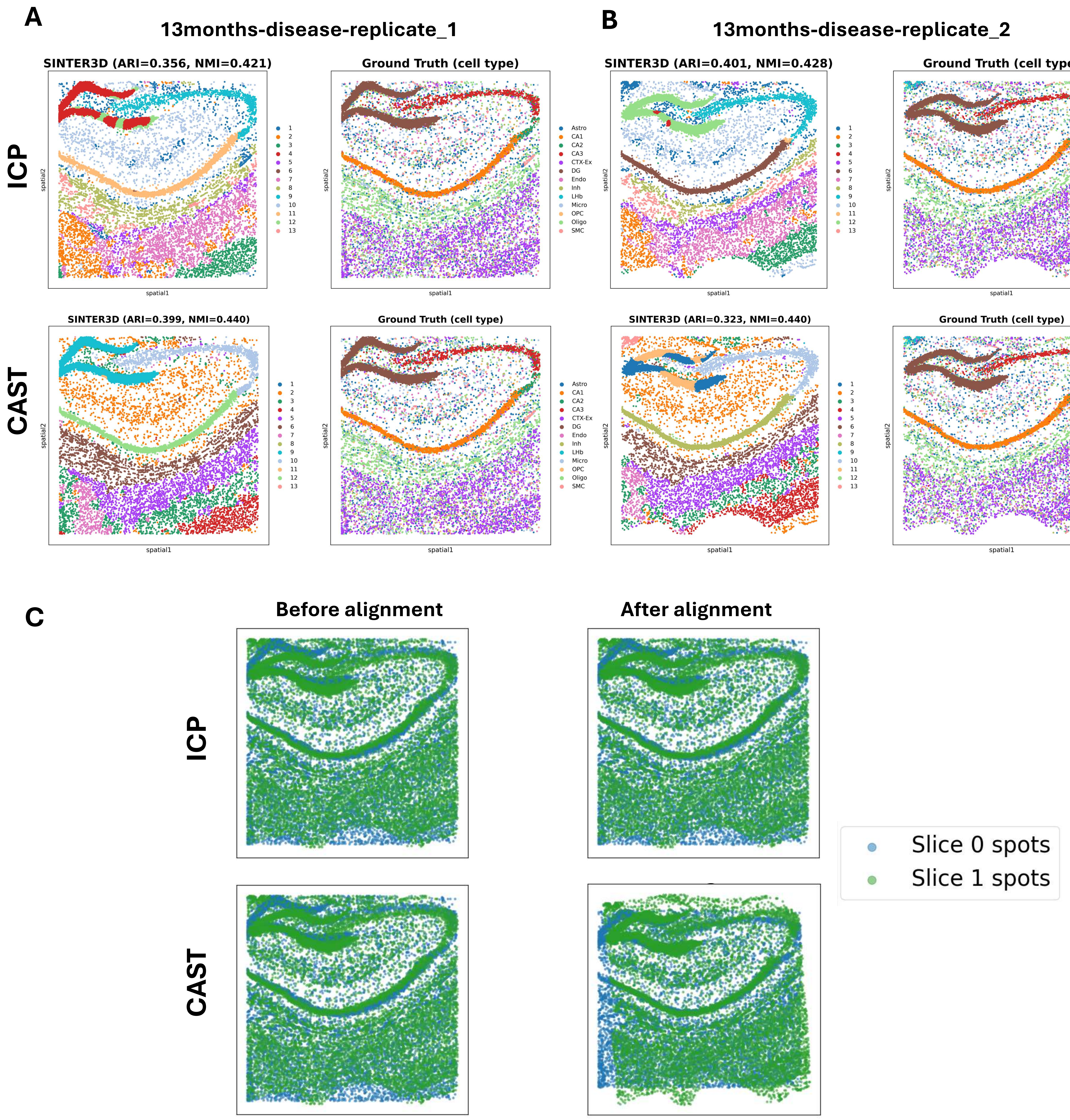
A
13months-disease-replicate_1
B
13months-disease-replicate_2
ICP
CAST
SINTER3D (ARI=0.356, NMI=0.421)
Ground Truth (cell type)
SINTER3D (ARI=0.401, NMI=0.428)
SINTER3D (ARI=0.399, NMI=0.440)
SINTER3D (ARI=0.323, NMI=0.440)
spatial1
spatial2
1
2
3
4
5
6
7
8
9
10
11
12
13
Astro
CA1
CA2
CA3
CTX-Ex
DG
Endo
Inh
LHb
Micro
OPC
Oligo
SMC
C
Before alignment
After alignment
Slice 0 spots
Slice 1 spots

Fig S20. Comparison of ICP- and CAST-based alignment for SINTER3D on the STARmap PLUS dataset.

A–B, SINTER3D clustering results on two 13-month disease STARmap PLUS replicates after ICP- or CAST-based alignment. For each replicate, the left panels show the SINTER3D clustering results, and the right panels show the corresponding ground-truth cell-type annotations. ARI and NMI values are reported above each SINTER3D result.

A, Results for 13months-disease-replicate_1.

B, Results for 13months-disease-replicate_2.

C, Visualization of spatial alignment between two adjacent sections before and after alignment using ICP or CAST. Blue and green points represent cells from two neighboring sections. CAST produced improved spatial correspondence between adjacent sections compared with ICP, while downstream SINTER3D analysis showed comparable or improved agreement with ground-truth cell-type annotations. These results demonstrate that SINTER3D is compatible with non-rigid CAST alignment and can be applied to single-cell-resolution spatial transcriptomics data.

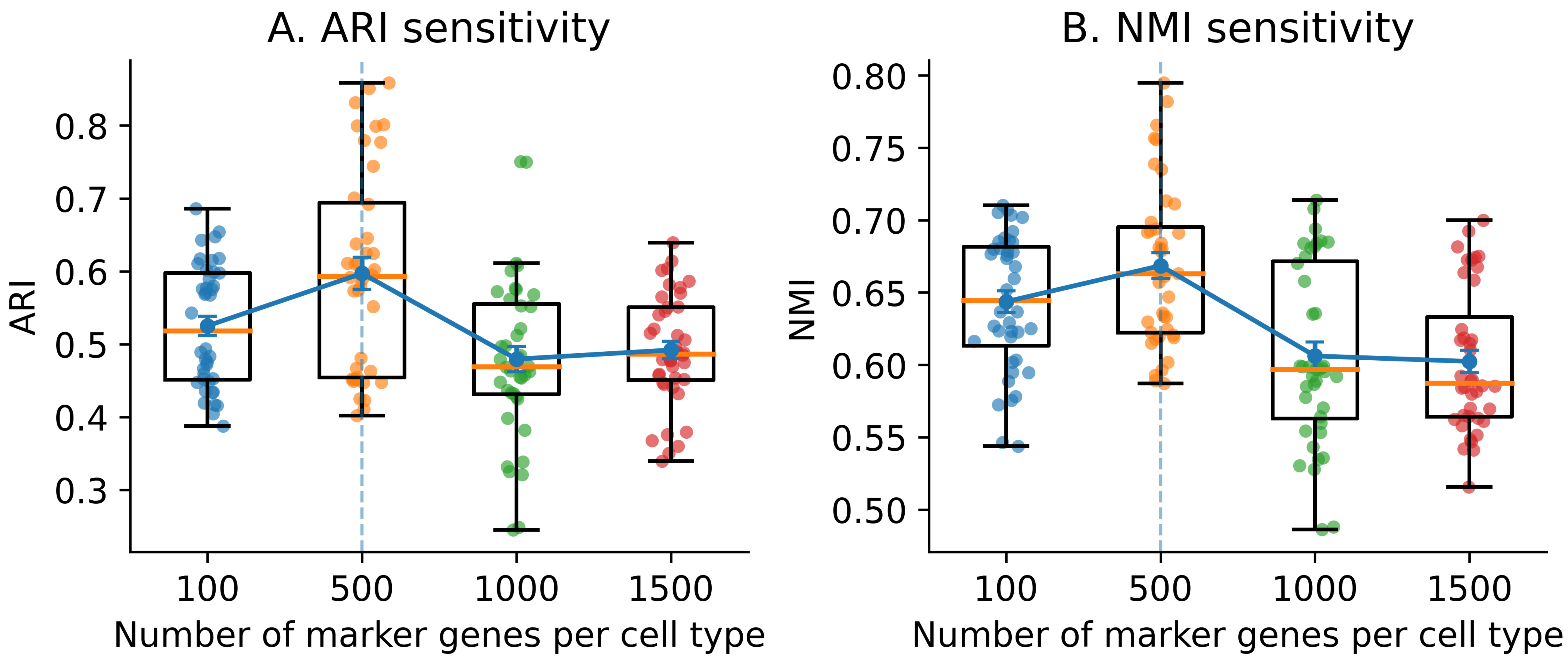

Fig S21. Sensitivity analysis of the number of marker genes per cell type on the DLPFC #151673–#151676 dataset.
SINTER3D was evaluated under K=100,500,1000, and 1500, where K denotes the number of marker genes selected for each cell type from the scRNA-seq reference. ARI and NMI were used to evaluate spatial domain identification performance across multiple random seeds and slices. K=500 achieved the highest average performance, supporting its use as the default setting.

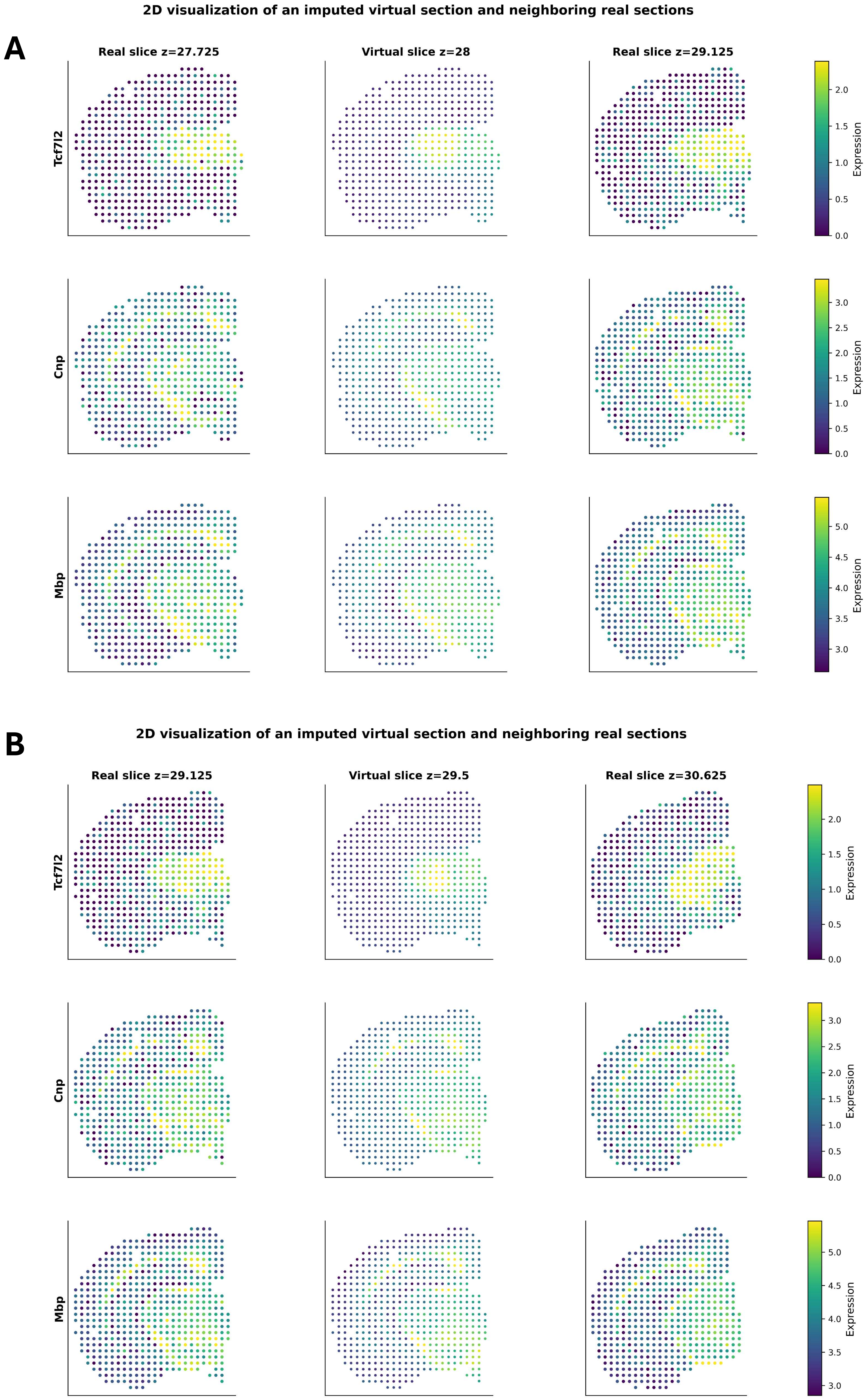
2D visualization of an imputed virtual section and neighboring real sections
A
Real slice z=27.725
Virtual slice z=28
Real slice z=29.125
Tcf7l2
Cnp
Mbp
Expression
0.0
0.5
1.0
1.5
2.0
2.5
3.0
3.5
4.0
4.5
5.0
2D visualization of an imputed virtual section and neighboring real sections
B
Real slice z=29.125
Virtual slice z=29.5
Real slice z=30.625
Tcf7l2
Cnp
Mbp
Expression

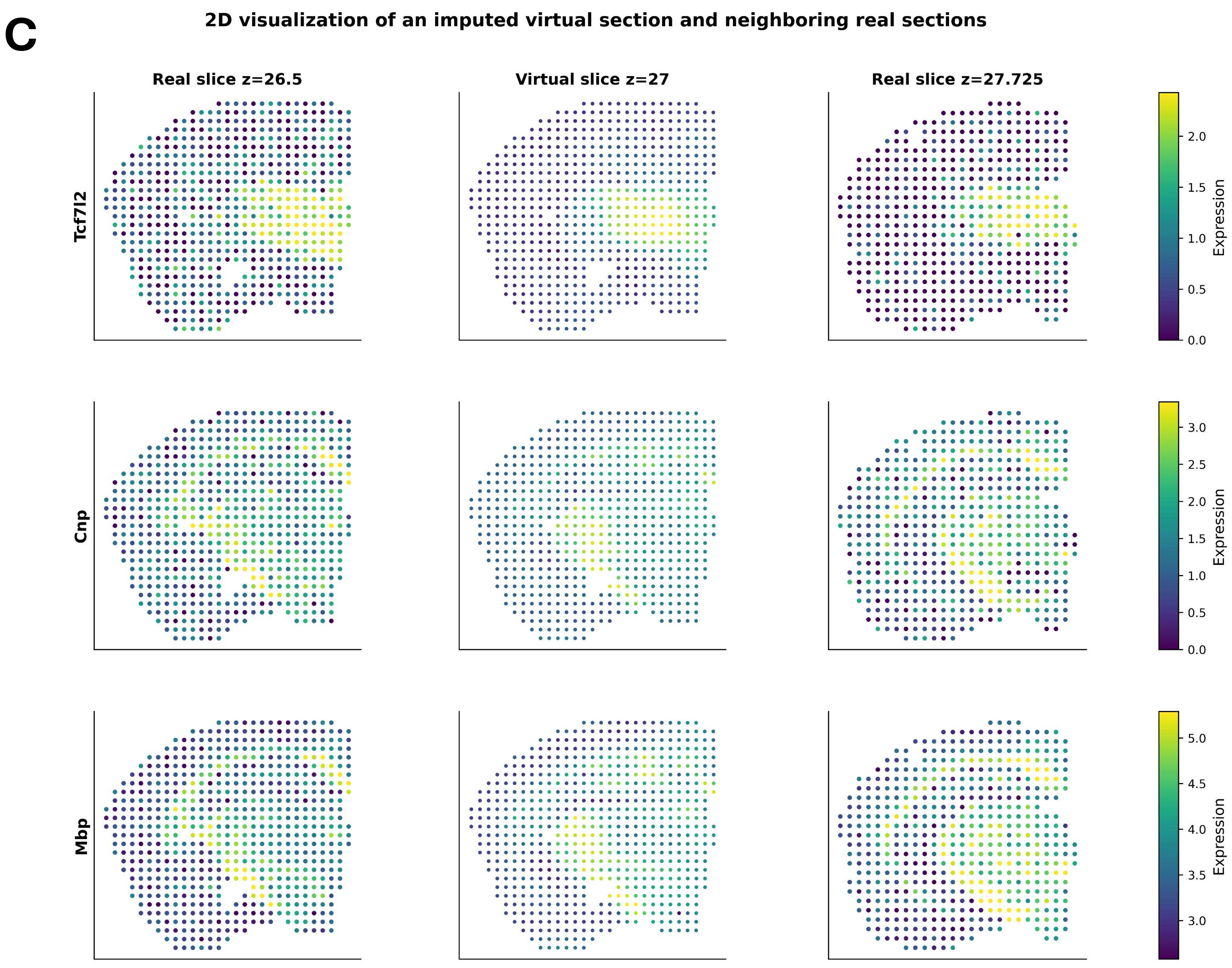

Fig S22. 2D visualization of a representative imputed mouse brain section generated by SINTER3D.

A–C, Two-dimensional expression maps of representative marker genes in neighboring measured sections and the SINTER3D-imputed virtual section. For each gene, the left and right panels show the measured sections adjacent to the target z position, and the middle panel shows the imputed virtual section.

The imputed spot coordinates were generated using the geometry-aware virtual-section construction strategy described in Methods, and expression values were predicted by the trained SINTER3D model. The reconstructed expression patterns in the virtual section show spatial continuity with neighboring measured sections and are consistent with known mouse brain anatomical structures.

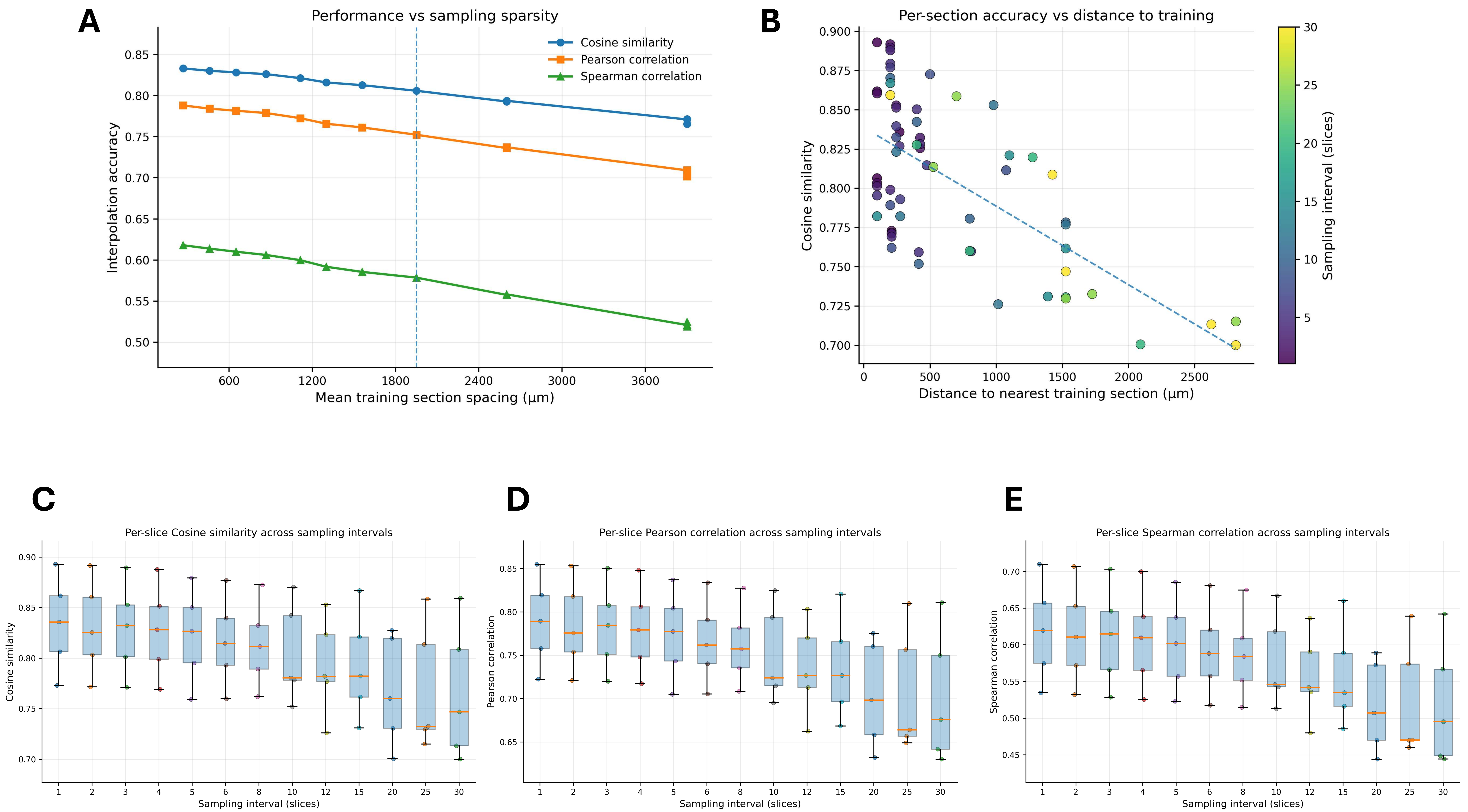

Fig S23. Effect of z-axis sampling density on SINTER3D interpolation performance in the adult mouse brain dataset.

A, Overall interpolation performance of SINTER3D under different z-axis sampling densities. The x-axis indicates the mean spacing between training sections, and the y-axis shows interpolation accuracy measured by Cosine similarity, Pearson correlation, and Spearman correlation.

B, Relationship between the distance from each held-out test section to its nearest training section and the corresponding interpolation accuracy, measured by Cosine similarity. Each point represents one held-out test section under a specific sampling interval.

C–E, Per-section performance distributions under different sampling intervals. Boxplots show the distributions of **C**, Cosine similarity, **D**, Pearson correlation, and **E**, Spearman correlation across held-out test sections. These results show that SINTER3D maintains robust interpolation performance under moderately sparse z-axis sampling, while prediction accuracy and stability gradually decrease as the sampling interval increases.

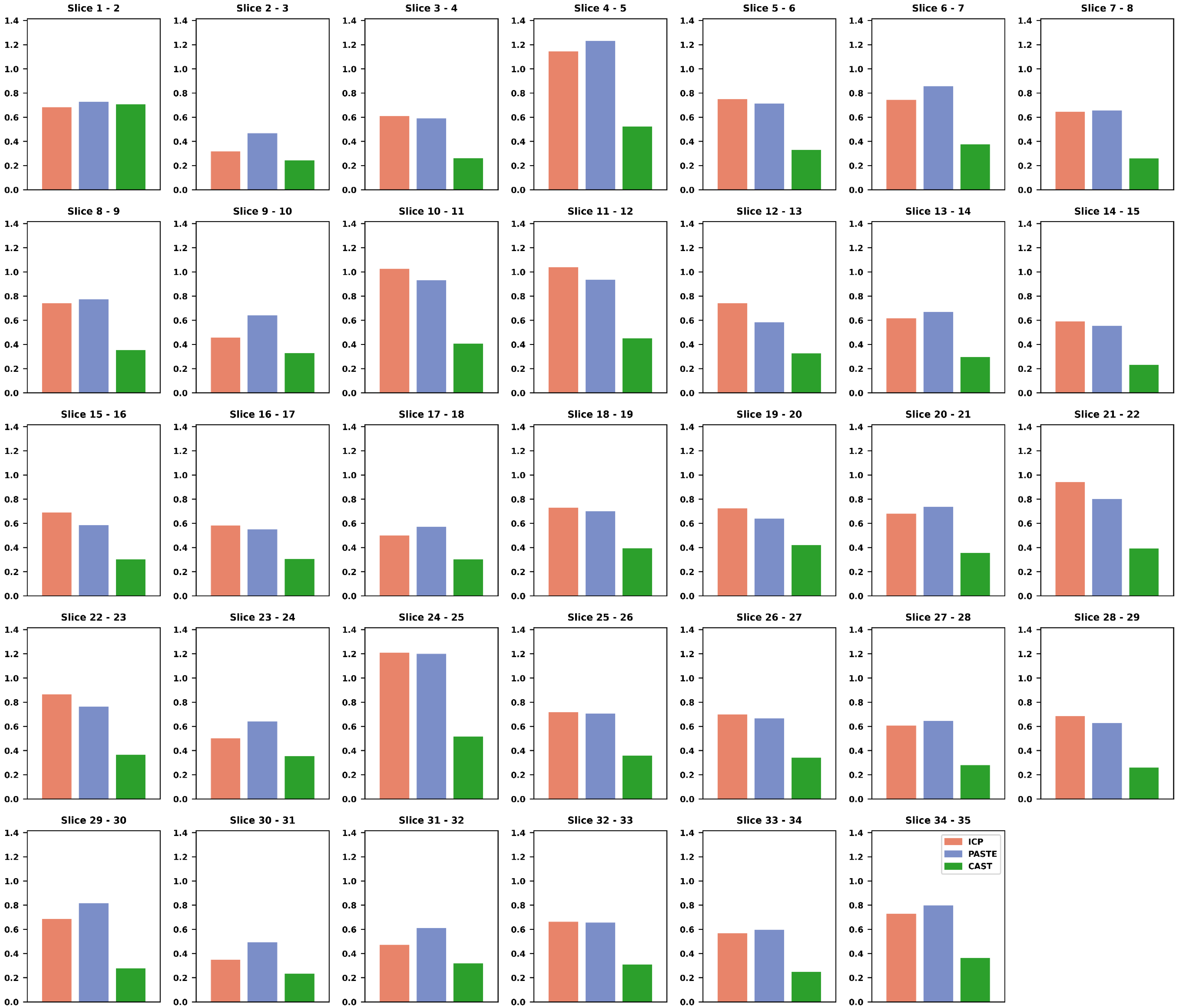

Fig S24. Quantitative evaluations of alignment accuracy for ICP, PASTE, and CAST using 34 pairs of mouse brain tissue slices. For each spot in slice A, we calculated the minimal distance between the chosen spot and spots having the same region label with the chosen spot in slice B. Then the score was obtained by averaging these minimal distances over all spots in the two slices. A smaller value indicates a better performance.

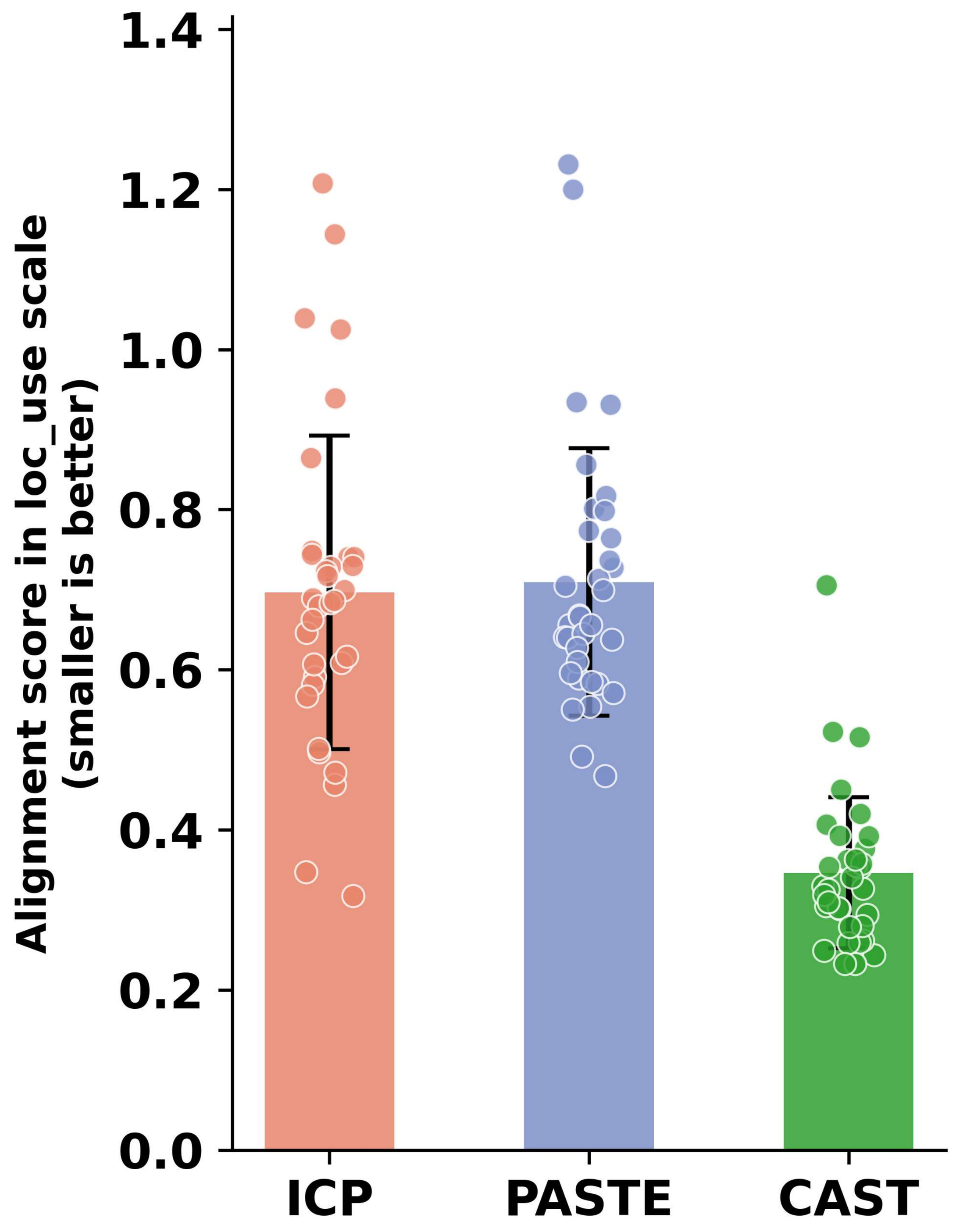

Fig S25. Overall alignment accuracy for ICP, PASTE, and CAST using mouse brain data. The overall score was computed by taking the mean of scores in Fig. S24 over all pairs. Error bars indicate standard deviations of the scores. Data are presented as mean values +/- standard deviations, calculated based on n = 34 points.

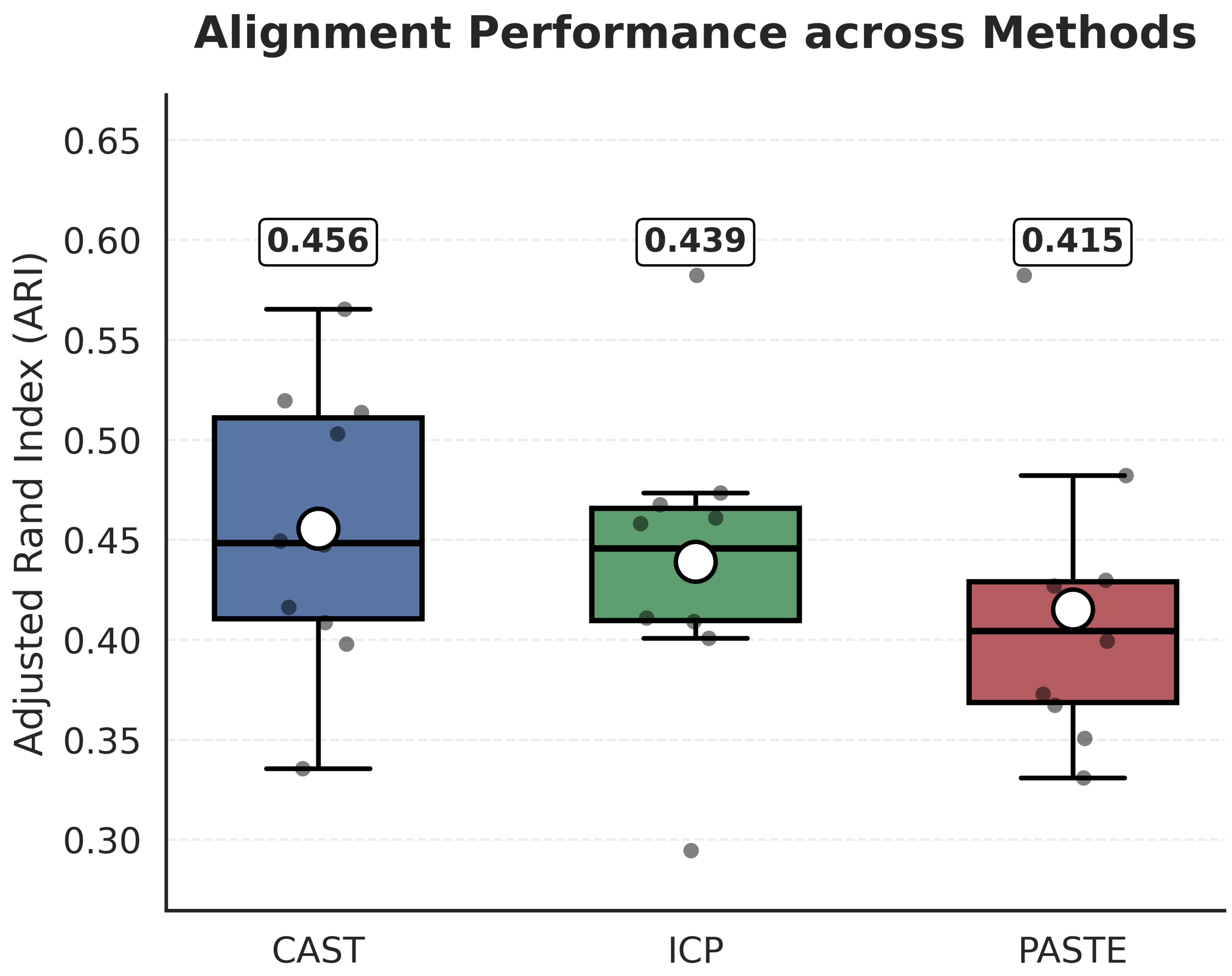

Fig S26. Downstream SINTER3D spatial domain clustering performance after applying different alignment methods. CAST-based alignment achieved the highest mean ARI, followed by ICP and PASTE, indicating that improved non-rigid alignment can benefit downstream SINTER3D analysis.

Supplementary Section 1: Impact of Marker Gene Count (*K*) on Model Performance

To evaluate the impact of the number of marker genes selected per cell type *K* on model performance, we performed a sensitivity analysis on the DLPFC #151673–#151676 datasets, comparing four settings: *K* = 100, 500, 1000, and 1500. For each setting, we assessed the spatial domain identification performance of SINTER3D using the Adjusted Rand Index (ARI) and Normalized Mutual Information (NMI) across multiple random seeds and sections. The results show that *K* = 500 achieves the best overall performance on these datasets, yielding the highest mean ARI and NMI among all tested settings. In contrast, smaller *K* values may fail to retain sufficient cell-type-specific information, whereas larger *K* values do not further improve performance and may instead introduce more low-information or noisy genes. These findings indicate that *K* = 500 offers a favorable balance among biological information retention, model stability, and computational efficiency, and it was therefore adopted as the default setting for SINTER3D (see Additional file 3: Fig S21).

Supplementary Section 2 :Data-Driven Submodel Selection Strategy

1.1 Background and Motivation

To accommodate the inherent high heterogeneity of spatial transcriptomics datasets, SINTER3D incorporates three specialized submodels: Basic, Spatial, and Complex. Manually selecting the optimal submodel for a new dataset requires domain expertise and is computationally inefficient. To automate and data-drive this process, we developed a lightweight random forest classifier. This classifier employs two intrinsic, dataset-level features to predict the best-performing submodel:

Number of sections: $k = |\text{unique}(\boldsymbol{S})|$ which represent the spatial depth and continuity of the dataset.

Expression heterogeneity: $h = \frac{1}{G}\sum_{g=1}^{G} \text{Var}(\boldsymbol{X}_{:,g})$ Quantifying the overall spatial variability of gene expression.

We intentionally opted for a simple model (Random Forest) and a highly informative yet condensed set of features to guard against overfitting and ensure robust performance, even with limited initial training data.

1.2 Training Label Assignment via Cross-Validation on Representative Datasets

The ground truth labels for the classifier were determined through empirical performance evaluation on four publicly available benchmark datasets. These datasets encompass diverse tissues, organisms, and technological platforms, providing broad representativeness.

For each dataset, the submodel achieving the highest Adjusted Rand Index (ARI) on the held-out validation set was assigned as its pseudo-label. This process ensured that our classifier aims to emulate a cross-validation-based "oracle" selector.

1.3 Classifier Training and Independent Validation

A random forest classifier was trained using the feature-label pairs listed in Table S2. Despite the small training set size (n=4), the classifier learned a coherent decision boundary:

Datasets with few sections and moderate expression heterogeneity (e.g., DLPFC) were best assigned to the Basic model.

Datasets with numerous sections (e.g., mouse brain) were assigned to the Spatial model to capture multi-scale spatial dependencies.

Datasets with high expression heterogeneity (e.g., Drosophila embryo and breast cancer) were directed to the Complex model, which effectively handles variability through its teacher-student architecture.

To rigorously evaluate the classifier's generalization capability, we performed independent validation using the developing human heart dataset, which was not included in the training set. Based on the cardiac data's features (k=9, h=0.31), the classifier correctly recommended the Complex model. Our experimental validation in the main text confirms that the Complex model indeed achieved superior performance on this dataset, demonstrating the practical utility and robustness of our selection strategy.

We acknowledge the limited size of the initial training set. However, this design represents a deliberate trade-off to achieve a practical, efficient, and scalable automated selection mechanism. Our approach is justified by the following rationales:

Feature Relevance: The two selected features are principled, biologically informative, and strongly correlated with model performance, as demonstrated in Table S3.

Model Simplicity: Using a simple Random Forest with only two features inherently guards against overfitting.

Empirical Success: The classifier exhibits logically sound decision-making, and its performance has been validated on an independent dataset (human heart), confirming its generalization capability beyond the training samples.

Supplementary Section 3: Impact of Sparse Sampling along the Z-Axis on the Performance of SINTER3D

To directly evaluate the impact of sparse sampling along the z-axis on SINTER3D performance, we conducted additional sampling density analyses on an adult mouse brain dataset. Specifically, we designated five held-out sections distributed along the anterior–posterior axis as test sections and progressively reduced the number of training sections along the z-axis to simulate varying degrees of sparse sampling. Consequently, the average inter-section distance of the training set ranged from 269 μm to 3900 μm.

The results show that SINTER3D remains notably robust under moderately sparse sampling conditions. As the average training section spacing increased from 269 μm to 1950 μm, Cosine similarity decreased only from 0.833 to 0.806, Pearson correlation from 0.788 to 0.752, and Spearman correlation from 0.618 to 0.579, indicating a gradual performance decline with increasing sampling sparsity rather than an abrupt collapse. Further reducing the number of training sections to four (average spacing approximately 2,600 μm) led to more pronounced drops, with Cosine similarity around 0.793 and Pearson correlation around 0.736; when only three training sections were retained (average spacing approximately 3,900 μm), the Pearson correlation fell further to roughly 0.70.

Additionally, our per-slice analysis revealed that the prediction accuracy of test sections is tightly coupled to their distance from the nearest training section: the larger the distance, the lower the interpolation accuracy overall. Box plots at different sampling intervals further demonstrated that the variability in performance across test sections increases as sampling becomes sparser. Together, these results indicate that SINTER3D is highly robust to sparse z-axis sampling, but interpolation accuracy and stability gradually degrade when training sections become too few and the inter-section spacing becomes excessively large.

Based on the experimental results from this adult mouse brain dataset, under the empirical criterion of "Cosine similarity > 0.80 and Pearson correlation > 0.75," as few as five training sections spanning a 7.8 mm anterior–posterior extent (corresponding to an average inter-section spacing of approximately 2 mm) are sufficient to support reasonably accurate 3D interpolation. We have incorporated these findings into the revised supplementary materials and added a new supplementary figure, Additional file 3: Fig S23, to systematically illustrate the influence of z-axis sampling density on the interpolation performance of SINTER3D. In this figure, Additional file 3: Fig S23A summarizes the relationship between average training section spacing and overall interpolation performance; Additional file 3: Fig S23B shows the relationship between the distance from a test section to its nearest training section and per-section prediction accuracy; and Additional file 3: Fig S23 C–E present the per-section distributions of Cosine similarity, Pearson correlation, and Spearman correlation at different sampling intervals, further demonstrating that performance variability across test sections gradually increases as sampling becomes sparser.

Supplementary Tables

Table S1: Downstream SINTER3D performance after different alignment methods.

Replicate	Alignment	ARI	NMI
13-months-disease-replicate_1	ICP	0.356	0.421
13-months-disease-replicate_1	CAST	0.399	0.440
13-months-disease-replicate_2	ICP	0.401	0.428
13-months-disease-replicate_2	CAST	0.323	0.440
**Mean**	**ICP**	**0.379**	**0.425**
**Mean**	**CAST**	**0.362**	**0.440**

Table S2: Empirical Performance and Label Assignment of the Submodel Selection Classifier.

**Dataset**	**Basic_model(ARI)**	**Spatial_model(ARI)**	**Complex_model(ARI)**	**Select_Model**
DLPFC	0.596097	0.013739	0.294898	Basic
Mouse_Brain	0.263929	0.439051	0.334725	Spatial
Drosophila	0.123063	0.102096	0.281723	Complex
Her2 breast cancer	0.038106	0.20135	0.279825	Complex

Table S3: Data characteristics of the different datasets.

Dataset	n_slices	Expression complexity	Model
DLPFC	4	0.27	Basic
Mouse_Brain	35	0.12	Spatial
Drosophila	13	0.28	Complex
Human_heart	9	0.31	Complex
Her2 breast cancer	6	0.20	Complex

Table S4: Running time and memory usage of SINTER3D across different datasets.

Datesets	Time(m)	Memory(MB)
Mouse brain	12.4	10341
DLPFC	2.3	5527
Drosophila embryo	3.2	5769
Human heart	2.2	2773
Her2 breast cancer	1.3	3145

Table S5: Implementation details of baseline methods.

Method	Task in this study	Code source / official resource	Parameter setting	Notes
STAGATE	Spatial domain identification	https://github.com/zhanglabtools/STAGATE	Default parameters recommended by the original implementation	Used for spatial clustering/domain identification.
GraphST	Spatial domain identification and comparison analysis	https://github.com/JinmiaoChenLab/GraphST	Default parameters recommended by the original implementation	Used according to the official GraphST workflow.
STitch3D	3D spatial domain identification and cell-type deconvolution	https://github.com/YangLabHKUST/STitch3D	Default parameters recommended by the original implementation	Used for multi-slice 3D modeling and deconvolution comparison.
Tangram	Cell-type mapping deconvolution comparison	https://github.com/broadinstitute/Tangram	Default parameters recommended by the official tutorial	Used with the same scRNA-seq reference where applicable.
CARD	Cell-type deconvolution comparison	https://github.com/YMa-lab/CARD	Default parameters recommended by the original implementation	Used with the same scRNA-seq reference where applicable.
Spateo	3D interpolation comparison	https://github.com/aristoteleo/spateo-release	Default parameters recommended by the official implementation	Used for VTK-based and vector-field-based interpolation comparisons where applicable.
STAIG	Spatial domain identification comparison	https://github.com/y-itao/STAIG	Default parameters recommended by the original implementation	Used according to the official STAIG workflow.
SGCD	Spatial domain identification comparison	https://github.com/hezi-li/SGCD	Default parameters recommended by the original implementation	Used for high-resolution spatial domain characterization comparison.

Table S6: Number of slices, spatial units, cell types, and genes used for each spatial transcriptomics dataset.

ST dataset	# slice	#spatial units	#cell types	#genes
Mouse brain	35	17,086	59	6227
Human DLPFC	4	14,243	18	4558
Drosophila embryo	13	14,132	16	5841
Human heart	9	1470	12	3837
Her2 breast cancer	6	2065	20	4530
STARmap PLUS mouse brain	2	20,006	13	2435

Table S7: Alignment quality comparison.

Methods	Origin	ICP	CAST
cell type concordance	0.327	0.330	0.403
